# Supplementary material for: Is an opportunistic primary care-based intervention for non-responders to bowel screening feasible and acceptable? A mixed-methods feasibility study in Scotland
Source: BMJ Open. 2017 Oct 11;7(10):e016307. doi: 10.1136/bmjopen-2017-016307 (PMC5652541; doi:10.1136/bmjopen-2017-016307)
Supplement: Supplementary file 1 [file bmjopen-2017-016307supp001.pdf]

Supplementary file 1:

A. **Estimating the size of the study population**
B. **Intervention supporting materials**
C. **Feasibility parameters**

A. Estimating the size of the study population

According to the 2011 Census, there are 184,846 residents in Lothian aged 50-74[1] (the age range for eligibility to bowel cancer screening). In these age groups, approximately 86% of patients (estimated 158,968) consult at least once a year (aged 45 and over are included as data were not available separately)[2], with a linear increase in the number of consultations according to patients' age (from 5.2 annual consultations to 7.7 among the most deprived groups).<br>As of September 2013 there were 127 practices in Lothian[3]. Each practice would then have, on average, at least 1252 patients consulting during our intervention period (although numbers vary between practices).<br><br>Official Scottish Bowel screening data for the period 2011-2013 show that uptake in Lothian was 55.3% (ranging from 39.6% to 59.8% from the most deprived to the least deprived areas)[4]. Considering the participation rates for the most deprived areas (39.6%) and the population aged 50-74; there would roughly be a maximum of 756 eligible patients who could be reached during the study period per practice, or 4536 in six selected practices.<br><br>In practice, however, numbers are likely to be much smaller. For example, in 2011 the prevalence of cancer (all types) in Scotland was 4.5%[5]. These patients would not take part in the intervention as it would not be appropriate to approach the issue of cancer screening to someone in receipt of cancer treatment. Considering cancer prevalence in Scotland, the number of eligible patients would be reduced to 4355 in all practices. This is a conservative estimate as cancer is more common in areas of high deprivation (which is the case of some of the selected practices in Lothian). Furthermore, the opportunity to approach a patient about screening may not arise in every consultation. We estimate that an opportunity will arise in about a quarter of all consultations with eligible participants, thus reducing the number of patients to 1089 (or approximately 182 per practice).

References:

1.Scotland's Census 2011 - National Records of Scotland. Table DC1117SC - Age by sex. All people [Internet]. 2014. Available from: http://www.scotlandscensus.gov.uk.

2.Estimated number of patients seen1/not seen2 in Scotland by either a General Practitioner (GP) or Practice Nurse [Internet]. 2013. Available from: http://www.isdscotland.org/PTI/.

3 .General Practice – GP workforce and practice population statistics to 2013. Scottish general practice characteristics: as at 30th September from 2005 to 2013. In: ISD Scotland, editor. 2014.

4.Scottish Bowel Screening Programme. Scottish Bowel Screening Programme. Key Performance Indicators Report: May 2014 data submission. Invitations between 1st November 2011 and 31st October 2013. Scotland: Scottish Bowel Screening Programme; 2014.

5. ISD Scotland. Cancer in Scotland: ISD Scotland; 2012. Available from: https://isdscotland.scot.nhs.uk/Health-Topics/Cancer/Publications/2012-04-24/Cancer_in_Scotland_summary_m.pdf

B. Intervention supporting materials

Intervention material	Aim(s)	Theoretical framework/adopted principles
3-4 questions/statements<br>• A5 coloured laminated sheet of paper<br>• Given to the health care professional	• To guide discussion addressing concerns around or barriers to bowel screening with non-responders	• Non-directive statements<br>• Non-coercion<br>• Informed choice<br>• Implementation Intentions theory
Staff flowchart<br>• A4 coloured laminated sheet of paper<br>• Given to the health care professional	• To describe barriers to screening (with examples), and evidence-based approaches to deal with these barriers	• Health Behaviour Framework<br>• Motivational Interviewing
Patient leaflet and freepost envelope<br>• A4 coloured sheet of paper folded into A5, perforated so a reply slip could be sent back by post<br>• FREEPOST envelope addressed to the bowel screening centre<br>• Given to patients who accepted the intervention	• To deconstruct health beliefs associated with low uptake, i.e. the barriers described in the flowchart<br>• To provide space for the patient to develop their own implementation plan<br>• To offer the opportunity to request a new test kit	• Health Behaviour Framework<br>• Non-coercion<br>• Informed choice<br>• Implementation Intentions theory
Guidance sheet<br>• A4 coloured laminated sheet of paper<br>• Given to the health care professional	• To provide practice staff with relevant information regarding the research study	N/A
Practice Proforma<br>• Green A4 sheet with perforations (3 proformas in each)<br>• Given to the health care professional<br>• Designed to require approximately 2 minutes to be completed	• To collect relevant intervention data so the feasibility outcomes could be assessed:<br>o Intervention date, staff name and role, duration of the intervention, patient age and gender, reasons for consultation (text)<br>o Whether intervention was accepted, leaflet was given and completed in practice<br>o Comments	N/A

Note: Bowel Screening leaflets in Polish were requested from NHS Scotland and provided to practices with a larger Polish population

C. Feasibility parameters

- Processes required in general practice to record (and flag) responder status in patients’ electronic records
- Number of patients approached during consultations
- Number of interventions delivered over the intervention period
- Length of time the intervention takes
- Number and willingness of patients to engage in conversation when responder status is raised during a consultation
- Number of patients who were willing to receive leaflet
- Number of patients who completed leaflet with health care professional
- Number of patients who sent reply slip
- Number of patients who returned a completed kit
- Processes required in the bowel screening centre to deal with the extra-to-programme returned FOBt kits, and to ensure over-screening does not occur
- Whether the brief intervention leads to longer discussion with patients regarding cancer screening, or bowel symptoms
- Willingness of GPs and practice nurses to deliver intervention
- Overall views on the study’s feasibility according to primary care professionals
- Overall views on the study’s feasibility according to bowel screening staff
