# Supplementary material for: Is an opportunistic primary care-based intervention for non-responders to bowel screening feasible and acceptable? A mixed-methods feasibility study in Scotland
Source: BMJ Open. 2017 Oct 11;7(10):e016307. doi: 10.1136/bmjopen-2017-016307 (PMC5652541; doi:10.1136/bmjopen-2017-016307)
Supplement: Supplementary file 2 [file bmjopen-2017-016307supp002.pdf]

Supplementary file 2

A. Recruitment Flowchart

B. Characteristics of practices approached in Wave 1

A. Recruitment Flowchart

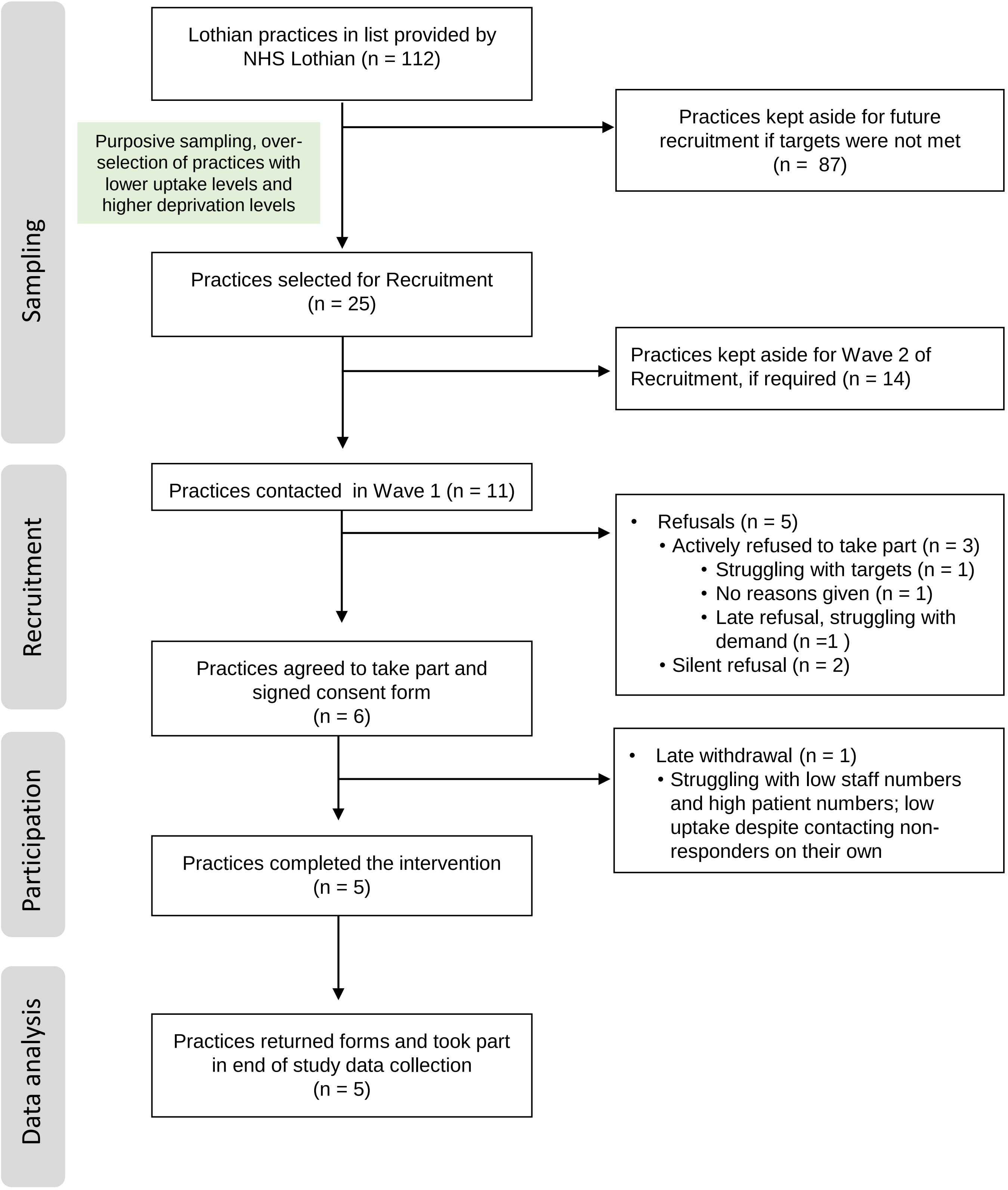

B. Characteristics of practices approached in Wave 1

Practice name	Outcome in Wave 1 of recruitment	Uptake % (2013)	Pop 50-75	Mean SIMD decile* (50-75yr olds)	Average Monthly Non Responder Numbers	Practice list size (ISD)
Practice A	Recruited	<45%	1,413	2.6	41	6,888
Practice B	Recruited	45-50%	2,654	3.6	61	10,440
Practice C	Recruited	50-55%	2,515	4.5	56	8,693
Practice D	Recruited	50-55%	1,241	6.2	29	5,326
Practice E	Recruited	55-60%	1,668	5.5	30	5,201
Practice F	Recruited, but withdrew as struggling with pressures	50-55%	3,444	3.2	73	11,624
Practice G	Actively refused, struggling with pressures	<45%	2,543	2.1	70	12,482
Practice H	Actively refused, struggling with pressures	45-50%	2,030	4.2	47	9,585
Practice I	Actively refused, no reasons given	50-55%	3,809	4.6	76	13,984
Practice J	Silent refusal	<45%	1,075	3.9	29	7,848
Practice K	Silent refusal	45-50%	2,241	3.4	50	8,287

Abbreviations: ISD: Information Services Division; Pop: population; SIMD: Scottish Index of Multiple Deprivation.
*The lower the decile number, the higher the deprivation levels.
