# Supplementary material for: Is an opportunistic primary care-based intervention for non-responders to bowel screening feasible and acceptable? A mixed-methods feasibility study in Scotland
Source: BMJ Open. 2017 Oct 11;7(10):e016307. doi: 10.1136/bmjopen-2017-016307 (PMC5652541; doi:10.1136/bmjopen-2017-016307)
Supplement: Supplementary file 3 [file bmjopen-2017-016307supp003.pdf]

Supplementary file 3:

A. Recorded reasons for consultation
B. Comparing patients who accepted and did not accept the intervention

A. Recoded reasons for consultation

Reasons for consultation	Practice A n(%)	Practice B n(%)	Practice C n(%)	Practice D n(%)	Practice E n(%)	All practices n(%)			
	**All**	**All**	**All**	**All**	**All**	**GP**	**PN**	**HCA**	**All**
Known chronical illness/review of existing condition	25 (36.8)	6 (11.5)	2 (7.4)	18 (26.1)	4 (11.8)	12 (21.8)	40 (72.7)	3 (5.5)	55 (22.0)
Musculoskeletal symptoms/conditions	3 (4.4)	7 (13.5)	7 (25.9)	11 (15.9)	5 (14.7)	33 (100.0)	0 (0.0)	0 (0.0)	33 (13.2)
Tests/test results	8 (11.8)	8 (15.4)	3 (11.1)	8 (11.6)	2 (5.9)	4 (13.8)	17 (58.6)	8 (27.6)	29 (11.6)
Respiratory or ENT symptoms/conditions	6 (8.8)	7 (13.5)	2 (7.4)	4 (5.8)	3 (8.8)	14 (63.6)	8 (36.4)	0 (0.0)	22 (8.8)
Prescriptions/medication review	7 (10.3)	2 (3.8)	1 (3.7)	1 (1.4)	4 (11.8)	15 (100.0)	0 (0.0)	0 (0.0)	15 (6.0)
Skin complaints	1 (1.5)	5 (9.6)	2 (7.4)	3 (4.3)	4 (11.8)	13 (86.7)	2 (13.3)	0 (0.0)	15 (6.0)
Non-clinical	2 (2.9)	4 (7.7)	2 (7.4)	5 (7.2)	1 (2.9)	10 (71.4)	2 (14.3)	2 (14.3)	14 (5.6)
Gastrointestinal symptoms/conditions	3 (4.4)	4 (7.7)	1 (3.7)	4 (5.8)	2 (5.9)	14 (100.0)	0 (0.0)	0 (0.0)	14 (5.6)
Multiple issues	4 (5.9)	1 (1.9)	0 (0.0)	1 (1.4)	5 (14.7)	11 (100.0)	0 (0.0)	0 (0.0)	11 (4.4)
Mental health symptoms/conditions	0 (0.0)	2 (3.8)	2 (7.4)	2 (2.9)	1 (2.9)	7 (100.0)	0 (0.0)	0 (0.0)	7 (2.8)
Preventative behaviour	4 (5.9)	1 (1.9)	2 (7.4)	0 (0.0)	0 (0.0)	0 (0.0)	7 (100.0)	0 (0.0)	7 (2.8)
Gynaecological or urological symptoms/conditions	2 (2.9)	1 (1.9)	0 (0.0)	1 (1.4)	0 (0.0)	4 (100.0)	0 (0.0)	0 (0.0)	4 (1.6)
Inconclusive	0 (0.0)	0 (0.0)	0 (0.0)	2 (2.9)	0 (0.0)	2 (100.0)	0 (0.0)	0 (0.0)	2 (0.8)
Other/non-specific symptoms	3 (4.4)	4 (7.7)	3 (11.1)	9 (13.0)	3 (8.8)	21 (95.5)	1 (4.5)	0 (0.0)	22 (8.8)
Total	68 (100.0)	52 (100.0)	27 (100.0)	69 (100.0)	34 (100.0)	N/A	N/A	N/A	250 (100.0)

Abbreviations: GP: General Practitioner; PN: Practice Nurse; HCA – Health Care Assistant; ENT – ear, nose and throat
Missing data: 8 cases (3 in Practice A, 1 in Practice C, 1 in Practice D and 3 in Practice B).
Sums may not add up to 100 due to rounding

B. Comparing patients who accepted and did not accept the intervention

Overall data	Accepted the intervention (n=220) n(%)	Did not accept the intervention (n=33) n(%)	Overall (n=253)* n(%)
**Patient sex**			
Male	113 (51.4)	22 (66.7)	135 (53.4)
Female	107 (48.6)	11 (33.3)	118 (46.6)
**Patient age**			
Median (IQR)	58.00 (53.00-64.75)	64.00 (57.00-71.50)	58.00 (53.00-65.00)
50-54	78 (35.5)	3 (9.1)	81 (32.5)
55-59	48 (21.8)	9 (27.3)	57 (22.9)
60-64	36 (16.4)	6 (18.2)	42 (16.9)
65-69	32 (14.5)	3 (9.1)	35 (14.1)
70-74	17 (7.7)	9 (27.3)	26 (10.4)
75-79	5 (2.3)	3 (9.1)	8 (3.2)
**Staff carrying out the intervention**			
GP	153 (69.5)	11 (33.3)	164 (64.8)
PN	57 (25.9)	19 (57.6)	76 (30.0)
HCA	10 (4.5)	3 (9.1)	13 (5.1)
**Duration of the intervention**			
Median (IQR)	2.00 (1.00-5.00)	2.00 (2.00-5.00)	2.00 (1.00-5.00)
**Reasons for consultation**			
Known chronical illness/review of existing condition	42 (19.7)	12 (37.5)	54 (22.0)
Musculoskeletal symptoms/conditions	30 (14.1)	3 (9.4)	33 (13.5)
Tests/test results	22 (10.3)	5 (15.6)	27 (11.0)
Respiratory or ENT symptoms/conditions	17 (8.0)	5 (15.6)	22 (9.0)
Prescriptions/medication review	15 (7.0)	0 (0.0)	15 (6.1)
Skin complaints	15 (7.0)	0 (0.0)	15 (6.1)
Non-clinical	14 (6.6)	0 (0.0)	14 (5.7)
Gastrointestinal symptoms/conditions	12 (5.6)	1 (3.1)	13 (5.3)
Multiple issues	10 (4.7)	1 (3.1)	11 (4.5)
Mental health symptoms/conditions	6 (2.8)	1 (3.1)	7 (2.9)
Preventative behaviour	5 (2.3)	1 (3.1)	6 (2.4)
Gynaecological or urological symptoms/conditions	2 (0.9)	2 (6.3)	4 (1.6)
Inconclusive	2 (0.9)	0 (0.0)	2 (0.8)
Other/non-specific symptoms	21 (9.9)	1 (3.1)	22 (9.0)

Abbreviations: GP: General Practitioner; PN: Practice Nurse; HCA – Health Care Assistant; ENT – ear, nose and throat; IQR – Interquartile range

*In 5 cases data were missing on whether patient accepted the intervention. These cases are not included here; hence the overall values do not match the ones in the main manuscript (i.e. 258 participants). There were also missing data for patient age (4 cases), duration of intervention (9 cases) and reasons for consultation (8 cases). Sums may not add up to 100 due to rounding.
