# Supplementary figures and images for: Is an opportunistic primary care-based intervention for non-responders to bowel screening feasible and acceptable? A mixed-methods feasibility study in Scotland
Source: BMJ Open. 2017 Oct 11;7(10):e016307. doi: 10.1136/bmjopen-2017-016307 (PMC5652541; doi:10.1136/bmjopen-2017-016307)

Supplementary file 4

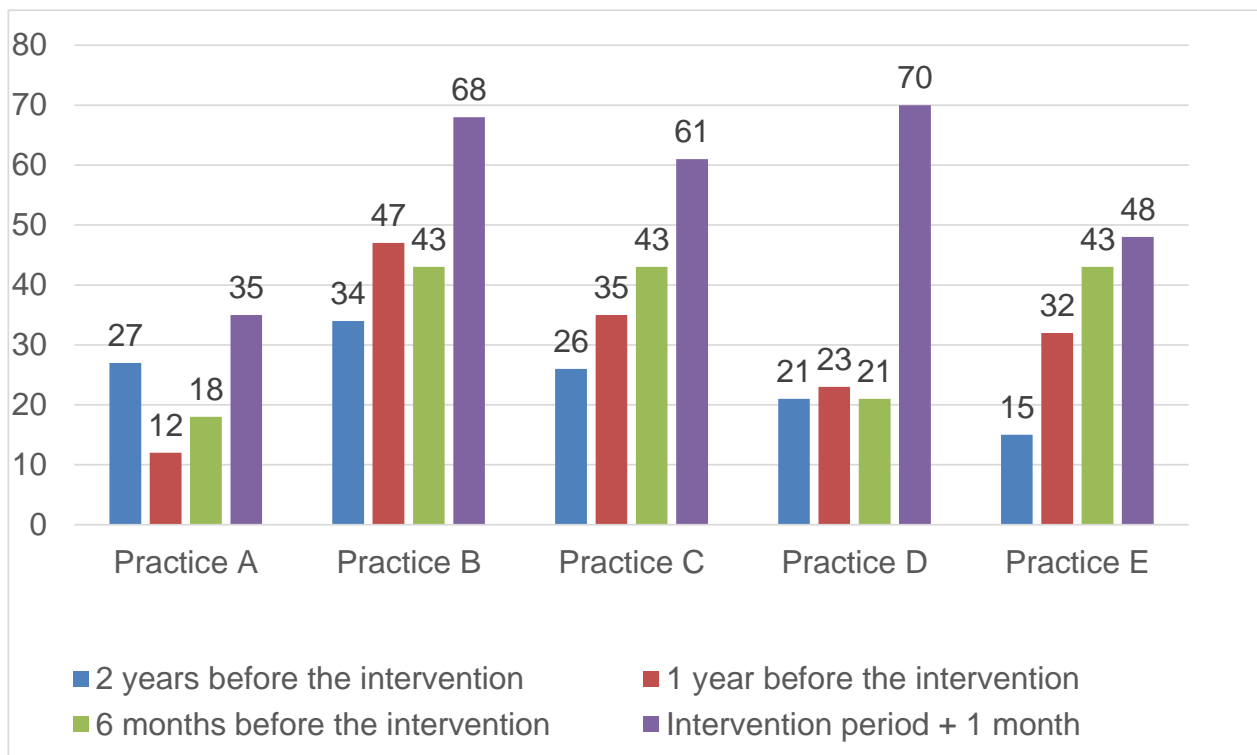

Supplement: Supplementary file 4 [file bmjopen-2017-016307supp004.pdf]
