# Supplementary material for: Is an opportunistic primary care-based intervention for non-responders to bowel screening feasible and acceptable? A mixed-methods feasibility study in Scotland
Source: BMJ Open. 2017 Oct 11;7(10):e016307. doi: 10.1136/bmjopen-2017-016307 (PMC5652541; doi:10.1136/bmjopen-2017-016307)
Supplement: Author's manuscript [file bmjopen-2017-016307.draft_revisions.pdf]

BMJ Open is committed to open peer review. As part of this commitment we make the peer review history of every article we publish publicly available.

When an article is published we post the peer reviewers' comments and the authors' responses online. We also post the versions of the paper that were used during peer review. These are the versions that the peer review comments apply to.

The versions of the paper that follow are the versions that were submitted during the peer review process. They are not the versions of record or the final published versions. They should not be cited or distributed as the published version of this manuscript.

BMJ Open is an open access journal and the full, final, typeset and author-corrected version of record of the manuscript is available on our site with no access controls, subscription charges or pay-per-view fees (http://bmjopen.bmj.com).

If you have any questions on BMJ Open's open peer review process please email info.bmjopen@bmj.com

BMJ Open

Feasibility and acceptability testing of an opportunistic primary care-based intervention for non-responders to bowel screening

Journal:	*BMJ Open*
Manuscript ID	bmjopen-2017-016307
Article Type:	Research
Date Submitted by the Author:	06-Feb-2017
Complete List of Authors:	Calanzani, Natalia ; University of Edinburgh, Centre For Population Health Sciences, The Usher Institute of Population Health Sciences and Informatics<br>Cavers, Debbie; University of Edinburgh, General Practice<br>Vojt, Gabriele; Glasgow Caledonian University, Department of Psychology<br>Orbell, Sheina; University of Essex, Department of Psychology<br>Steele, Robert; University of Dundee, Surgery and Molecular Oncology<br>Brownlee, Linda; Scottish Bowel Screening Centre<br>Smith, Steve; University Hospitals of Coventry & Warwickshire NHS Trust, Midlands & NW Bowel Cancer Screening Hub, Hospital of St. Cross<br>Patnick, Julietta; University of Oxford, Cancer Epidemiology Unit<br>Weller, David; University of Edinburgh, General Practice<br>Campbell, Christine; University of Edinburgh, Centre for Population Health Sciences
**Primary Subject Heading**:	Oncology
Secondary Subject Heading:	General practice / Family practice, Health services research
Keywords:	feasibility studies, bowel screening, general practice, neoplasms

SCHOLARONE™
Manuscripts

Feasibility and acceptability testing of an opportunistic primary care-based intervention for non-responders to bowel screening

Natalia Calanzani[1], Debbie Cavers[1], Gabriele Vojt[2], Sheina Orbell[3], Robert JC Steele[4], Linda Brownlee[5], Steve Smith[6], Julietta Patnick[7], David Weller[1], Christine Campbell[1]

[1]University of Edinburgh, The Usher Institute of Population Health Sciences and Informatics, Centre for Population Health Sciences, Medical School, Teviot Place, Edinburgh EH8 9AG, UK

[2]Glasgow Caledonian University, Department of Psychology, Cowcaddens Road, Glasgow G4 0BA

[3]Department of Psychology, University of Essex, Wivenhoe Park, Colchester CO4 3SQ, UK

[4]Surgery and Molecular Oncology, Ninewells Hospital, University of Dundee, Dundee, DD1 9SY, Scotland, UK

[5]Scottish Bowel Screening Centre, Kings Cross, Clepington Road, Dundee, DD3 8EA, Scotland, UK

[6]University Hospitals of Coventry & Warwickshire NHS Trust, Midlands & NW Bowel Cancer Screening Hub, Hospital of St. Cross, Barry Road, Rugby, CV22 5PX

[7]Oxford University, Cancer Epidemiology Unit, Richard Doll Building, Roosevelt Drive, Oxford OX3 7LF

Word count: 3,988

Corresponding author:

Natalia Calanzani

Centre for Population Health Sciences

The Usher Institute of Population Health Sciences and Informatics, University of Edinburgh

Room 123, Doorway 1, Medical School, Teviot Place, Edinburgh EH8 9AG

Tel: 0131 650 3818

Fax: 031 650 3227

Natalia.Calanzani@ed.ac.uk

For peer review only

For peer review only - http://bmjopen.bmj.com/site/about/guidelines.xhtml

ABSTRACT

Objectives: We aimed to test whether a brief, opportunistic intervention in general practice was a feasible and acceptable way to engage with bowel screening non-responders.

Design: This was a feasibility study testing an intervention which comprised a brief conversation during routine consultation, provision of a patient leaflet and instructions to request a replacement faecal occult blood test kit. A mixed methods approach to evaluation was adopted. Data were collected from proformas completed after each intervention, from the Bowel Screening Centre database, and from questionnaires. Semi-structured interviews were carried out. We used descriptive statistics, content and framework analysis to determine intervention feasibility and acceptability.

Participants: Bowel screening non-responders (as defined by the Scottish Bowel Screening Centre) and primary care professionals working in five general practices in Lothian, Scotland.

Primary and secondary outcome measures: Several predefined feasibility parameters were assessed, including numbers of patients engaging in conversation, requesting a replacement kit and returning it; and willingness of primary care professionals to deliver the intervention.

Results: The intervention was offered to 258 patients in five general practices: 220 (87.0%) engaged with the intervention, 60 (23.3%) requested a new kit, 22 (8.5%) kits were completed and returned. Interviews and questionnaires suggest that the intervention was feasible, acceptable, and consistent with an existing health prevention agenda. Reported challenges referred to work-related pressures, time constraints and practice priorities.

Conclusions: This intervention was acceptable and resulted in a modest increase in non-responders participating in bowel screening, although outlined challenges may affect sustained implementation. The strategy is also aligned with the increasing role of primary care in promoting bowel screening.

Keywords: feasibility studies; bowel screening; general practice; neoplasms

For peer review only - http://bmjopen.bmj.com/site/about/guidelines.xhtml

STRENGTHS AND LIMITATIONS OF THE STUDY

- We have tested the feasibility and acceptability of an evidence-based intervention consisting of a brief conversation targeting non-responders to bowel screening
- The intervention was grounded in the pragmatic reality of a dynamic, time-pressured primary care environment
- This is a small-scale scale feasibility study in one region of Scotland, targeting non-responders who consult in primary care
- As the intervention is of an opportunistic nature, data on patient characteristics (such as medical history or ethnicity) are not available

INTRODUCTION

Bowel screening using a faecal occult blood test (FOBt) enables identification of earlier stage cancers when treatment is more likely to be beneficial [1], ultimately leading to reduction in bowel cancer mortality [2]. The United Kingdom has well-established bowel screening programmes in each of its constituent countries. The Scottish Bowel Screening Centre (SBSC) sends a guaiac-based FOBt biennially to eligible patients aged 50-74 years [3]. The current uptake is 57.7%; with lower participation among the most deprived populations compared with the least deprived groups (45.5% vs 66.6% respectively). Uptake is higher for women (60.6%) compared to men (54.7%) [4].

Both barriers to uptake and effective strategies to increase participation in bowel screening are described in the literature. Lack of awareness of bowel cancer [5] or of screening [6], concerns about unpleasantness and embarrassment [7, 8], fear of the outcome [5, 9], fatalism [10] and perception of risk [11, 12] are commonly identified barriers. Nevertheless, there is good evidence that reminders targeting patients [13, 14] and physicians [5], having one-on-one interactions/education with general practitioners (GPs) and/or nurses [6, 15] and GP endorsements [6, 16] have a positive impact on screening uptake.

Primary care has an important and increasing role in cancer prevention and cancer screening [17]. In the UK alone, a number of interventions involving primary care have been recently developed [1, 13, 16, 18, 19]. In Scotland, a government programme aiming to improve cancer survival (the Detect Cancer Early Programme) [20] provided a financial incentive for practices meeting defined bowel screening targets [21, 22].

For peer review only - http://bmjopen.bmj.com/site/about/guidelines.xhtml

In this context, we aimed to test the feasibility and acceptability of an opportunistic intervention in general practice patient consultations, examining whether a brief conversation was a viable way to engage with non-responders and increase bowel screening participation. The study was undertaken in the Lothian region of Scotland which has slightly lower bowel screening uptake (57.2%) than the national average, and shows similar variation based on sex and socio-economic status [4].

MATERIALS AND METHODS

Recruitment of Practices and Patients

Practice recruitment

NHS Lothian provided the research team with a list of 112 practices in this region. The list had information on practice code, % screening uptake in 2013, practice list size, number of patients aged 50-75, number of average monthly non-responders, mean Scottish Index of Multiple Deprivation (SIMD) decile [23] (for those aged 50-75), and whether or not practices took part in the bowel Scottish Quality and Outcomes Framework (SQoF) [21]. Eleven general practices were purposively selected for a first wave of recruitment. We oversampled among the most deprived practices with lower uptake (as it was perceived that these practices could benefit the most from the intervention), while also taking into account the other factors listed above (as these would impact on how many patients could potentially be approached during the study period). Practices were invited to take part in the study via a personalised email sent by the study's principal investigator.

A visit was scheduled at the practices that were interested in taking part in the study. A brief information session was delivered, giving background information on colorectal cancer and screening, known barriers and facilitators to screening uptake and a thorough description of the study. Practices also received a folder containing the intervention materials, study information sheet, ethical approvals, background information on bowel cancer and bowel screening and a consent form.

Estimated size of study population

A preliminary calculation estimated that each recruited general practice would have the opportunity to engage with up to 182 potentially eligible patients during the study period (Supplementary File 1). These figures are

For peer review only - http://bmjopen.bmj.com/site/about/guidelines.xhtml

rough estimates as the study findings will guide calculations of a powered sample size for a larger study. We aimed to recruit up to six general practices for testing the intervention's feasibility and acceptability.

Patient inclusion and exclusion criteria

The target population were men and women aged 50-74 registered in a participating Lothian practice who received an invitation to screening and did not return a completed kit with a definitive screening result within 90 days (i.e. the official SBSC's definition of a non-responder). Patients were excluded from the study when they lacked mental capacity as defined by the Mental Capacity Act 2005 [24] and at professionals' discretion where patients were regarded as too ill (e.g. undergoing cancer treatment or in receipt of palliative care) or distressed to take part.

Identifying bowel screening non-responders

NHS Lothian and the SBSC routinely provide Scottish general practices with a list of non-responders. The research team worked alongside each participating practice to create a customised plan to ensure they could efficiently flag non-responders in their computer systems if they did not already have a system in place.

Intervention

The intervention comprised a brief conversation about bowel screening with non-responders. During a consultation with an eligible patient, the primary care professional (PCP) (a GP, practice nurse or health care assistant) raised the topic of non-participation using neutral statements and discussed any patient concerns. A leaflet with further information and an opportunity to request a bowel screening kit (via email, phone or tear off slip with FREEPOST) was offered. The intervention was designed to last 3-5 minutes. As part of the intervention, patients could also choose whether or not to develop a written plan of how to complete and return the kit (an implementation intention) [25]. In addition to the information leaflet and FREEPOST envelope, the intervention was supported by: an A5 set of 3-4 suggested questions/topic for discussion; an intervention flowchart and guidance sheet for PCPs (Supplementary File 1).

In developing the intervention content we drew on our previous work on strategies promoting uptake of FOBt screening [26], and available literature on factors associated with uptake and barriers to screening. We also drew on psychological models, principally on Implementation Intentions [25], the Health Behaviour Framework

For peer review only - http://bmjopen.bmj.com/site/about/guidelines.xhtml

[27], and were guided by principles of motivational interviewing [28] and informed choice [29]. The process of developing the brief intervention will be reported elsewhere (manuscript under peer review). We sought approaches (and wording in our materials) which were not coercive, but invited participants to consider the offer of screening after balancing potential benefits and harms. Materials conformed to the Scottish Bowel Screening Programme [30] and the NHS Cancer Screening Programme [31] resources and guidance.

Data Collected for Evaluation

Delivery of the intervention

PCPs logged details of each intervention on a proforma (contents in Supplementary File 1). Researchers regularly visited practices to collect these and to distribute materials as required, recording all communication/events in an intervention log. The intervention was planned to run for 3-4 months in each practice, depending on availability.

Requests for screening kits

Requests for new kits were made to the SBSC, which logged both the requests using the tear-off slip, and the returned kits. It was not possible to identify email or telephone requests relating to this project due to the high volume received through these means on a daily basis. For the purposes of comparison, the SBSC also provided data on total number of requests for a replacement kit made by each of the recruited practices during the intervention period (plus one extra month to allow time for requests to come in), and for equivalent periods at six months, one year and two years before the intervention.

Questionnaire and Interview Data

The feasibility parameters and mechanisms to be investigated are available in Supplementary File 1. Brief end of study questionnaires were developed for primary care and bowel screening staff. The questionnaires comprised closed and open-ended questions and focused on intervention acceptability and potential impact on workload. Semi-structured interviews were carried out with members of the practice team (aiming to interview the practice manager and at least one GP or practice nurse). Interviews sought to ascertain views on the running of the brief intervention, its acceptability, and its overall feasibility as part of routine primary care.

Data analysis

For peer review only - http://bmjopen.bmj.com/site/about/guidelines.xhtml

Templates were created using SPSS 19 for Windows [32] to collect data on the practice proforma and the end of study questionnaires. Quantitative data from the SBSC, proformas and end of study questionnaires were analysed using descriptive statistics (summaries, frequencies and cross-tabulations). As we had a purposeful sample of practices and a non-random sample of patients, no inferential statistics were calculated [33].

End of study interviews were digitally recorded and transcribed verbatim. Qualitative data were analysed using thematic analysis informed by a framework approach including techniques of familiarisation, coding, indexing, charting, mapping and interpretation [34, 35] assisted by QSR NVivo 7 software [36]. This approach was considered appropriate due to the pre-existing feasibility parameters being tested. Identified themes were considered in the context of these parameters and interpreted according to existing theory and research. Scrutiny both within and across transcripts ensured that the analysis encompassed all perspectives and used the whole dataset. All transcripts were read by two researchers (DC and NC) and 50% were subject to triple initial coding (DC, NC and CC). The initial coding framework was developed by DC and was reiterated following discussion, with any discrepancies explored and accounted for. Content analysis [37] was used to summarise, categorise and interpret text entries made by practice staff on proformas to record reasons for consultation.

Ethical approval and consent

The study was approved by the South East Scotland Research Ethics Committee 01 (reference 14/SS/1067) and the NHS Lothian's Research and Development Office (Project Number 2014/0366). The Scottish Bowel Screening Governance Reference Group also approved the study. Written informed consent was obtained via the practice manager or GP partner and separate consent was obtained for end of study interviews. Practices were reimbursed for their participation.

RESULTS

Practice recruitment

Six out of 11 invited practices consented to participate in the study; one practice subsequently withdrew due to resource issues (Supplementary file 2). The five remaining practices varied in size, bowel screening uptake and deprivation levels (Table 1). All but one practice were signed up to the SQoF.

For peer review only - http://bmjopen.bmj.com/site/about/guidelines.xhtml

For peer review only

Table 1 Characteristics of recruited practices

Recruited Practices	Location	Uptake % (2013)	Pop 50-75	Mean SIMD decile (50-75 year olds)*	Average monthly non-responders	Practice list size	Signed up to SQoF	Start date	End date
Practice A	Edinburgh	<45%	1,413	2.6	41	6,888	No	04/03/15	05/07/15
Practice B	Edinburgh	45-50%	2,654	3.6	61	10,440	Yes	14/04/15	15/08/15
Practice C	East Lothian	50-55%	2,515	4.5	56	8,693	Yes	22/04/15	03/09/15
Practice D	Edinburgh	50-55%	1,241	6.2	29	5,326	Yes	20/04/15	24/08/15
Practice E	Midlothian	55-60%	1,668	5.5	30	5,201	Yes	05/03/15	08/07/15

Abbreviations: Pop: population; SIMD: Scottish Index of Multiple Deprivation; SQoF; Scottish Quality and Outcomes Framework.

*The Scottish Index of Multiple Deprivation (SIMD) is a measure of multiple deprivation which combines different domains related to employment, income, health, education, skills and training, geographic access to services, crime and housing [23]. The lower the decile number, the higher the deprivation levels.

Setting up the intervention

Although all practices were routinely provided with an electronic list of non-responders, there was variation in the methods in place to identify non-responders during consultations. In two practices, the researchers coded non-responders into practices' computer systems, also helping to insert screen 'pop-up' reminders in one of these cases. The remaining three practices already had systems in place.

One practice developed a digital proforma in their GP system instead of using the paper-based one provided by the research team. Planned monthly visits were not always required and were adapted to suit practice needs.

Intervention delivery, acceptance and impact on screening uptake

Overall, 258 patients were approached between March and September 2015. Men were approached slightly more often than women (53.1% vs 46.9%) and most patients were among the younger eligible age groups for screening (median 58.00, interquartile range (IQR) 53.00-65.00) (Table 2). No information on patient ethnicity was available. The median duration of the intervention was 2.00 minutes (IQR 1.25-5.00).

For peer review only

Table 2. Patient and staff characteristics

Overall data	Practice A n(%)	Practice B n(%)	Practice C n(%)	Practice D n(%)	Practice E n(%)	Total n(%)
**Patient sex**						
Men	43 (60.6)	25 (45.5)	8 (28.6)	43 (61.4)	18 (52.9)	137 (53.1)
Women	28 (39.4)	30 (54.5)	20 (71.4)	27 (38.6)	16 (47.1)	121 (46.9)
Total	71 (100.0)	55 (100.0)	28 (100.0)	70 (100.0)	34 (100.0)	258 (100.0)
**Patient age**[a]						
Median	55.50	55.50	58.50	63.00	59.00	58.00
(IQR)	(53.00-64.00)	(51.75-62.25)	(52.25-67.75)	(56.25-69.00)	(53.75-63.00)	(53.00-65.00)
50-54	25 (35.7)	24 (44.4)	10 (35.7)	13 (19.1)	11 (32.4)	83 (32.7)
55-59	22 (31.4)	9 (16.7)	5 (17.9)	14 (20.6)	7 (20.6)	57 (22.4)
60-64	8 (11.4)	10 (18.5)	4 (14.3)	11 (16.2)	11 (32.4)	44 (17.3)
65-69	6 (8.6)	6 (11.1)	4 (14.3)	15 (22.1)	5 (14.7)	36 (14.2)
70-74	9 (12.9)	4 (7.4)	3 (10.7)	10 (14.7)	0 (0.0)	26 (10.2)
75-79	0 (0.0)	1 (1.9)	2 (7.1)	5 (7.4)	0 (0.0)	8 (3.1)
Total	70 (100.0)	54 (100.0)	28 (100.0)	68 (100.0)	34 (100.0)	254 (100.0)
**Interventions by primary care role**						
GP	33 (46.5)	31 (56.4)	21 (75.0)	50 (71.4)	31 (91.2)	166 (64.3)
PN	38 (53.5)	11 (20.0)	7 (25.0)	20 (28.6)	3 (8.8)	79 (30.6)
HCA	0 (0.0)	13 (23.6)	0 (0.0)	0 (0.0)	0 (0.0)	13 (5.0)
Total	71 (100.0)	55 (100.0)	28 (100.0)	70 (100.0)	34 (100.0)	258 (100.0)

Abbreviations: GP: General Practitioner; PN: Practice Nurse; HCA: Health Care Assistant.
[a]Eight patients aged 75 or older were included as their last invitation to screening happened before their 75th birthday (hence meeting eligibility criteria).
Missing data: There were no missing data for patient sex and staff carrying out the intervention. There were 4 missing cases for patient age. Percentages may not add up to 100 due to rounding.

The majority of the interventions were carried out by GPs (64.3%), followed by practice nurses (30.6%) and health care assistants (5.0%). Patients receiving the intervention consulted for a variety of reasons (Supplementary File 3). The main reasons were reviews of existing conditions (22.0%), consultations due to musculoskeletal symptoms/conditions (13.2%), to carry out tests or obtain test results (11.6%), or due to respiratory or ear, nose and throat symptoms/conditions (8.8%).

The majority of patients who were offered the intervention accepted it (i.e. engaged in conversation) (87.0%), with variations across practices (Table 3). The leaflet was given to 74.2% of patients. Almost a quarter of the 258 patients approached (n=60) requested a replacement kit using a reply slip. Over a third of these patients (n=22) also returned a completed kit.

For peer review only

Table 3. Intervention acceptability, requested and returned kits

Overall data		Practice A n(%)	Practice B n(%)	Practice C n(%)	Practice D n(%)	Practice E n(%)	Total n(%)
Duration of intervention (minutes)[a]	Median (IQR)	3.50 (2.00-5.00)	2.00 (2.00-4.50)	2.00 (1.00-5.00)	2.00 (2.00-3.25)	1.00 (1.00-2.00)	2.00 (1.25-5.00)
Acceptance of intervention	Accepted (yes)	56 (78.9)	46 (85.2)	24 (96.0)	61 (88.4)	33 (97.1)	220 (87.0)
	Leaflet given (yes)	40 (57.1)	44 (81.5)	24 (85.7)	48 (68.6)	34 (100.0)	190 (74.2)
	Leaflet completed in practice (yes)	10 (14.3)	9 (16.7)	0 (0.0)	19 (27.9)	0 (0.0)	38 (17.3)
Requested kits using a slip	N requested kits/total interventions (% requesting a kit amongst total interventions)	16/71 (22.5)	7/55 (12.7)	6/28 (21.4)	25/70 (35.7)	6/34 (17.7)	60/258 (23.3)
Returned kits	N completed kits returned/total requested kits (% completing kits amongst total requests)	1/16 (6.3)	4/7 (57.1)	5/6 (83.3)	8/25 (32.0)	4/6[c] (66.7)	22/60 (36.7)
Test results	Negative	1	3	4	8	4	20
	Positive	0	0	1	0	0	1
	Pending[b]	0	1	0	0	0	1
Non-responders approached who became a responder to screening	N completed kits/N approached non-responders (% approached who became a responder)	1/71 (1.4)	4/55 (7.2)	5/28 (17.9)	8/70 (11.4)	4/34 (11.8)	22/258 (8.5)
Non-responders accepting the intervention who became a responder to screening	N completed kits/N accepting intervention (% accepting intervention who became a responder)	1/56 (1.8)	4/46 (8.7)	5/24 (20.8)	8/61 (13.1)	4/33 (12.1)	22/220 (10.0)

Abbreviations: IQR: Interquartile Range.
Missing data: there were 10 missing cases for duration of intervention, 5 for whether intervention was accepted, 2 for whether leaflet was given and 4 for whether it was completed in the practice. The same denominator (i.e. the total number of interventions carried out) applies for each question about acceptance of the intervention (i.e. intervention accepted, leaflet given and leaflet completed in practice) due to issues observed in data entry. Overall 4 leaflets were given although intervention was ticked as not accepted and 12 leaflets were completed in practice although they were ticked as not given to the patient.
[a]Over 90% of the interventions (n=225) lasted up to 5 minutes.
[b]A weak positive result (not shown) indicates that further tests are needed; in one case results for further tests were not yet available so results are shown as pending. In another case a weak positive became a positive result after further tests.
[c]One patient from Practice E requested a kit but was not sent one as s/he was only due for a new test in 2016. The National Bowel Screening System (BoSS) does not allow for sending additional kits for patients who are not due for another test; this helps to avoid over-screening.

For peer review only - http://bmjopen.bmj.com/site/about/guidelines.xhtml

Younger participants were more likely to accept the intervention (median age 58.00; IQR 53.00-64.75 for those accepting the intervention vs. 64.00; IQR 57.00-71.50 for those not accepting it). Men refused the intervention more often than women (the former represented 66.7% of all refusals). Over half (57.6%) of refused interventions were carried out by a practice nurse (Supplementary File 3).

Descriptive data from the Bowel Screening Centre on requested kits (Supplementary File 4) show that there was an increase in the number of requested kits across all practices during the intervention period (the highest increase in practice D and the lowest in Practice E) compared to two years, one year and six months prior to the intervention.

End of study evaluation: qualitative interviews

Eleven individual and one group in-depth qualitative interviews were conducted with a total of 14 primary care staff (four GPs, four practice nurses, five practice managers and one health care assistant). Thirteen interviews were face to face and one was via telephone. Findings from the qualitative interviews identified four main domains: the primary care and general *health care context*; the *processes* involved *in delivering the intervention*; *patient-related acceptability*; and *primary care professional acceptability* (Table 4).

For peer review only

For peer review only - http://bmjopen.bmj.com/site/about/guidelines.xhtml

Table 4. End of study interview quotes to support findings

1.Health care context

Existing practices

- We remind patients about cervical screening, so it's on a, sort of, slightly similar vein." Practice Nurse, Practice D
- "I think similar to our alcohol brief interventions and I think it enables us to initiate conversation…about an important subject which we might not otherwise do." GP, Practice D

Pressurised work environment

- "Because of the state of the practice at the minute when we've got doctors leaving - retiring and resigning – it's just put an added burden on existing people to do that." Practice Manager, Practice C
- "Just part of a greater workload issue. We're struggling to provide our contracted services, so I'm not going to commit to take on anything now unless it's properly resourced." GP, Practice E

Acknowledging barriers to screening

- "But it's always practicalities. That's why people don't want to do it." Practice Nurse, Practice D
- It's interesting because the research does show there is a kind of gradient there and that in some minority groups the uptake is not as high." GP, Practice D

Knowledge of and attitude to bowel screening

- It's definitely an extra to add in to the patients but I'm a real proponent of preventive health care and I think these things are worthwhile." GP, Practice D
- "To be honest, the practice population that we have have far bigger problems than whether they did their bowel screening or not, so in the real world it's possibly not one of the things we would include." Practice Nurse, Practice A

2. Processes in implementing the intervention

Providing information on the intervention

- "Actually it was very helpful, because I hadn't understood what the patients were being asked to do." GP, Practice E
- "They all thought it was a very worthwhile thing to sign up to." Practice Manager, Practice C

Appropriate timing and scenarios for the intervention

- "Probably if you have had any consultations that has presented with six problems and the last thing you need is to get into something else." GP, Practice B
- "I think when you leave it to the end you have not encroached on the patient's time." Practice Nurse, Practice B

Use of intervention supporting materials

- "You maybe look at it once or twice and see what's the kind of chat and then you probably don't dig it out every time, it's so opportunistic. GP, Practice A

Minimising paperwork, adaptations and integrating the intervention into existing IT

- "Obviously if we had a reminder for everything […] then we wouldn't be able to see the screen for reminders. So it's okay in the short term but in the long term it's a bit more difficult." Practice Manager, Practice A
- "We've got a computer, so it tells you that you need a bowel intervention, so why (not) record the data that you wanted on the same system?" GP, Practice E

Time limitations

- "I was aware that we were missing lots of people as the GPs simply didn't have time." Practice Manager, Practice C
- "In GP land when you've got ten minutes, ten minutes, ten minutes, then every little five minutes counts." GP, Practice A

Constraints in implementing the intervention

- "It all boils down to sometimes some men don't want to discuss it. […] I've found that sometimes a barrier, especially with older men." Practice Nurse, Practice D
- "We have a high Asian population and they are not keen to talk about poo or the practicalities of keeping their kit beside the toilet." GP, Practice D

Translating intention into action

- "When you actually spoke about it they thought it was a good idea, "I'll do it", but whether they do it or not, don't know." Practice Nurse, Practice B

For peer review only

For peer review only - http://bmjopen.bmj.com/site/about/guidelines.xhtml

3. Patient factors and acceptability

Patient receptivity

- “I had no bad experiences at all. People were happy to talk about it.” Practice Nurse, Practice E
- “I was surprised at how receptive the patients were to it.” GP, Practice B

Patient awareness and support for bowel screening

- “They knew pretty much what was involved.” Practice Nurse, Practice E
- My own finding was as soon as you mentioned it to patients and brought up screening, the majority of them were keen to go ahead and do it.” GP, Practice B

Patient priorities and motivation to participate in bowel screening

- “The patient would say, well that’s the least of my concerns and I’ll tell you why…” GP, Practice A
- “Just sort of, inertia and couldn’t be bothered, not a priority.” GP, Practice E

4. Primary care professional factors and acceptability

Acceptability to professionals

- “It isn’t an onerous thing to do and what they have to do is fairly straightforward.” Practice Manager, Practice E
- “It’s just an extension of normal dialogues really.[…] I think it’s entirely appropriate and problem, well almost problem free.[…] It was quick and simple to do and if the feedback turns out to that it’s effective, then I think it would be an appropriate thing to implement in practice.” GP, Practice E

Professional interest, variable support and priority

- “I think there was a bit of a mixed response. I think generally GPs when they’re asked to do something over and above are just like whoa, we’re totally overwhelmed.” GP, Practice A
- “I mean they are so busy here. They are always running late […] so whether or not it just hasn't been a great priority for them.” Practice Nurse, Practice E

Motivation to adopt the intervention

- “Bowel screening is effective and we didn’t have to sell that concept to them. […] I think if they think it’s a good thing they’re more likely to advocate it.” Practice Manager, Practice B
- “Sometimes it felt like quite a positive thing to do because it is about health promotion and disease prevention and that very much chimes with our ethos.” GP, Practice A

The intervention as part of a broader preventive health agenda

- “I think we have to be trying to educate people to look after themselves instead of fixing things after they’re broken.” Practice Nurse, Practice A
- “Giving them a message of empowering them to take control of their destiny, which is something I think that is really lacking in a population like ours.” GP, Practice A

The perceived professional role in educating patients and raising awareness

- “It was a good opportunity to bring it to the forefront of their consciousness […] to kind of put some medical opinion behind it and say, “this is the reason we are doing it”, you know. It does reduce your chances of having a serious bowel cancer if we catch it early.” GP, Practice D
- It was just talking round the practicalities. […] “Oh, I just didn’t know how to do it” […] so you’d try to talk through it a little bit with them.” Practice Nurse, Practice D

Potential for differing roles and involvement

- “I’m a more junior practice nurse, people aren’t coming to me with loads of things, […] so maybe I have more time to look at it.” Practice Nurse, Practice E
- “I do think that the nurses will integrate it more than the GPs will. […] I think GPs deal with the more acute problems, whereas health checks you’ve maybe got a bit more time and people are more relaxed and they are expecting you to ask that.” Practice Manager, Practice A

For peer review only - http://bmjopen.bmj.com/site/about/guidelines.xhtml

Health care context

PCPs reported that certain organisational aspects of primary care services impacted on the implementation of the brief intervention. Existing health promotion interventions already placed demands on practices. PCPs emphasised the highly pressured primary care work environment with a cumulative impact on their ability to commit to new projects.

PCPs also highlighted important barriers to bowel screening participation such as embarrassment and practical issues, in addition to the influence of gender and ethnicity. There was variation by practitioner and also across practices, reflecting PCP's knowledge and belief in screening and also particular patient populations (with reported constraints such as illiteracy and high levels of deprivation).

Processes in delivering the intervention

PCPs commented on the usefulness of the background information on bowel cancer and screening to increase their understanding and belief in screening and the intervention, fostering a sense of commitment. In relation to delivering the intervention itself, staff commented on appropriate types of consultations to raise the topic of screening, appropriate timing within the consultation, frequency of use of the intervention materials, adaptations made, and issues around logging interventions. Staff also referred to use of computer systems to highlight non-responders (also serving as a reminder) and to log and monitor interventions, as well as the need to minimise paperwork and integrate any future interventions into existing computer systems. Constraints raised in ability to deliver the intervention related to difficulty in interacting on the topic of bowel screening with certain groups (e.g. males and minority ethnic groups), but mainly to limited time in an already pressured environment.

Patient factors and acceptability

PCPs reported that patients were positive or neutral but rarely negative when engaging on the topic of screening. They felt that patients were overall receptive to the intervention and discussing bowel screening. In a number of cases, patients had knowledge of bowel cancer and bowel screening and were aware of the benefits of taking part, but there was a large degree of perceived 'inertia', where bowel screening did not appear to be a priority.

For peer review only - http://bmjopen.bmj.com/site/about/guidelines.xhtml

Primary care professional factors and acceptability

The PCPs reported the importance of increasing bowel screening participation and found the process to be acceptable, straightforward and easy to administer as part of routine consultations. However, PCPs reported variation depending on factors such as special interests, personal experience, perceived priorities for the patient population, and forced priorities as a result of limited time and work pressures. Professionals also acknowledged the influence of their attitude towards bowel screening on their approach to the intervention. Those who were motivated drew on the importance of screening and their belief in a holistic approach to health care whereas for others bowel screening was not the highest priority in order to improve patient care.

Interviewees were cognisant of their role in the intervention process in educating patients about bowel cancer and screening and raising awareness of the benefits of participating, and how sometimes this alone was enough to prompt patients to take part. However, they felt they lacked control once the patient had left the consulting room over whether or not they ultimately returned a FOBt kit. There was also discussion of the most appropriate member of the practice team to take the intervention forward, whether this be related to time available, role (GP, PN or HCA), practice load or special interest.

Practice staff reported it was feasible to roll out the intervention and made suggestions for certain adjustments to make it more effective, such as handing out kits directly to patients; streamlining any written materials and making them electronic; integrating any data recording into existing computer systems; and considering funding and set time periods dedicated to bowel screening that complement other initiatives.

End of study evaluation: questionnaires

Nineteen PCPs returned a completed questionnaire (response rate 38.8%). Thirteen were GPs, five were practice nurses and one was a practice manager. This group carried out over half (51.2%) of all interventions (n=132). As reflected in the qualitative interviews, all but one GP (no recorded interventions) stated that most patients were receptive to the intervention; that it could be easily incorporated into practice; and they would theoretically be willing to take part in the study again. Nonetheless, despite positive feedback, ten professionals highlighted lack of time as a potential or actual barrier.

For peer review only - http://bmjopen.bmj.com/site/about/guidelines.xhtml

Four bowel screening staff (out of seven; three screening officers and the screening supervisor) whom had been involved in the intervention returned a completed end of study questionnaire. They all stated that the intervention could be easily incorporated into their workload. However, opinion on the potential impact on workload was uncertain. Three respondents stated that it was difficult identifying calls from patients in intervention practices among over 300 daily calls. However, all four reported that it was suitable for testing in a larger study in its present form.

DISCUSSION

Summary

Results indicate that the intervention was feasible and acceptable to PCPs and patients (as reported by professionals). Of those reached, a small but important minority became responders, a likely underestimate as email and telephone requests were not recorded. It is also possible that some patients may have completed their original kit at home and returned it. The majority of patients approached were willing to discuss the subject of bowel screening. Some patients may have made an informed choice not to participate in screening (indeed informed choice guided the intervention design), although this was not documented in this study.

Qualitative and questionnaire data indicate that the intervention was straightforward and easy to implement and reflected similar ongoing health promotion initiatives, and was thus an effective way to communicate with patients about bowel screening. Overall, PCPs were willing and felt comfortable delivering the intervention in different scenarios, suggesting suitability for most primary care consultations. Practices varied in the number of patients approached and reasons for this variability were widely described in the interviews. Inappropriate or challenging scenarios reported included those involving patients with complex health and social care needs, poor literacy, English as a second language, or sensitivities related to ethnicity and culture. Evidence on appropriate scenarios can help inform future interventions on how to approach these hard to reach groups. Furthermore, these findings have implications for the flexible design of the intervention at a larger scale so it meets the needs of individual practices and different patient groups.

PCPs stated that materials were helpful to promote the intervention, draw attention to it and reinforce messages post-consultation. Nonetheless, not all practices had systems in place to identify non responders,

For peer review only - http://bmjopen.bmj.com/site/about/guidelines.xhtml

or wished to use reminders long-term; both issues can influence the success of any future implementation. Finally, feedback from the SBSC regarding the intervention was also positive, but future implementation would need to take into account the difficulty recoding telephone and email requests and explore all potential mechanisms for requests to be made.

Study strengths and limitations

This was an evidence-based intervention informed by current data on non-participation and psychological theory, grounded in the pragmatic reality of the primary care workload. A good relationship with practices was developed. The study produced a clear audit trail and the duplicate coding of qualitative data helped ensure consistency, rigour and transparency. Nevertheless, this was a small feasibility study which requires further evaluation in larger patient populations. The study also targeted patients who consult in primary care; those who do not consult may present different challenges regarding participation. As we had to adapt to a dynamic, time-pressured primary care environment, it is unknown how many eligible non-responders consulted (and how many of these were approached). Some elements of the intervention were adapted by practices, this is expected in a complex intervention [38]. In fact, an intervention that can be adapted to local circumstances without loss of its essence is a strength that facilitates practical implementation. Finally, the context of governmental campaigns in Scotland promoting screening participation [39] and the SQoF rewards may have influenced practice decisions to participate in our study. It also made it harder to separate the impact of the campaigns and the intervention on patients' behavioural response.

Comparison with other studies

Brief interventions in primary care are well established and successful in influencing behaviours such as alcohol consumption [40],tobacco smoking [41], and weight management [42]. Our research also contributes to a body of recent UK studies examining primary care-based interventions to influence screening behaviour and demonstrating their effectiveness in improving bowel screening uptake [13, 18, 19], offering further evidence on the benefits of such interventions. Our results show that intervention acceptance varied across practices with the two most deprived practices having the lowest proportion of acceptance and the lowest number of kits requested and returned. This finding suggests that GP endorsement alone is not sufficient to change patient bowel screening behaviour among the most deprived groups, as reflected in a recent study [16].

For peer review only - http://bmjopen.bmj.com/site/about/guidelines.xhtml

PCPs reported lack of control after the patient leaves the consulting room - indeed only over a third of those requesting a kit actually returned it - and a gap between intention and action, a phenomenon well described in the literature [43, 44]. The implementation intention plan aimed to help deal with this limitation but was seldom used by PCPs and other studies have shown mixed effectiveness [45-49].

Implications for practice and further research

A higher proportion of people seen by a nurse did not accept the intervention. Disease monitoring was a common reason for seeing a nurse, reflecting official data on consultation patterns in Scotland [50]. Interviews show that both GPs and PNs saw their role as important, and some suggestion that GPs placed greater emphasis on educating and persuading patients. HCAs also reported being in a good position to deliver interventions, though the numbers in this study were small. There is scope to explore further the potential differing roles for members of the primary care team in this context.

The primary care context was described as a highly pressured environment comprising complex patients' needs, limited financial and human resources, increasing patients but diminishing staff, and the need to incentivise health promotion. When asked about the likelihood of continuing on with the intervention, it was clear that despite perceiving it as useful and supporting its underlying ethos, other pressing issues would be prioritised. These challenges constrain the ability to deliver and sustain the intervention, irrespective of motivation, willingness and recognised importance. However, the flexibility of the intervention meant that it could be adapted to suit individual practices and demonstrated an impact on bowel screening participation despite the outlined constraints.

CONCLUSIONS

We tested a primary care-based intervention to increase uptake in non-responders to FOBt screening, and found it to be feasible and acceptable in Scottish primary practices, despite recognised organisational and system constraints that would need to be considered for the intervention to be more widely implemented. Further testing in a randomised controlled trial would give robust evidence of the effectiveness of the brief intervention in increasing informed screening participation. The intervention can be useful as one tool to complement other efforts to engage with non-responders and reflects the broader aims from the Scottish

For peer review only - http://bmjopen.bmj.com/site/about/guidelines.xhtml

For peer review only

government to raise awareness and normalise bowel screening. Our study adds to evidence that primary care can play a key role in promoting bowel screening uptake.

AUTHORS' CONTRIBUTIONS

All authors designed the study. CC, DW, DC and NC were involved in recruitment, data collection and data analysis. All authors contributed to the drafting of the manuscript and the approval of the final version.

FUNDING STATEMENT

This work was supported by CRUK [grant reference number C12357/A13965] through the National Awareness and Early Diagnosis Initiative (NAEDI-2). The funder had no role in the design, collection, analysis, and interpretation of data; in the writing of the manuscript; and in the decision to submit the manuscript for publication.

CONFLICT OF INTEREST STATEMENT

The authors report no conflicts of interest.

DATA SHARING STATEMENT

Some of the unpublished data are available from the authors (such as intervention materials and questionnaires). The corresponding author (natalia.calanzani@ed.ac.uk) can be contacted by anyone interested in accessing these. Data from patients, primary care practices and the Scottish Bowel Screening Centre cannot be accessed by anyone who is not part of the research team due to ethical and confidentiality concerns.

ACKNOWLEDGEMENTS

Bowel screening data were obtained from the Scottish Bowel Screening Centre in Dundee; we thank all staff for their assistance and for completing the end of study questionnaires. We acknowledge the support and advice from NHS Lothian, which provided the practice lists used for recruiting practices and the help from NHS Scotland which provided the leaflets in Polish. We thank Dr Robert Scully for his help with medical acronyms/terms in the intervention proformas. We are very grateful to each GP, practice nurse, health care assistant and practice manager who agreed to take part in the study, gave up their time to be interviewed and completed the end of study questionnaires.

For peer review only

REFERENCES

[1] Damery S, Smith S, Clements A, et al. Evaluating the effectiveness of GP endorsement on increasing participation in the NHS Bowel Cancer Screening Programme in England: study protocol for a randomized controlled trial. *Trials* 2012;13:18.
[2] Hewitson P, Glasziou Paul P, et al. Screening for colorectal cancer using the faecal occult blood test, Hemoccult. Cochrane Database Syst Rev: John Wiley & Sons, Ltd, 2007.
[3] Bowel Cancer UK. Screening in the Nations. 2015.
[4] Scottish Bowel Screening Programme. Scottish Bowel Screening Programme. Key Performance Indicators Report: May 2015 data submission. Invitations between 1st November 2012 and 31st October 2014. https://www.isdscotland.org/Health-Topics/Cancer/Publications/2015-08-04/2015-08-04-Bowel-Screening-KPI-Full-Report.xlsx, 2015
[5] European Commission. European guidelines for quality assurance in colorectal cancer screening and diagnosis. Luxembourg: Publications Office of the European Union, 2010.
[6] Holden DJ, Harris R, Porterfield DS, et al. Enhancing the use and quality of colorectal cancer screening. *Evid Rep Technol Assess* 2010:1-195.
[7] Jepson R, Clegg A, Forbes C, et al. The determinants of screening uptake and interventions for increasing uptake: a systematic review. *Health Technol Assess* 2000;4:i-vii,1-133.
[8] Palmer CK, Thomas MC, von Wagner C, et al. Reasons for non-uptake and subsequent participation in the NHS Bowel Cancer Screening Programme: a qualitative study. *Br J Cancer* 2014;110:1705-11.
[9] Chapple A, Ziebland S, Hewitson P, et al. What affects the uptake of screening for bowel cancer using a faecal occult blood test (FOBt): a qualitative study. *Soc Sci Med* 2008;66:2425-35.
[10] Power E, Miles A, von Wagner C, et al. Uptake of colorectal cancer screening: system, provider and individual factors and strategies to improve participation. *Future Oncol* 2009;5:1371-88.
[11] Malila N, Senore C, Armaroli P. European guidelines for quality assurance in colorectal cancer screening and diagnosis. First Edition--Organisation. *Endoscopy* 2012;44 Suppl 3:Se31-48.
[12] Powe BD, Faulkenberry R, Harmond L. A review of intervention studies that seek to increase colorectal cancer screening among African-Americans. *Am J Health Promot* 2010;25:92-9.
[13] Wardle J, von Wagner C, Kralj-Hans I, et al. Effects of evidence-based strategies to reduce the socioeconomic gradient of uptake in the English NHS Bowel Cancer Screening Programme (ASCEND): four cluster-randomised controlled trials. *Lancet* 2015;387:751-59.
[14] Brouwers MC, De Vito C, Bahirathan L, et al. Effective interventions to facilitate the uptake of breast, cervical and colorectal cancer screening: an implementation guideline. *Implement Sci* 2011;6:112.
[15] Sabatino SA, Lawrence B, Elder R, et al. Effectiveness of interventions to increase screening for breast, cervical, and colorectal cancers: nine updated systematic reviews for the guide to community preventive services. *Am J Prev Med* 2012;43:97-118.
[16] Raine R, Duffy SW, Wardle J, et al. W. Impact of general practice endorsement on the social gradient in uptake in bowel cancer screening. *Br J Cancer* 2016;114:321-6.
[17] Rubin G, Berendsen A, Crawford SM, et al. The expanding role of primary care in cancer control. *Lancet Oncol* 2015;16:1231-72.
[18] Hewitson P, Ward AM, Heneghan C, et al. D. Primary care endorsement letter and a patient leaflet to improve participation in colorectal cancer screening: results of a factorial randomised trial. *Br J Cancer* 2011;105:475-80.
[19] Shankleman J, Massat NJ, Khagram L, et al. Evaluation of a service intervention to improve awareness and uptake of bowel cancer screening in ethnically-diverse areas. *Br J Cancer* 2014;111:1440-7.
[20] The Scottish Government. Detect Cancer Early. 2015. http://www.gov.scot/Topics/Health/Services/Cancer/Detect-Cancer-Early
[21] ISD Scotland, National Services Scotland. Quality and Outcomes Framework. Prevalence, achievement, payment and exceptions data for Scotland, 2013/2014. https://isdscotland.scot.nhs.uk/Health-Topics/General-Practice/Publications/2014-09-30/2014-09-30-QOF-Report.pdf?44355410338, 2014
[22] The Scottish Government. Detect Cancer Early - Bowel Screening Initiative. Health and Social Care Integration Directorate Primary Care Division, 2013.
[23] The Scottish Government. Scottish Index of Multiple Deprivation 2012. 2012. http://simd.scotland.gov.uk/publication-2012/.

For peer review only - http://bmjopen.bmj.com/site/about/guidelines.xhtml

[24] Department for Constitutional Affairs. Mental Capacity Act 2005 Code of Practice. London, 2007. https://www.gov.uk/government/uploads/system/uploads/attachment_data/file/497253/Mental-capacity-act-code-of-practice.pdf.
[25] Gollwitzer PM. Goal Achievement: The Role of Intentions. *Eur Rev Soc Psychol* 1993;4:141-85.
[26] Campbell C, Techer L, Crawford F, et al Primary Care based strategies to increase uptake of colorectal cancer screening. USA: Report to the National Cancer Institute, 2009.
[27] Bastani R, Glenn BA, Taylor VM, et al. Integrating theory into community interventions to reduce liver cancer disparities: The Health Behavior Framework. *Prev Med* 2010;50:63-7.
[28] Treasure J. Motivational interviewing. *Adv Psychiatr Treat* 2004;10:331-7.
[29] Jepson RG, Hewison J, Thompson A, et al. Patient perspectives on information and choice in cancer screening: a qualitative study in the UK. *Soc Sci Med* 2007;65:890-9.
[30] NHS Inform. Risks and benefits of bowel screening. 2013. https://www.nhsinform.scot/healthy-living/screening/bowel-screening/introduction-to-bowel-screening#benefits-and-risks
[31] NHS Cancer Screening Programmes. Consent to Cancer Screening. Cancer Screening Series No 4., 2008.
[32] IBM SPSS Statistics for Windows, Version 19.0. Armonk, NY: IBM Corp.: IBM Corp, 2010.
[33] Baker R, Brick MJ, Bates NA, et al. Report of the AAPOR Task Force on Non-Probability Sampling. 2013. https://www.aapor.org/AAPOR_Main/media/MainSiteFiles/NPS_TF_Report_Final_7_revised_FNL_6_22_13.pdf
[34] Gale N, Heath G, Cameron E, et al. Using the framework method for the analysis of qualitative data in multi-disciplinary health research. *BMC Med Res Methodol* 2013;13:117.
[35] Ritchie J, Spencer L. Qualitative data analysis for applied policy research. In: Bryman A, Burgess R, editors. Analyzing qualitative data. Abindgon: Routledge, 1994:173-94.
[36] NVivo qualitative data analysis Software. QSR International Pty Ltd, 2006.
[37] Krippendorf K. Content analysis: An introduction to its methodology. Beverly Hills, CA: Sage Publications, 1980.
[38] Medical Research Council. Developing and evaluating complex interventions: new guidance. http://www.mrc.ac.uk/documents/pdf/complex-interventions-guidance/, 2008.
[39] The Scottish Government. New bowel cancer home screening test. 2015. http://news.gov.scot/news/new-bowel-cancer-home-screening-test#downloads
[40] Kaner EF, Beyer F, Dickinson HO, et al. Effectiveness of brief alcohol interventions in primary care populations. *Cochrane Database Syst Rev* 2007:Cd004148.
[41] Bauld L, Bell K, McCullough L, et al. The effectiveness of NHS smoking cessation services: a systematic review. *J Public Health* 2010;32:71-82.
[42] Aveyard P, Lewis A, Tearne S, et al. Screening and brief intervention for obesity in primary care: a parallel, two-arm, randomised trial. *Lancet* 2016;388:2492–500.
[43] Sheeran P. Intention—Behavior Relations: A Conceptual and Empirical Review. *Eur Rev Soc Psychol* 2002;12:1-36.
[44] Power E, Van Jaarsveld CH, McCaffery K, et al. Understanding intentions and action in colorectal cancer screening. *Ann Behav Med* 2008;35:285-94.
[45] Sheeran P, Orbell S. Using implementation intentions to increase attendance for cervical cancer screening. *Health Psychol* 2000;19:283-9.
[46] Rutter D, Quine L, Steadman L, et al. Increasing Attendance at Breast Cancer Screening: Field Trial. Final Report to NHSBSP. University of Kent. Department of Psychology. Centre for Research in Health Behaviour, 2007.
[47] Greiner KA, Daley CM, Epp A, et al. Implementation Intentions and Colorectal Screening: A Randomized Trial in Safety-Net Clinics. *Am J Prev Med* 2014;47:703-14.
[48] Lo SH, Good A, Sheeran P, et al. Preformulated implementation intentions to promote colorectal cancer screening: a cluster-randomized trial. *Health Psychol* 2014; 33:998-1002.
[49] Walsh JC. Increasing Screening Uptake for a Cervical Smear Test: Predictors of Attendance and the use of Action Plans in Prior Non-Attenders. *The Irish Journal of Psychology* 2005;26:65-73.
[50] ISD Scotland. Practice Team Information (PTI) Annual Update (2012/13). http://www.isdscotland.org/Health-Topics/General-Practice/Publications/2013-10-29/2013-10-29-PTI-Report.pdf, 2013.

For peer review only

For peer review only - http://bmjopen.bmj.com/site/about/guidelines.xhtml

Supplementary file 1:

A. Estimating the size of the study population
B. Intervention supporting materials
C. Feasibility parameters

A. Estimating the size of the study population

According to the 2011 Census, there are 184,846 residents in Lothian aged 50-74[1] (the age range for eligibility to bowel cancer screening). In these age groups, approximately 86% of patients (estimated 158,968) consult at least once a year (aged 45 and over are included as data were not available separately)[2], with a linear increase in the number of consultations according to patients' age (from 5.2 annual consultations to 7.7 among the most deprived groups).
As of September 2013 there were 127 practices in Lothian[3]. Each practice would then have, on average, at least 1252 patients consulting during our intervention period (although numbers vary between practices).

Official Scottish Bowel screening data for the period 2011-2013 show that uptake in Lothian was 55.3% (ranging from 39.6% to 59.8% from the most deprived to the least deprived areas)[4]. Considering the participation rates for the most deprived areas (39.6%) and the population aged 50-74; there would roughly be a maximum of 756 eligible patients who could be reached during the study period per practice, or 4536 in six selected practices.

In practice, however, numbers are likely to be much smaller. For example, in 2011 the prevalence of cancer (all types) in Scotland was 4.5%[5]. These patients would not take part in the intervention as it would not be appropriate to approach the issue of cancer screening to someone in receipt of cancer treatment. Considering cancer prevalence in Scotland, the number of eligible patients would be reduced to 4355 in all practices. This is a conservative estimate as cancer is more common in areas of high deprivation (which is the case of some of the selected practices in Lothian). Furthermore, the opportunity to approach a patient about screening may not arise in every consultation. We estimate that an opportunity will arise in about a quarter of all consultations with eligible participants, thus reducing the number of patients to 1089 (or approximately 182 per practice).

References:

1.Scotland's Census 2011 - National Records of Scotland. Table DC1117SC - Age by sex. All people [Internet]. 2014. Available from: http://www.scotlandscensus.gov.uk.

2.Estimated number of patients seen1/not seen2 in Scotland by either a General Practitioner (GP) or Practice Nurse [Internet]. 2013. Available from: http://www.isdscotland.org/PTI/.

3 .General Practice – GP workforce and practice population statistics to 2013. Scottish general practice characteristics: as at 30th September from 2005 to 2013. In: ISD Scotland, editor. 2014.

4.Scottish Bowel Screening Programme. Scottish Bowel Screening Programme. Key Performance Indicators Report: May 2014 data submission. Invitations between 1st November 2011 and 31st October 2013. Scotland: Scottish Bowel Screening Programme; 2014.

5. ISD Scotland. Cancer in Scotland: ISD Scotland; 2012. Available from: https://isdscotland.scot.nhs.uk/Health-Topics/Cancer/Publications/2012-04-24/Cancer_in_Scotland_summary_m.pdf

For peer review only

B. Intervention supporting materials

Intervention material	Aim(s)	Theoretical framework/adopted principles
3-4 questions/statements<br>• A5 coloured laminated sheet of paper<br>• Given to the health care professional	• To guide discussion addressing concerns around or barriers to bowel screening with non-responders	• Non-directive statements<br>• Non-coercion<br>• Informed choice<br>• Implementation Intentions theory
Staff flowchart<br>• A4 coloured laminated sheet of paper<br>• Given to the health care professional	• To describe barriers to screening (with examples), and evidence-based approaches to deal with these barriers	• Health Behaviour Framework<br>• Motivational Interviewing
Patient leaflet and freepost envelope<br>• A4 coloured sheet of paper folded into A5, perforated so a reply slip could be sent back by post<br>• FREEPOST envelope addressed to the bowel screening centre<br>• Given to patients who accepted the intervention	• To deconstruct health beliefs associated with low uptake, i.e. the barriers described in the flowchart<br>• To provide space for the patient to develop their own implementation plan<br>• To offer the opportunity to request a new test kit	• Health Behaviour Framework<br>• Non-coercion<br>• Informed choice<br>• Implementation Intentions theory
Guidance sheet<br>• A4 coloured laminated sheet of paper<br>• Given to the health care professional	• To provide practice staff with relevant information regarding the research study	N/A
Practice Proforma<br>• Green A4 sheet with perforations (3 proformas in each)<br>• Given to the health care professional<br>• Designed to require approximately 2 minutes to be completed	• To collect relevant intervention data so the feasibility outcomes could be assessed:<br>o Intervention date, staff name and role, duration of the intervention, patient age and gender, reasons for consultation (text)<br>o Whether intervention was accepted, leaflet was given and completed in practice<br>o Comments	N/A

Note: Bowel Screening leaflets in Polish were requested from NHS Scotland and provided to practices with a larger Polish population

For peer review only

C. Feasibility parameters

- Processes required in general practice to record (and flag) responder status in patients' electronic records
- Number of patients approached during consultations
- Number of interventions delivered over the intervention period
- Length of time the intervention takes
- Number and willingness of patients to engage in conversation when responder status is raised during a consultation
- Number of patients who were willing to receive leaflet
- Number of patients who completed leaflet with health care professional
- Number of patients who sent reply slip
- Number of patients who returned a completed kit
- Processes required in the bowel screening centre to deal with the extra-to-programme returned FOBt kits, and to ensure over-screening does not occur
- Whether the brief intervention leads to longer discussion with patients regarding cancer screening, or bowel symptoms
- Willingness of GPs and practice nurses to deliver intervention
- Overall views on the study's feasibility according to primary care professionals
- Overall views on the study's feasibility according to bowel screening staff

For peer review only

Supplementary file 2

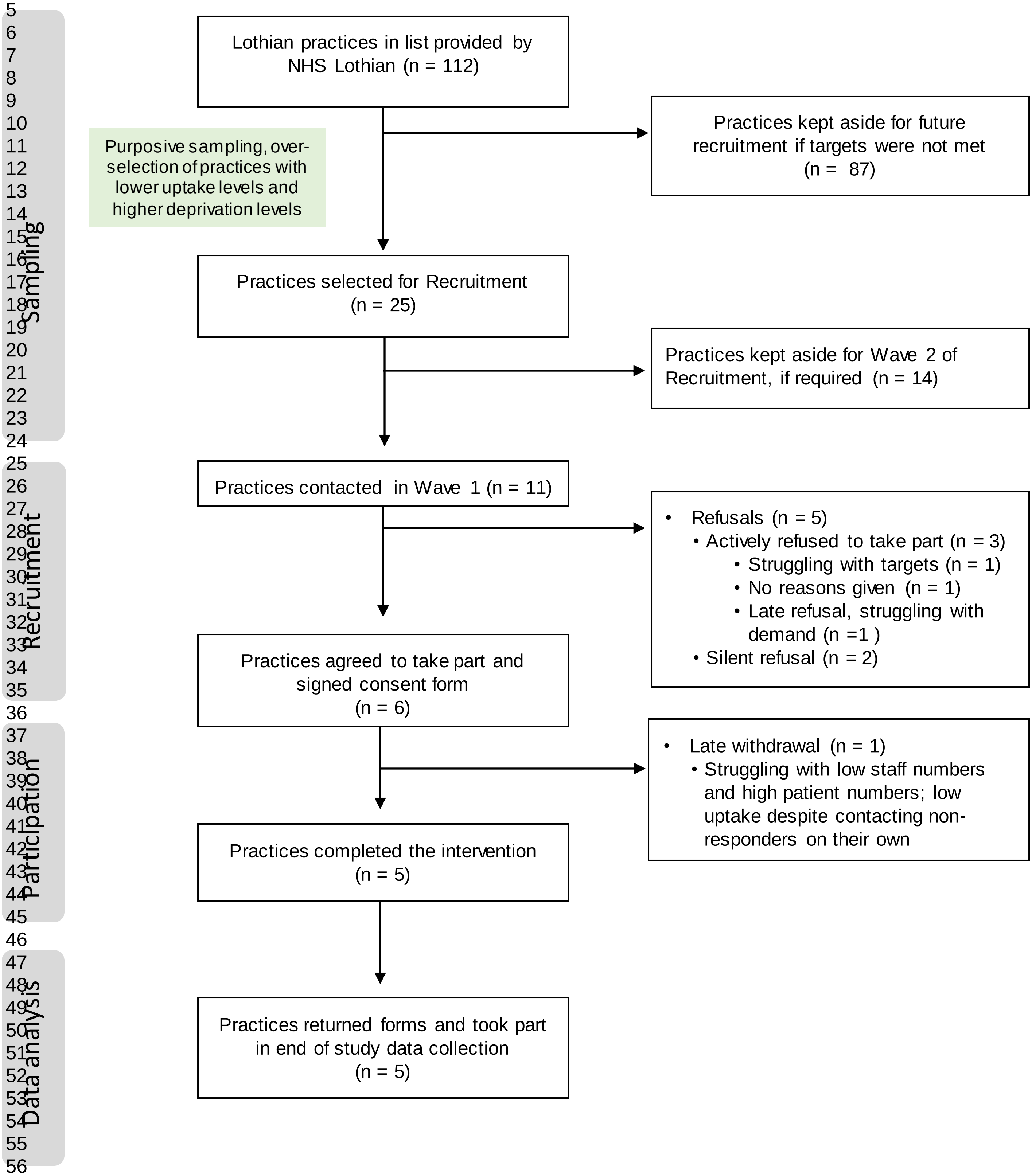

Supplementary file 3:

A. **Recorded reasons for consultation**
B. **Comparing patients who accepted and did not accept the intervention**

For peer review only

A. Recoded reasons for consultation

**Reasons for consultation**	**Practice A n(%)**	**Practice B n(%)**	**Practice C n(%)**	**Practice D n(%)**	**Practice E n(%)**	**All practices n(%)**			
	**All**	**All**	**All**	**All**	**All**	**GP**	**PN**	**HCA**	**All**
Known chronical illness/review of existing condition	25 (36.8)	6 (11.5)	2 (7.4)	18 (26.1)	4 (11.8)	12 (21.8)	40 (72.7)	3 (5.5)	55 (22.0)
Musculoskeletal symptoms/conditions	3 (4.4)	7 (13.5)	7 (25.9)	11 (15.9)	5 (14.7)	33 (100.0)	0 (0.0)	0 (0.0)	33 (13.2)
Tests/test results	8 (11.8)	8 (15.4)	3 (11.1)	8 (11.6)	2 (5.9)	4 (13.8)	17 (58.6)	8 (27.6)	29 (11.6)
Respiratory or ENT symptoms/conditions	6 (8.8)	7 (13.5)	2 (7.4)	4 (5.8)	3 (8.8)	14 (63.6)	8 (36.4)	0 (0.0)	22 (8.8)
Prescriptions/medication review	7 (10.3)	2 (3.8)	1 (3.7)	1 (1.4)	4 (11.8)	15 (100.0)	0 (0.0)	0 (0.0)	15 (6.0)
Skin complaints	1 (1.5)	5 (9.6)	2 (7.4)	3 (4.3)	4 (11.8)	13 (86.7)	2 (13.3)	0 (0.0)	15 (6.0)
Non-clinical	2 (2.9)	4 (7.7)	2 (7.4)	5 (7.2)	1 (2.9)	10 (71.4)	2 (14.3)	2 (14.3)	14 (5.6)
Gastrointestinal symptoms/conditions	3 (4.4)	4 (7.7)	1 (3.7)	4 (5.8)	2 (5.9)	14 (100.0)	0 (0.0)	0 (0.0)	14 (5.6)
Multiple issues	4 (5.9)	1 (1.9)	0 (0.0)	1 (1.4)	5 (14.7)	11 (100.0)	0 (0.0)	0 (0.0)	11 (4.4)
Mental health symptoms/conditions	0 (0.0)	2 (3.8)	2 (7.4)	2 (2.9)	1 (2.9)	7 (100.0)	0 (0.0)	0 (0.0)	7 (2.8)
Preventative behaviour	4 (5.9)	1 (1.9)	2 (7.4)	0 (0.0)	0 (0.0)	0 (0.0)	7 (100.0)	0 (0.0)	7 (2.8)
Gynaecological or urological symptoms/conditions	2 (2.9)	1 (1.9)	0 (0.0)	1 (1.4)	0 (0.0)	4 (100.0)	0 (0.0)	0 (0.0)	4 (1.6)
Inconclusive	0 (0.0)	0 (0.0)	0 (0.0)	2 (2.9)	0 (0.0)	2 (100.0)	0 (0.0)	0 (0.0)	2 (0.8)
Other/non-specific symptoms	3 (4.4)	4 (7.7)	3 (11.1)	9 (13.0)	3 (8.8)	21 (95.5)	1 (4.5)	0 (0.0)	22 (8.8)
Total	68 (100.0)	52 (100.0)	27 (100.0)	69 (100.0)	34 (100.0)	N/A	N/A	N/A	250 (100.0)

Abbreviations: GP: General Practitioner; PN: Practice Nurse; HCA – Health Care Assistant; ENT – ear, nose and throat
Missing data: 8 cases (3 in Practice A, 1 in Practice C, 1 in Practice D and 3 in Practice B).
Sums may not add up to 100 due to rounding

B. Comparing patients who accepted and did not accept the intervention

Overall data	Accepted the intervention (n=220) n(%)	Did not accept the intervention (n=33) n(%)	Overall (n=253)* n(%)
**Patient sex**			
Male	113 (51.4)	22 (66.7)	135 (53.4)
Female	107 (48.6)	11 (33.3)	118 (46.6)
**Patient age**			
Median (IQR)	58.00 (53.00-64.75)	64.00 (57.00-71.50)	58.00 (53.00-65.00)
50-54	78 (35.5)	3 (9.1)	81 (32.5)
55-59	48 (21.8)	9 (27.3)	57 (22.9)
60-64	36 (16.4)	6 (18.2)	42 (16.9)
65-69	32 (14.5)	3 (9.1)	35 (14.1)
70-74	17 (7.7)	9 (27.3)	26 (10.4)
75-79	5 (2.3)	3 (9.1)	8 (3.2)
**Staff carrying out the intervention**			
GP	153 (69.5)	11 (33.3)	164 (64.8)
PN	57 (25.9)	19 (57.6)	76 (30.0)
HCA	10 (4.5)	3 (9.1)	13 (5.1)
**Duration of the intervention**			
Median (IQR)	2.00 (1.00-5.00)	2.00 (2.00-5.00)	2.00 (1.00-5.00)
**Reasons for consultation**			
Known chronical illness/review of existing condition	42 (19.7)	12 (37.5)	54 (22.0)
Musculoskeletal symptoms/conditions	30 (14.1)	3 (9.4)	33 (13.5)
Tests/test results	22 (10.3)	5 (15.6)	27 (11.0)
Respiratory or ENT symptoms/conditions	17 (8.0)	5 (15.6)	22 (9.0)
Prescriptions/medication review	15 (7.0)	0 (0.0)	15 (6.1)
Skin complaints	15 (7.0)	0 (0.0)	15 (6.1)
Non-clinical	14 (6.6)	0 (0.0)	14 (5.7)
Gastrointestinal symptoms/conditions	12 (5.6)	1 (3.1)	13 (5.3)
Multiple issues	10 (4.7)	1 (3.1)	11 (4.5)
Mental health symptoms/conditions	6 (2.8)	1 (3.1)	7 (2.9)
Preventative behaviour	5 (2.3)	1 (3.1)	6 (2.4)
Gynaecological or urological symptoms/conditions	2 (0.9)	2 (6.3)	4 (1.6)
Inconclusive	2 (0.9)	0 (0.0)	2 (0.8)
Other/non-specific symptoms	21 (9.9)	1 (3.1)	22 (9.0)

Abbreviations: GP: General Practitioner; PN: Practice Nurse; HCA – Health Care Assistant; ENT – ear, nose and throat; IQR – Interquartile range
*In 5 cases data were missing on whether patient accepted the intervention. These cases are not included here; hence the overall values do not match the ones in the main manuscript (i.e. 258 participants). There were also missing data for patient age (4 cases), duration of intervention (9 cases) and reasons for consultation (8 cases). Sums may not add up to 100 due to rounding.

For peer review only

Supplementary file 4

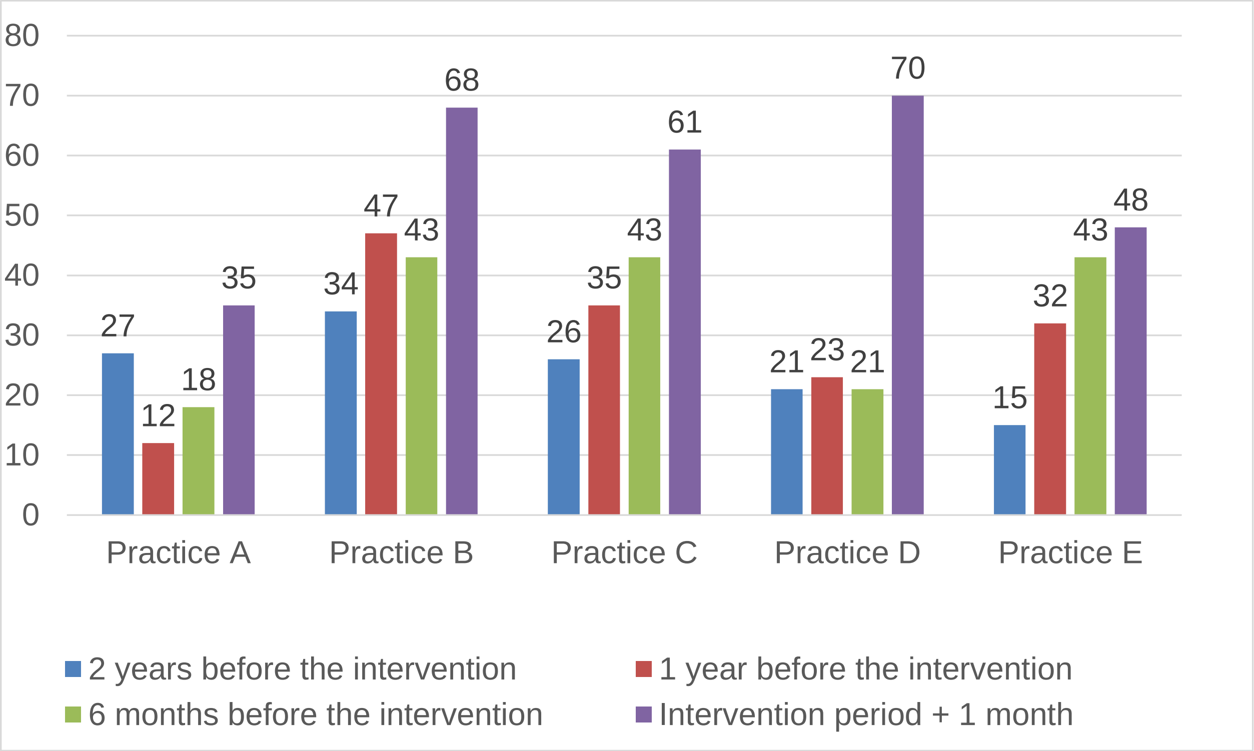

STROBE 2007 (v4) Statement—Checklist of items that should be included in reports of *cross-sectional studies*

Section/Topic	Item #	Recommendation	Reported on page #
**Title and abstract**	1	(*a*) Indicate the study's design with a commonly used term in the title or the abstract	1
		(*b*) Provide in the abstract an informative and balanced summary of what was done and what was found	2
**Introduction**			
Background/rationale	2	Explain the scientific background and rationale for the investigation being reported	3,4
Objectives	3	State specific objectives, including any prespecified hypotheses	4
**Methods**			
Study design	4	Present key elements of study design early in the paper	5,6
Setting	5	Describe the setting, locations, and relevant dates, including periods of recruitment, exposure, follow-up, and data collection	4,5,8
Participants	6	(*a*) Give the eligibility criteria, and the sources and methods of selection of participants	4,5
Variables	7	Clearly define all outcomes, exposures, predictors, potential confounders, and effect modifiers. Give diagnostic criteria, if applicable	6
Data sources/ measurement	8*	For each variable of interest, give sources of data and details of methods of assessment (measurement). Describe comparability of assessment methods if there is more than one group	7
Bias	9	Describe any efforts to address potential sources of bias	Limitations and potential sources of bias approached in the discussion (page 17)
Study size	10	Explain how the study size was arrived at	4,5
Quantitative variables	11	Explain how quantitative variables were handled in the analyses. If applicable, describe which groupings were chosen and why	7
Statistical methods	12	(*a*) Describe all statistical methods, including those used to control for confounding	7
		(*b*) Describe any methods used to examine subgroups and interactions	N/A
		(*c*) Explain how missing data were addressed	Reported in all cases (table footnotes on pages 9 and 10, supplementary file 3). N/A for multivariate analysis as only descriptive statistics were reported
		(*d*) If applicable, describe analytical methods taking account of sampling strategy	N/A

For peer review only

		(*e*) Describe any sensitivity analyses	N/A
**Results**			
Participants	13*	(a) Report numbers of individuals at each stage of study—eg numbers potentially eligible, examined for eligibility, confirmed eligible, included in the study, completing follow-up, and analysed	8, 9, 10, 11 and supplementary file 3
		(b) Give reasons for non-participation at each stage	Not available, limitations approached on page 17
		(c) Consider use of a flow diagram	Supplementary file 2
Descriptive data	14*	(a) Give characteristics of study participants (eg demographic, clinical, social) and information on exposures and potential confounders	8, 9, 10, 11, supplementary file 3, limitations discussed in pages 16 and 17
		(b) Indicate number of participants with missing data for each variable of interest	Reported in all cases (table footnotes on pages 9 and 10, supplementary file 3).
Outcome data	15*	Report numbers of outcome events or summary measures	8, 9, 10, 11
Main results	16	(*a*) Give unadjusted estimates and, if applicable, confounder-adjusted estimates and their precision (eg, 95% confidence interval). Make clear which confounders were adjusted for and why they were included	N/A, no multivariate analyses were carried out
		(*b*) Report category boundaries when continuous variables were categorized	N/A
		(*c*) If relevant, consider translating estimates of relative risk into absolute risk for a meaningful time period	N/A
Other analyses	17	Report other analyses done—eg analyses of subgroups and interactions, and sensitivity analyses	Supplementary file 3
**Discussion**			
Key results	18	Summarise key results with reference to study objectives	16
Limitations	19	Discuss limitations of the study, taking into account sources of potential bias or imprecision. Discuss both direction and magnitude of any potential bias	17
Interpretation	20	Give a cautious overall interpretation of results considering objectives, limitations, multiplicity of analyses, results from similar studies, and other relevant evidence	16, 17, 18
Generalisability	21	Discuss the generalisability (external validity) of the study results	17
**Other information**			
Funding	22	Give the source of funding and the role of the funders for the present study and, if applicable, for the original study on which the present article is based	19

*Give information separately for cases and controls in case-control studies and, if applicable, for exposed and unexposed groups in cohort and cross-sectional studies.

Note: An Explanation and Elaboration article discusses each checklist item and gives methodological background and published examples of transparent reporting. The STROBE checklist is best used in conjunction with this article (freely available on the Web sites of PLoS Medicine at http://www.plosmedicine.org/, Annals of Internal Medicine at http://www.annals.org/, and Epidemiology at http://www.epidem.com/). Information on the STROBE Initiative is available at www.strobe-statement.org.

For peer review only

BMJ Open

Is an opportunistic primary care-based intervention for non-responders to bowel screening feasible and acceptable? A mixed methods feasibility study in Scotland

Journal:	*BMJ Open*
Manuscript ID	bmjopen-2017-016307.R1
Article Type:	Research
Date Submitted by the Author:	07-Jun-2017
Complete List of Authors:	Calanzani, Natalia ; University of Edinburgh, Centre For Population Health Sciences, The Usher Institute of Population Health Sciences and Informatics<br>Cavers, Debbie; University of Edinburgh, General Practice<br>Vojt, Gabriele; Glasgow Caledonian University, Department of Psychology<br>Orbell, Sheina; University of Essex, Department of Psychology<br>Steele, Robert; University of Dundee, Surgery and Molecular Oncology<br>Brownlee, Linda; Scottish Bowel Screening Centre<br>Smith, Steve; University Hospitals of Coventry & Warwickshire NHS Trust, Midlands & NW Bowel Cancer Screening Hub, Hospital of St. Cross<br>Patnick, Julietta; University of Oxford, Cancer Epidemiology Unit<br>Weller, David; University of Edinburgh, General Practice<br>Campbell, Christine; University of Edinburgh, Centre for Population Health Sciences
**Primary Subject Heading**:	Health services research
Secondary Subject Heading:	General practice / Family practice, Public health
Keywords:	general practice, neoplasms, feasibility studies, bowel screening

SCHOLARONE™
Manuscripts

Is an opportunistic primary care-based intervention for non-responders to bowel screening feasible and acceptable? A mixed methods feasibility study in Scotland

Natalia Calanzani[1], Debbie Cavers[1], Gabriele Vojt[2], Sheina Orbell[3], Robert JC Steele[4], Linda Brownlee[5], Steve Smith[6], Julietta Patnick[7], David Weller[1], Christine Campbell[1]

[1]University of Edinburgh, The Usher Institute of Population Health Sciences and Informatics, Centre for Population Health Sciences, Medical School, Teviot Place, Edinburgh EH8 9AG, UK

[2]Glasgow Caledonian University, Department of Psychology, Cowcaddens Road, Glasgow G4 0BA

[3]Department of Psychology, University of Essex, Wivenhoe Park, Colchester CO4 3SQ, UK

[4]Surgery and Molecular Oncology, Ninewells Hospital, University of Dundee, Dundee, DD1 9SY, Scotland, UK

[5]Scottish Bowel Screening Centre, Kings Cross, Clepington Road, Dundee, DD3 8EA, Scotland, UK

[6]University Hospitals of Coventry & Warwickshire NHS Trust, Midlands & NW Bowel Cancer Screening Hub, Hospital of St. Cross, Barry Road, Rugby, CV22 5PX

[7]Oxford University, Cancer Epidemiology Unit, Richard Doll Building, Roosevelt Drive, Oxford OX3 7LF

Word count: 4,546

Corresponding author:

Natalia Calanzani

Centre for Population Health Sciences

The Usher Institute of Population Health Sciences and Informatics, University of Edinburgh

Room 123, Doorway 1, Medical School, Teviot Place, Edinburgh EH8 9AG

Tel: 0131 650 3818

Fax: 031 650 3227

Natalia.Calanzani@ed.ac.uk

For peer review only

For peer review only - http://bmjopen.bmj.com/site/about/guidelines.xhtml

ABSTRACT

Objectives: We aimed to test whether a brief, opportunistic intervention in general practice was a feasible and acceptable way to engage with bowel screening non-responders.

Design: This was a feasibility study testing an intervention which comprised a brief conversation during routine consultation, provision of a patient leaflet and instructions to request a replacement faecal occult blood test kit. A mixed methods approach to evaluation was adopted. Data were collected from proformas completed after each intervention, from the Bowel Screening Centre database, and from questionnaires. Semi-structured interviews were carried out. We used descriptive statistics, content and framework analysis to determine intervention feasibility and acceptability.

Participants: Bowel screening non-responders (as defined by the Scottish Bowel Screening Centre) and primary care professionals working in five general practices in Lothian, Scotland.

Primary and secondary outcome measures: Several predefined feasibility parameters were assessed, including numbers of patients engaging in conversation, requesting a replacement kit and returning it; and willingness of primary care professionals to deliver the intervention.

Results: The intervention was offered to 258 patients in five general practices: 220 (87.0%) engaged with the intervention, 60 (23.3%) requested a new kit, 22 (8.5%) kits were completed and returned. Interviews and questionnaires suggest that the intervention was feasible, acceptable, and consistent with an existing health prevention agenda. Reported challenges referred to work-related pressures, time constraints and practice priorities.

Conclusions: This intervention was acceptable and resulted in a modest increase in non-responders participating in bowel screening, although outlined challenges may affect sustained implementation. The strategy is also aligned with the increasing role of primary care in promoting bowel screening.

Keywords: feasibility studies; bowel screening; general practice; neoplasms

For peer review only - http://bmjopen.bmj.com/site/about/guidelines.xhtml

STRENGTHS AND LIMITATIONS OF THE STUDY

- This intervention is grounded in psychological theory and evidence on factors associated with non-participation in bowel screening
- Furthermore, it considered the pragmatic reality of a dynamic, time-pressured primary care environment
- This is a small-scale, non-randomised feasibility study in one region of Scotland, targeting non-responders who consult in primary care
- As the intervention is of an opportunistic nature, data on patient characteristics (such as medical history or ethnicity) are not available

INTRODUCTION

Bowel screening using a faecal occult blood test (FOBt) enables identification of earlier stage cancers when treatment is more likely to be beneficial [1], ultimately leading to reduction in bowel cancer mortality [2]. The United Kingdom has well-established bowel screening programmes in each of its constituent countries. The Scottish Bowel Screening Centre (SBSC) sends a guaiac-based FOBt biennially to eligible patients aged 50-74 years [3]. The current uptake is 57.7%; with lower participation among the most deprived populations compared with the least deprived groups (45.5% vs 66.6% respectively). Uptake is higher for women (60.6%) compared to men (54.7%) [4].

Both barriers to uptake and effective strategies to increase participation in bowel screening are described in the literature. Lack of awareness of bowel cancer [5] or of screening [6], concerns about unpleasantness and embarrassment [7, 8], fear of the outcome [5, 9], fatalism [10] and perception of risk [11, 12] are commonly identified barriers. Nevertheless, there is good evidence that reminders targeting patients [13, 14] and physicians [5], having one-on-one interactions/education with general practitioners (GPs) and/or nurses [6, 15] and GP endorsements [6, 16] have a positive impact on screening uptake.

Primary care has an important and increasing role in cancer prevention and cancer screening [17]. In the UK alone, a number of interventions involving primary care have been recently developed [1, 13, 16, 18, 19]. In Scotland, a government programme aiming to improve cancer survival (the Detect Cancer Early Programme) [20] provided a financial incentive for practices meeting defined bowel screening targets [21, 22].

For peer review only - http://bmjopen.bmj.com/site/about/guidelines.xhtml

In this context, we aimed to test the feasibility and acceptability of an opportunistic intervention in general practice patient consultations, examining whether a brief conversation was a viable way to engage with non-responders and increase bowel screening participation. The study was undertaken in the Lothian region of Scotland which has slightly lower bowel screening uptake (57.2%) than the national average, and shows similar variation based on sex and socio-economic status [4].

MATERIALS AND METHODS

Recruitment of Practices and Patients

Practice recruitment

NHS Lothian provided the research team with a list of 112 practices in this region. The list had information on practice code, % screening uptake in 2013, practice list size, number of patients aged 50-75, number of average monthly non-responders, mean Scottish Index of Multiple Deprivation (SIMD) decile [23] (for those aged 50-75), and whether or not practices took part in the bowel Scottish Quality and Outcomes Framework (SQoF) [21]. Eleven general practices were purposively selected for a first wave of recruitment. We oversampled among the most deprived practices with lower uptake (as it was perceived that these practices could benefit the most from the intervention), while also taking into account the other factors listed above (as these would impact on how many patients could potentially be approached during the study period). Practices were invited to take part in the study via a personalised email sent by the study's principal investigator.

A visit was scheduled at the practices that were interested in taking part in the study. A brief information session was delivered, giving background information on colorectal cancer and screening, known barriers and facilitators to screening uptake and a thorough description of the study. Practices also received a folder containing the intervention materials, study information sheet, ethical approvals, background information on bowel cancer and bowel screening and a consent form.

Estimated size of study population

A preliminary calculation estimated that each recruited general practice would have the opportunity to engage with up to 182 potentially eligible patients during the study period (Supplementary File 1). These figures are rough estimates as the study findings will guide calculations of a powered sample size for a larger study. We aimed to recruit up to six general practices for testing the intervention's feasibility and acceptability.

For peer review only - http://bmjopen.bmj.com/site/about/guidelines.xhtml

Patient inclusion and exclusion criteria

The target population were men and women aged 50-74 registered in a participating Lothian practice who received an invitation to screening and did not return a completed kit with a definitive screening result within 90 days (i.e. the official SBSC's definition of a non-responder). Patients were excluded from the study when they lacked mental capacity as defined by the Mental Capacity Act 2005 [24] and at professionals' discretion where patients were regarded as too ill (e.g. undergoing cancer treatment or in receipt of palliative care) or distressed to take part.

Identifying bowel screening non-responders

NHS Lothian and the SBSC routinely provide Scottish general practices with a list of non-responders. The research team worked alongside each participating practice to create a customised plan to ensure they could efficiently flag non-responders in their computer systems if they did not already have a system in place.

Intervention

The intervention comprised a brief conversation about bowel screening with non-responders. During a consultation with an eligible patient, the primary care professional (PCP) (a GP, practice nurse or health care assistant) raised the topic of non-participation using neutral statements and discussed any patient concerns. A leaflet with further information and an opportunity to request a bowel screening kit (via email, phone or tear off slip with FREEPOST) was offered. The intervention was designed to last 3-5 minutes. As part of the intervention, patients could also choose whether or not to develop a written plan of how to complete and return the kit (an implementation intention) [25]. In addition to the information leaflet and FREEPOST envelope, the intervention was supported by: an A5 set of 3-4 suggested questions/topic for discussion; an intervention flowchart and guidance sheet for PCPs (Supplementary File 1).

In developing the intervention content we drew on our previous work on strategies promoting uptake of FOBt screening [26], and available literature on factors associated with uptake and barriers to screening. We also drew on psychological models, principally on Implementation Intentions [25], the Health Behaviour Framework [27], and were guided by principles of motivational interviewing [28] and informed choice [29]. We chose the Health Behaviour Framework as it synthesises major health behaviour models while also considering contextual factors. It has also been successfully applied to cancer screening health behaviours [30-32].

For peer review only - http://bmjopen.bmj.com/site/about/guidelines.xhtml

Implementation Intentions have been associated with higher participation in studies about cervical [33], breast [34] and bowel [35] screening. Finally, motivational interviewing has been shown to increase bowel screening uptake [36, 37]; its principles aided the development of non-directive statements to discuss non-participation.

The process of developing the intervention also included eight interviews with health professionals and 19 non-responders to bowel screening to explore their views on its acceptability in a primary care setting. Overall feedback was positive and results are reported elsewhere (manuscript under review). We sought approaches (and wording in our materials) which were not coercive, but invited participants to consider the offer of screening after balancing potential benefits and harms. Materials conformed to the Scottish Bowel Screening Programme [38] and the NHS Cancer Screening Programme [39] resources and guidance.

Data Collected for Evaluation

Delivery of the intervention

PCPs logged details of each intervention on a proforma (Supplementary File 1). Researchers regularly visited practices to collect these and to distribute materials as required, recording all communication/events in an intervention log. The intervention was planned to run for 3-4 months in each practice, depending on availability.

Requests for screening kits

Requests for new kits were made to the SBSC, which logged both the requests using the tear-off slip, and the returned kits. It was not possible to identify email or telephone requests relating to this project due to the high volume received through these means daily. For the purposes of comparison, the SBSC also provided data on total number of requests for a replacement kit made by each of the recruited practices during the intervention period (plus one extra month to allow time for requests to come in), and for equivalent periods at six months, one year and two years before the intervention.

Questionnaire and Interview Data

The feasibility parameters and mechanisms to be investigated are available in Supplementary File 1. Brief end of study questionnaires were developed for primary care and bowel screening staff. The questionnaires comprised closed and open-ended questions and focused on intervention acceptability and potential impact on workload. Semi-structured interviews were carried out with members of the practice team (aiming to

For peer review only - http://bmjopen.bmj.com/site/about/guidelines.xhtml

interview the practice manager and at least one GP or practice nurse). Interviews sought to ascertain views on the running of the brief intervention, its acceptability, and its overall feasibility as part of routine primary care.

Data analysis

Templates were created using SPSS 19 for Windows [40] to collect data on the practice proforma and the end of study questionnaires. Quantitative data from the SBSC, proformas and end of study questionnaires were analysed using descriptive statistics (summaries, frequencies and cross-tabulations). As we had a purposeful sample of practices and a non-random sample of patients, no inferential statistics were calculated [41].

End of study interviews were digitally recorded and transcribed verbatim. Qualitative data were analysed using thematic analysis informed by a framework approach including techniques of familiarisation, coding, indexing, charting, mapping and interpretation [42, 43] assisted by QSR NVivo 7 software [44]. This approach was considered appropriate due to the pre-existing feasibility parameters being tested. Identified themes were considered in the context of these parameters and interpreted according to existing theory and research. Scrutiny both within and across transcripts ensured that the analysis encompassed all perspectives and used the whole dataset. All transcripts were read by two researchers (DC and NC) and 50% were subject to triple initial coding (DC, NC and CC). The initial coding framework was developed by DC and was reiterated following discussion, with any discrepancies explored and accounted for. Content analysis [45] was used to summarise, categorise and interpret text entries made by practice staff on proformas to record reasons for consultation.

Ethical approval and consent

The study was approved by the South East Scotland Research Ethics Committee 01 (reference 14/SS/1067) and the NHS Lothian's Research and Development Office (Project Number 2014/0366). The Scottish Bowel Screening Governance Reference Group also approved the study. Written informed consent was obtained via the practice manager or GP partner and separate consent was obtained for end of study interviews. Practices were reimbursed for their participation.

RESULTS

Practice recruitment

For peer review only - http://bmjopen.bmj.com/site/about/guidelines.xhtml

Six out of 11 invited practices consented to participate in the study; one practice subsequently withdrew due to resource issues (Supplementary file 2). The five remaining practices varied in size, bowel screening uptake and deprivation levels (Table 1). All but one practice were signed up to the SQoF.

Table 1 Characteristics of recruited practices

Recruited Practices	Location	Uptake % (2013)	Pop 50-75	Mean SIMD decile (50-75 year olds)*	Average monthly non-responders	Practice list size	Signed up to SQoF	Start date	End date
Practice A	Edinburgh	<45%	1,413	2.6	41	6,888	No	04/03/15	05/07/15
Practice B	Edinburgh	45-50%	2,654	3.6	61	10,440	Yes	14/04/15	15/08/15
Practice C	East Lothian	50-55%	2,515	4.5	56	8,693	Yes	22/04/15	03/09/15
Practice D	Edinburgh	50-55%	1,241	6.2	29	5,326	Yes	20/04/15	24/08/15
Practice E	Midlothian	55-60%	1,668	5.5	30	5,201	Yes	05/03/15	08/07/15

Abbreviations: Pop: population; SIMD: Scottish Index of Multiple Deprivation; SQoF; Scottish Quality and Outcomes Framework.
*The Scottish Index of Multiple Deprivation (SIMD) is a measure of multiple deprivation which combines different domains related to employment, income, health, education, skills and training, geographic access to services, crime and housing [23]. The lower the decile number, the higher the deprivation levels.

Setting up the intervention

Although all practices were routinely provided with an electronic list of non-responders, there was variation in the methods in place to identify non-responders during consultations. In two practices, the researchers coded non-responders into practices' computer systems, also helping to insert screen 'pop-up' reminders in one of these cases. The remaining three practices already had systems in place.

One practice developed a digital proforma in their GP system instead of using the paper-based one provided by the research team. Planned monthly visits were not always required and were adapted to suit practice needs.

Intervention delivery, acceptance and impact on screening uptake

Overall, 258 patients were approached between March and September 2015. Men were approached slightly more often than women (53.1% vs 46.9%) and most patients were among the younger eligible age groups for screening (median 58.00, interquartile range (IQR) 53.00-65.00) (Table 2). No information on patient ethnicity was available. The median duration of the intervention was 2.00 minutes (IQR 1.25-5.00).

Table 2. Patient and staff characteristics

Overall data	Practice A	Practice B	Practice C	Practice D	Practice E	Total

For peer review only - http://bmjopen.bmj.com/site/about/guidelines.xhtml

	n(%)	n(%)	n(%)	n(%)	n(%)	n(%)
**Patient sex**						
Men	43 (60.6)	25 (45.5)	8 (28.6)	43 (61.4)	18 (52.9)	137 (53.1)
Women	28 (39.4)	30 (54.5)	20 (71.4)	27 (38.6)	16 (47.1)	121 (46.9)
Total	71 (100.0)	55 (100.0)	28 (100.0)	70 (100.0)	34 (100.0)	258 (100.0)
**Patient age[a]**						
Median	55.50	55.50	58.50	63.00	59.00	58.00
(IQR)	(53.00-64.00)	(51.75-62.25)	(52.25-67.75)	(56.25-69.00)	(53.75-63.00)	(53.00-65.00)
50-54	25 (35.7)	24 (44.4)	10 (35.7)	13 (19.1)	11 (32.4)	83 (32.7)
55-59	22 (31.4)	9 (16.7)	5 (17.9)	14 (20.6)	7 (20.6)	57 (22.4)
60-64	8 (11.4)	10 (18.5)	4 (14.3)	11 (16.2)	11 (32.4)	44 (17.3)
65-69	6 (8.6)	6 (11.1)	4 (14.3)	15 (22.1)	5 (14.7)	36 (14.2)
70-74	9 (12.9)	4 (7.4)	3 (10.7)	10 (14.7)	0 (0.0)	26 (10.2)
75-79	0 (0.0)	1 (1.9)	2 (7.1)	5 (7.4)	0 (0.0)	8 (3.1)
Total	70 (100.0)	54 (100.0)	28 (100.0)	68 (100.0)	34 (100.0)	254 (100.0)
**Interventions by primary care role**						
GP	33 (46.5)	31 (56.4)	21 (75.0)	50 (71.4)	31 (91.2)	166 (64.3)
PN	38 (53.5)	11 (20.0)	7 (25.0)	20 (28.6)	3 (8.8)	79 (30.6)
HCA	0 (0.0)	13 (23.6)	0 (0.0)	0 (0.0)	0 (0.0)	13 (5.0)
Total	71 (100.0)	55 (100.0)	28 (100.0)	70 (100.0)	34 (100.0)	258 (100.0)

Abbreviations: GP: General Practitioner; PN: Practice Nurse; HCA: Health Care Assistant.
[a]Eight patients aged 75 or older were included as their last invitation to screening happened before their 75th birthday (hence meeting eligibility criteria).
Missing data: There were no missing data for patient sex and staff carrying out the intervention. There were 4 missing cases for patient age. Percentages may not add up to 100 due to rounding.

The majority of the interventions were carried out by GPs (64.3%), followed by practice nurses (30.6%) and health care assistants (5.0%). Patients receiving the intervention consulted for a variety of reasons (Supplementary File 3). The main reasons were reviews of existing conditions (22.0%), consultations due to musculoskeletal symptoms/conditions (13.2%), to carry out tests or obtain test results (11.6%), or due to respiratory or ear, nose and throat symptoms/conditions (8.8%).

The majority of patients who were offered the intervention accepted it (i.e. engaged in conversation) (87.0%), with variations across practices (Table 3). The leaflet was given to 74.2% of patients. Almost a quarter of the 258 patients approached (n=60) requested a replacement kit using a reply slip. Over a third of these patients (n=22) also returned a completed kit.

For peer review only

For peer review only - http://bmjopen.bmj.com/site/about/guidelines.xhtml

Table 3. Intervention acceptability, requested and returned kits

Overall data		Practice A n(%)	Practice B n(%)	Practice C n(%)	Practice D n(%)	Practice E n(%)	Total n(%)
Duration of intervention (minutes)[a]	Median (IQR)	3.50 (2.00-5.00)	2.00 (2.00-4.50)	2.00 (1.00-5.00)	2.00 (2.00-3.25)	1.00 (1.00-2.00)	2.00 (1.25-5.00)
Acceptance of intervention	Accepted (yes)	56 (78.9)	46 (85.2)	24 (96.0)	61 (88.4)	33 (97.1)	220 (87.0)
	Leaflet given (yes)	40 (57.1)	44 (81.5)	24 (85.7)	48 (68.6)	34 (100.0)	190 (74.2)
	Leaflet completed in practice (yes)	10 (14.3)	9 (16.7)	0 (0.0)	19 (27.9)	0 (0.0)	38 (17.3)
Requested kits using a slip	N requested kits/total interventions (% requesting a kit amongst total interventions)	16/71 (22.5)	7/55 (12.7)	6/28 (21.4)	25/70 (35.7)	6/34 (17.7)	60/258 (23.3)
Returned kits	N completed kits returned/total requested kits (% completing kits amongst total requests)	1/16 (6.3)	4/7 (57.1)	5/6 (83.3)	8/25 (32.0)	4/6[c] (66.7)	22/60 (36.7)
Test results	Negative	1	3	4	8	4	20
	Positive	0	0	1	0	0	1
	Pending[b]	0	1	0	0	0	1
Non-responders approached who became a responder to screening	N completed kits/N approached non-responders (% approached who became a responder)	1/71 (1.4)	4/55 (7.2)	5/28 (17.9)	8/70 (11.4)	4/34 (11.8)	22/258 (8.5)
Non-responders accepting the intervention who became a responder to screening	N completed kits/N accepting intervention (% accepting intervention who became a responder)	1/56 (1.8)	4/46 (8.7)	5/24 (20.8)	8/61 (13.1)	4/33 (12.1)	22/220 (10.0)

Abbreviations: IQR: Interquartile Range.
Missing data: there were 10 missing cases for duration of intervention, 5 for whether intervention was accepted, 2 for whether leaflet was given and 4 for whether it was completed in the practice. The same denominator (i.e. the total number of interventions carried out) applies for each question about acceptance of the intervention (i.e. intervention accepted, leaflet given and leaflet completed in practice) due to issues observed in data entry. Overall 4 leaflets were given although intervention was ticked as not accepted and 12 leaflets were completed in practice although they were ticked as not given to the patient.
[a]Over 90% of the interventions (n=225) lasted up to 5 minutes.
[b]A weak positive result (not shown) indicates that further tests are needed; in one case results for further tests were not yet available so results are shown as pending. In another case a weak positive became a positive result after further tests.
[c]One patient from Practice E requested a kit but was not sent one as s/he was only due for a new test in 2016. The National Bowel Screening System (BoSS) does not allow for sending additional kits for patients who are not due for another test; this helps to avoid over-screening.

For peer review only - http://bmjopen.bmj.com/site/about/guidelines.xhtml

Younger participants were more likely to accept the intervention (median age 58.00; IQR 53.00-64.75 for those accepting the intervention vs. 64.00; IQR 57.00-71.50 for those not accepting it). Men refused the intervention more often than women (the former represented 66.7% of all refusals). Over half (57.6%) of refused interventions were carried out by a practice nurse (Supplementary File 3).

Descriptive data from the Bowel Screening Centre on requested kits (Supplementary File 4) show that there was an increase in the number of requested kits across all practices during the intervention period (the highest increase in practice D and the lowest in Practice E) compared to two years, one year and six months prior to the intervention.

End of study evaluation: qualitative interviews

Eleven individual and one group in-depth qualitative interviews were conducted with a total of 14 primary care staff (four GPs, four practice nurses, five practice managers and one health care assistant). Thirteen interviews were face to face and one was via telephone. Findings from the qualitative interviews identified four main domains: the primary care and general *health care context*; the *processes* involved *in delivering the intervention*; *patient-related acceptability*; and *primary care professional acceptability* (Table 4).

For peer review only - http://bmjopen.bmj.com/site/about/guidelines.xhtml

Table 4. End of study interview quotes to support findings

1.Health care context

Existing practices

- We remind patients about cervical screening, so it's on a, sort of, slightly similar vein." Practice Nurse, Practice D
- "I think similar to our alcohol brief interventions and I think it enables us to initiate conversation…about an important subject which we might not otherwise do." GP, Practice D

Pressurised work environment

- "Because of the state of the practice at the minute when we've got doctors leaving - retiring and resigning – it's just put an added burden on existing people to do that." Practice Manager, Practice C
- "Just part of a greater workload issue. We're struggling to provide our contracted services, so I'm not going to commit to take on anything now unless it's properly resourced." GP, Practice E

Acknowledging barriers to screening

- "But it's always practicalities. That's why people don't want to do it." Practice Nurse, Practice D
- It's interesting because the research does show there is a kind of gradient there and that in some minority groups the uptake is not as high." GP, Practice D

Knowledge of and attitude to bowel screening

- It's definitely an extra to add in to the patients but I'm a real proponent of preventive health care and I think these things are worthwhile." GP, Practice D
- "To be honest, the practice population that we have have far bigger problems than whether they did their bowel screening or not, so in the real world it's possibly not one of the things we would include." Practice Nurse, Practice A

2. Processes in implementing the intervention

Providing information on the intervention

- "Actually it was very helpful, because I hadn't understood what the patients were being asked to do." GP, Practice E
- "They all thought it was a very worthwhile thing to sign up to." Practice Manager, Practice C

Appropriate timing and scenarios for the intervention

- "Probably if you have had any consultations that has presented with six problems and the last thing you need is to get into something else." GP, Practice B
- "I think when you leave it to the end you have not encroached on the patient's time." Practice Nurse, Practice B

Use of intervention supporting materials

- "You maybe look at it once or twice and see what's the kind of chat and then you probably don't dig it out every time, it's so opportunistic. GP, Practice A

Minimising paperwork, adaptations and integrating the intervention into existing IT

- "Obviously if we had a reminder for everything […] then we wouldn't be able to see the screen for reminders. So it's okay in the short term but in the long term it's a bit more difficult." Practice Manager, Practice A
- "We've got a computer, so it tells you that you need a bowel intervention, so why (not) record the data that you wanted on the same system?" GP, Practice E

Time limitations

- "I was aware that we were missing lots of people as the GPs simply didn't have time." Practice Manager, Practice C
- "In GP land when you've got ten minutes, ten minutes, ten minutes, then every little five minutes counts." GP, Practice A

Constraints in implementing the intervention

- "It all boils down to sometimes some men don't want to discuss it. […] I've found that sometimes a barrier, especially with older men." Practice Nurse, Practice D
- "We have a high Asian population and they are not keen to talk about poo or the practicalities of keeping their kit beside the toilet." GP, Practice D

Translating intention into action

- "When you actually spoke about it they thought it was a good idea, "I'll do it", but whether they do it or not, don't know." Practice Nurse, Practice B

For peer review only - http://bmjopen.bmj.com/site/about/guidelines.xhtml

<u>3. Patient factors and acceptability</u>

Patient receptivity

- “I had no bad experiences at all. People were happy to talk about it.” Practice Nurse, Practice E
- “I was surprised at how receptive the patients were to it.” GP, Practice B

Patient awareness and support for bowel screening

- “They knew pretty much what was involved.” Practice Nurse, Practice E
- My own finding was as soon as you mentioned it to patients and brought up screening, the majority of them were keen to go ahead and do it.” GP, Practice B

Patient priorities and motivation to participate in bowel screening

- “The patient would say, well that’s the least of my concerns and I’ll tell you why…” GP, Practice A
- “Just sort of, inertia and couldn’t be bothered, not a priority.” GP, Practice E

<u>4. Primary care professional factors and acceptability</u>

Acceptability to professionals

- “It isn’t an onerous thing to do and what they have to do is fairly straightforward.” Practice Manager, Practice E
- “It’s just an extension of normal dialogues really.[…] I think it’s entirely appropriate and problem, well almost problem free.[…] It was quick and simple to do and if the feedback turns out to that it’s effective, then I think it would be an appropriate thing to implement in practice.” GP, Practice E

Professional interest, variable support and priority

- “I think there was a bit of a mixed response. I think generally GPs when they’re asked to do something over and above are just like whoa, we’re totally overwhelmed.” GP, Practice A
- “I mean they are so busy here. They are always running late […] so whether or not it just hasn't been a great priority for them.” Practice Nurse, Practice E

Motivation to adopt the intervention

- “Bowel screening is effective and we didn’t have to sell that concept to them. […] I think if they think it’s a good thing they’re more likely to advocate it.” Practice Manager, Practice B
- “Sometimes it felt like quite a positive thing to do because it is about health promotion and disease prevention and that very much chimes with our ethos.” GP, Practice A

The intervention as part of a broader preventive health agenda

- “I think we have to be trying to educate people to look after themselves instead of fixing things after they’re broken.” Practice Nurse, Practice A
- “Giving them a message of empowering them to take control of their destiny, which is something I think that is really lacking in a population like ours.” GP, Practice A

The perceived professional role in educating patients and raising awareness

- “It was a good opportunity to bring it to the forefront of their consciousness […] to kind of put some medical opinion behind it and say, “this is the reason we are doing it”, you know. It does reduce your chances of having a serious bowel cancer if we catch it early.” GP, Practice D
- It was just talking round the practicalities. […] “Oh, I just didn’t know how to do it” […] so you’d try to talk through it a little bit with them.” Practice Nurse, Practice D

Potential for differing roles and involvement

- “I’m a more junior practice nurse, people aren’t coming to me with loads of things, […] so maybe I have more time to look at it.” Practice Nurse, Practice E
- “I do think that the nurses will integrate it more than the GPs will. […] I think GPs deal with the more acute problems, whereas health checks you’ve maybe got a bit more time and people are more relaxed and they are expecting you to ask that.” Practice Manager, Practice A

For peer review only - http://bmjopen.bmj.com/site/about/guidelines.xhtml

Health care context

PCPs reported that certain organisational aspects of primary care services impacted on the implementation of the brief intervention. Existing health promotion interventions already placed demands on practices. PCPs emphasised the highly pressured primary care work environment with a cumulative impact on their ability to commit to new projects.

PCPs also highlighted important barriers to bowel screening participation such as embarrassment and practical issues, in addition to the influence of gender and ethnicity. There was variation by practitioner and also across practices, reflecting PCP's knowledge and belief in screening and also particular patient populations (with reported constraints such as illiteracy and high levels of deprivation).

Processes in delivering the intervention

PCPs commented on the usefulness of the background information on bowel cancer and screening to increase their understanding and belief in screening and the intervention, fostering a sense of commitment. In relation to delivering the intervention itself, staff commented on appropriate types of consultations to raise the topic of screening, appropriate timing within the consultation, frequency of use of the intervention materials, adaptations made, and issues around logging interventions. Staff also referred to use of computer systems to highlight non-responders (also serving as a reminder) and to log and monitor interventions, as well as the need to minimise paperwork and integrate any future interventions into existing computer systems. Constraints raised in ability to deliver the intervention related to difficulty in interacting on the topic of bowel screening with certain groups (e.g. males and minority ethnic groups), but mainly to limited time in an already pressured environment.

Patient factors and acceptability

PCPs reported that patients were positive or neutral but rarely negative when engaging on the topic of screening. They felt that patients were overall receptive to the intervention and discussing bowel screening. In a number of cases, patients had knowledge of bowel cancer and bowel screening and were aware of the benefits of taking part, but there was a large degree of perceived 'inertia', where bowel screening did not appear to be a priority.

For peer review only - http://bmjopen.bmj.com/site/about/guidelines.xhtml

Primary care professional factors and acceptability

The PCPs reported the importance of increasing bowel screening participation and found the process to be acceptable, straightforward and easy to administer as part of routine consultations. However, PCPs reported variation depending on factors such as special interests, personal experience, perceived priorities for the patient population, and forced priorities as a result of limited time and work pressures. Professionals also acknowledged the influence of their attitude towards bowel screening on their approach to the intervention. Those who were motivated drew on the importance of screening and their belief in a holistic approach to health care whereas for others bowel screening was not the highest priority in order to improve patient care.

Interviewees were cognisant of their role in the intervention process in educating patients about bowel cancer and screening and raising awareness of the benefits of participating, and how sometimes this alone was enough to prompt patients to take part. However, they felt they lacked control once the patient had left the consulting room over whether or not they ultimately returned a FOBt kit. There was also discussion of the most appropriate member of the practice team to take the intervention forward, whether this be related to time available, role (GP, PN or HCA), practice load or special interest.

Practice staff reported it was feasible to roll out the intervention and made suggestions for certain adjustments to make it more effective, such as handing out kits directly to patients; streamlining any written materials and making them electronic; integrating any data recording into existing computer systems; and considering funding and set time periods dedicated to bowel screening that complement other initiatives.

End of study evaluation: questionnaires

Nineteen PCPs returned a completed questionnaire (response rate 38.8%). Thirteen were GPs, five were practice nurses and one was a practice manager. This group carried out over half (51.2%) of all interventions (n=132). As reflected in the qualitative interviews, all but one GP (no recorded interventions) stated that most patients were receptive to the intervention; that it could be easily incorporated into practice; and they would theoretically be willing to take part in the study again. Nonetheless, despite positive feedback, ten professionals highlighted lack of time as a potential or actual barrier.

For peer review only - http://bmjopen.bmj.com/site/about/guidelines.xhtml
For peer review only

Four bowel screening staff (out of seven; three screening officers and the screening supervisor) whom had been involved in the intervention returned a completed end of study questionnaire. They all stated that the intervention could be easily incorporated into their workload. However, opinion on the potential impact on workload was uncertain. Three respondents stated that it was difficult identifying calls from patients in intervention practices among over 300 daily calls. However, all four reported that it was suitable for testing in a larger study in its present form.

DISCUSSION

Summary

Results indicate that the intervention was feasible and acceptable to PCPs and patients (as reported by professionals). Of those reached, a small but important minority became responders, a likely underestimate as email and telephone requests were not recorded. It is also possible that some patients may have completed their original kit at home and returned it. The majority of patients approached were willing to discuss the subject of bowel screening. Some patients may have made an informed choice not to participate in screening (indeed informed choice guided the intervention design), although this was not documented in this study.

Qualitative and questionnaire data indicate that the intervention was straightforward and easy to implement and reflected similar ongoing health promotion initiatives, and was thus an effective way to communicate with patients about bowel screening. Overall, PCPs were willing and felt comfortable delivering the intervention in different scenarios, suggesting suitability for most primary care consultations. Practices varied in the number of patients approached and reasons for this variability were widely described in the interviews. Inappropriate or challenging scenarios reported included those involving patients with complex health and social care needs, poor literacy, English as a second language, or sensitivities related to ethnicity and culture. Evidence on appropriate scenarios can help inform future interventions on how to approach these hard to reach groups. Furthermore, these findings have implications for the flexible design of the intervention at a larger scale so it meets the needs of individual practices and different patient groups.

PCPs stated that materials were helpful to promote the intervention, draw attention to it and reinforce messages post-consultation. Nonetheless, not all practices had systems in place to identify non responders,

For peer review only - http://bmjopen.bmj.com/site/about/guidelines.xhtml

or wished to use reminders long-term; both issues can influence the success of any future implementation. Finally, feedback from the SBSC regarding the intervention was also positive, but future implementation would need to take into account the difficulty recoding telephone and email requests and explore all potential mechanisms for requests to be made.

Study strengths and limitations

This was an evidence-based intervention informed by current data on non-participation and psychological theory, grounded in the pragmatic reality of the primary care workload. A good relationship with practices was developed. The study produced a clear audit trail and the duplicate coding of qualitative data helped ensure consistency, rigour and transparency. Nevertheless, this was a small feasibility study which requires further evaluation in larger patient populations. The study also targeted patients who consult in primary care; those who do not consult may present different challenges regarding participation. As we had to adapt to a dynamic, time-pressured primary care environment, it is unknown how many eligible non-responders consulted (and how many of these were approached). Some elements of the intervention were adapted by practices, this is expected in a complex intervention [46]. In fact, an intervention that can be adapted to local circumstances without loss of its essence is a strength that facilitates practical implementation.

The number of patients approached was smaller than our estimates based on national population statistics. This may be due to variation in general practice characteristics and the actual number of non-responders who consulted in primary care. We may have underestimated the number of possible appropriate consultations, although qualitative data suggest that the number of perceived inappropriate scenarios were small. Furthermore, time pressures were described as the main reason for not approaching patients, even among professionals motivated to carry out the intervention. Nonetheless, we consider that an increase in requested bowel screening kits in all recruited practices was a positive outcome influenced by the intervention, and that more patients could be reached over time if the intervention was incorporated into practice, similar to what happens with other types of brief interventions.

Five out of the 11 invited practices completed the study. This is a low participation rate (45.5%), but not uncommon due to recognised challenges in doing research in primary care [47, 48]. Reasons given for non-participation were struggles with demand/targets and pressures in primary care, a challenge also described by

For peer review only - http://bmjopen.bmj.com/site/about/guidelines.xhtml

those who took part in the study. Only one of our recruited practices was within the 30% most deprived areas in Scotland (three were amongst the 50% most deprived). Hence, it is likely that these areas were underrepresented (this would need to be explored in a larger study). To ensure better representation, further intervention roll-out should consider additional strategies to engage with practices in the most deprived areas (such as more personal contact and additional monetary incentives). Nonetheless, it is important to recognise that certain challenges may be beyond the scope of the study, and that priorities in these practices may be different.

Finally, the context of governmental campaigns in Scotland promoting screening participation [49] and the SQoF rewards may have influenced practice decisions to participate in our study. It also made it harder to separate the impact of the campaigns and the intervention on patients' behavioural response.

Comparison with other studies

Brief interventions in primary care are well established and successful in influencing behaviours such as alcohol consumption [50],tobacco smoking [51], and weight management [52]. Our research also contributes to a body of recent UK studies examining primary care-based interventions to influence screening behaviour and demonstrating their effectiveness in improving bowel screening uptake [13, 18, 19], offering further evidence on the benefits of such interventions. Our results show that intervention acceptance varied across practices with the two most deprived practices having the lowest proportion of acceptance and the lowest number of kits requested and returned. This finding suggests that GP endorsement alone is not sufficient to change patient bowel screening behaviour among the most deprived groups, as reflected in a recent study [16].

PCPs reported lack of control after the patient leaves the consulting room - indeed only over a third of those requesting a kit actually returned it - and a gap between intention and action, a phenomenon well described in the literature [53, 54]. The implementation intention plan aimed to help deal with this limitation but was seldom used by PCPs and other studies have shown mixed effectiveness [33, 35, 55-57].

Implications for practice and further research

For peer review only - http://bmjopen.bmj.com/site/about/guidelines.xhtml

Our feasibility parameters did not include a cost-related analysis. Future roll-out should aim to incorporate both direct costs (such as professional time) and indirect costs for the SBSC. These costs need to be balanced against long-term gains in terms of early detection and likely reduction of population mortality [58, 59].

Most interventions were carried out by a GP, which is consistent with national statistics in Scotland demonstrating that GPs carry out about two-thirds of all consultations in primary care [60]. A higher proportion of people seen by a nurse did not accept the intervention. Disease monitoring was a common reason for seeing a nurse, reflecting official data on consultation patterns in Scotland [60]. Interviews show that both GPs and PNs saw their role as important. PNs suggested that they may have more time to deliver interventions incorporated into routine patient checks and reviews, but there was some suggestion that GPs placed greater emphasis on educating and persuading patients. HCAs also reported being in a good position to deliver interventions, though the numbers in this study were small. There is scope to explore further the potential differing roles for members of the primary care team in this context, and to identify ways for different professionals to have a more active involvement.

Practices varied in the number of patients approached. Our qualitative data suggest that the practice population profile, staff's level of engagement with screening, professional knowledge, experience and interests are likely to have been strong explanatory elements. Understanding this variability has implications for the flexible design of the intervention at a larger scale so it meets the needs of individual practices and different patient groups.

The primary care context was described as a highly pressured environment comprising complex patients' needs, limited financial and human resources, increasing patients but diminishing staff, and the need to incentivise health promotion. When asked about the likelihood of continuing on with the intervention, it was clear that despite perceiving it as useful and supporting its underlying ethos, other pressing issues would be prioritised. These challenges constrain the ability to deliver and sustain the intervention, irrespective of motivation, willingness and recognised importance. However, the flexibility of the intervention meant that it could be adapted to suit individual practices and demonstrated an impact on bowel screening participation despite the outlined constraints.

For peer review only - http://bmjopen.bmj.com/site/about/guidelines.xhtml

For peer review only

CONCLUSIONS

We tested a primary care-based intervention to increase uptake in non-responders to FOBt screening, and found it to be feasible and acceptable in Scottish primary practices, despite recognised organisational and system constraints that would need to be considered for the intervention to be more widely implemented. Further testing in a randomised controlled trial would give robust evidence of the effectiveness of the brief intervention in increasing informed screening participation. The intervention can be useful as one tool to complement other efforts to engage with non-responders and reflects the broader aims from the Scottish government to raise awareness and normalise bowel screening. Our study adds to evidence that primary care can play a key role in promoting bowel screening uptake.

AUTHORS' CONTRIBUTIONS

All authors designed the study. CC, DW, DC and NC were involved in recruitment, data collection and data analysis. All authors contributed to the drafting of the manuscript and the approval of the final version.

FUNDING STATEMENT

This work was supported by CRUK [grant reference number C12357/A13965] through the National Awareness and Early Diagnosis Initiative (NAEDI-2). The funder had no role in the design, collection, analysis, and interpretation of data; in the writing of the manuscript; and in the decision to submit the manuscript for publication.

CONFLICT OF INTEREST STATEMENT

The authors declare no conflicts of interest.

DATA SHARING STATEMENT

Some of the unpublished data are available from the authors (such as intervention materials and questionnaires). The corresponding author (natalia.calanzani@ed.ac.uk) can be contacted by anyone interested in accessing these. Data from patients, primary care practices and the Scottish Bowel Screening Centre cannot be accessed by anyone who is not part of the research team due to ethical and confidentiality concerns.

ACKNOWLEDGEMENTS

For peer review only - http://bmjopen.bmj.com/site/about/guidelines.xhtml

For peer review only

Bowel screening data were obtained from the Scottish Bowel Screening Centre in Dundee; we thank all staff for their assistance and for completing the end of study questionnaires. We acknowledge the support and advice from NHS Lothian, which provided the practice lists used for recruiting practices and the help from NHS Scotland which provided the leaflets in Polish. We thank Dr Robert Scully for his help with medical acronyms/terms in the intervention proformas and Dr Ewan Gray for his advice when revising this manuscript. We also thank the reviewers for their comments and recommendations. We are very grateful to each GP, practice nurse, health care assistant and practice manager who agreed to take part in the study, gave up their time to be interviewed and completed the end of study questionnaires.

REFERENCES

[1] Damery S, Smith S, Clements A, et al. Evaluating the effectiveness of GP endorsement on increasing participation in the NHS Bowel Cancer Screening Programme in England: study protocol for a randomized controlled trial. *Trials* 2012;13:18.

[2] Hewitson P, Glasziou Paul P, et al. Screening for colorectal cancer using the faecal occult blood test, Hemoccult. *Cochrane Database Syst Rev*: John Wiley & Sons, Ltd, 2007.

[3] Bowel Cancer UK. Screening in the Nations. 2015. Available from: http://www.bowelcanceruk.org.uk/understanding-bowel-cancer/screening/screening-in-the-uk/.

[4] Scottish Bowel Screening Programme. Scottish Bowel Screening Programme. Key Performance Indicators Report: May 2015 data submission. Invitations between 1st November 2012 and 31st October 2014. https://www.isdscotland.org/Health-Topics/Cancer/Publications/2015-08-04/2015-08-04-Bowel-Screening-KPI-Full-Report.xlsx, 2015

[5] European Commission. European guidelines for quality assurance in colorectal cancer screening and diagnosis. Luxembourg: Publications Office of the European Union, 2010.

[6] Holden DJ, Harris R, Porterfield DS, et al. Enhancing the use and quality of colorectal cancer screening. *Evid Rep Technol Assess* 2010:1-195.

[7] Jepson R, Clegg A, Forbes C, et al. The determinants of screening uptake and interventions for increasing uptake: a systematic review. *Health Technol Assess* 2000;4:i-vii,1-133.

[8] Palmer CK, Thomas MC, von Wagner C, et al. Reasons for non-uptake and subsequent participation in the NHS Bowel Cancer Screening Programme: a qualitative study. *Br J Cancer* 2014;110:1705-11.

For peer review only - http://bmjopen.bmj.com/site/about/guidelines.xhtml

[9] Chapple A, Ziebland S, Hewitson P, et al. What affects the uptake of screening for bowel cancer using a faecal occult blood test (FOBt): a qualitative study. *Soc Sci Med* 2008;66:2425-35.

[10] Power E, Miles A, von Wagner C, et al. Uptake of colorectal cancer screening: system, provider and individual factors and strategies to improve participation. *Future Oncol* 2009;5:1371-88.

[11] Malila N, Senore C, Armaroli P. European guidelines for quality assurance in colorectal cancer screening and diagnosis. First Edition--Organisation. *Endoscopy* 2012;44 Suppl 3:Se31-48.

[12] Powe BD, Faulkenberry R, Harmond L. A review of intervention studies that seek to increase colorectal cancer screening among African-Americans. *Am J Health Promot* 2010;25:92-9.

[13] Wardle J, von Wagner C, Kralj-Hans I, et al. Effects of evidence-based strategies to reduce the socioeconomic gradient of uptake in the English NHS Bowel Cancer Screening Programme (ASCEND): four cluster-randomised controlled trials. *Lancet* 2015;387:751-59.

[14] Brouwers MC, De Vito C, Bahirathan L, et al. Effective interventions to facilitate the uptake of breast, cervical and colorectal cancer screening: an implementation guideline. *Implement Sci* 2011;6:112.

[15] Sabatino SA, Lawrence B, Elder R, et al. Effectiveness of interventions to increase screening for breast, cervical, and colorectal cancers: nine updated systematic reviews for the guide to community preventive services. *Am J Prev Med* 2012;43:97-118.

[16] Raine R, Duffy SW, Wardle J, et al. W. Impact of general practice endorsement on the social gradient in uptake in bowel cancer screening. *Br J Cancer* 2016;114:321-6.

[17] Rubin G, Berendsen A, Crawford SM, et al. The expanding role of primary care in cancer control. *Lancet Oncol* 2015;16:1231-72.

[18] Hewitson P, Ward AM, Heneghan C, et al. D. Primary care endorsement letter and a patient leaflet to improve participation in colorectal cancer screening: results of a factorial randomised trial. *Br J Cancer* 2011;105:475-80.

[19] Shankleman J, Massat NJ, Khagram L, et al. Evaluation of a service intervention to improve awareness and uptake of bowel cancer screening in ethnically-diverse areas. *Br J Cancer* 2014;111:1440-7.

[20] The Scottish Government. Detect Cancer Early. 2015. http://www.gov.scot/Topics/Health/Services/Cancer/Detect-Cancer-Early

[21] ISD Scotland, National Services Scotland. Quality and Outcomes Framework. Prevalence, achievement, payment and exceptions data for Scotland, 2013/2014. https://isdscotland.scot.nhs.uk/Health-Topics/General-Practice/Publications/2014-09-30/2014-09-30-QOF-Report.pdf?44355410338, 2014

For peer review only - http://bmjopen.bmj.com/site/about/guidelines.xhtml

[22] The Scottish Government. Detect Cancer Early - Bowel Screening Initiative. Health and Social Care Integration Directorate Primary Care Division, 2013.

[23] The Scottish Government. Scottish Index of Multiple Deprivation 2012. 2012. http://simd.scotland.gov.uk/publication-2012/.

[24] Department for Constitutional Affairs. Mental Capacity Act 2005 Code of Practice. London, 2007. https://www.gov.uk/government/uploads/system/uploads/attachment_data/file/497253/Mental-capacity-act-code-of-practice.pdf.

[25] Gollwitzer PM. Goal Achievement: The Role of Intentions. *Eur Rev Soc Psychol* 1993;4:141-85.

[26] Campbell C, Techer L, Crawford F, et al Primary Care based strategies to increase uptake of colorectal cancer screening. USA: Report to the National Cancer Institute, 2009.

[27] Bastani R, Glenn BA, Taylor VM, et al. Integrating theory into community interventions to reduce liver cancer disparities: The Health Behavior Framework. *Prev Med* 2010;50:63-7.

[28] Treasure J. Motivational interviewing. *Adv Psychiatr Treat* 2004;10:331-7.

[29] Jepson RG, Hewison J, Thompson A, et al. Patient perspectives on information and choice in cancer screening: a qualitative study in the UK. *Soc Sci Med* 2007;65:890-9.

[30] Tu SP, Yip MP, Chun A, et al. Development of intervention materials for individuals with limited English proficiency: lessons learned from "Colorectal Cancer Screening in Chinese Americans". *Med Care* 2008; 46:S51-61.

[31] Jo AM, Maxwell AE, Wong WK, et al. Colorectal cancer screening among underserved Korean Americans in Los Angeles County. *Journal Of Immigrant And Minority Health* 2008; 10:119-26.

[32] Maxwell AE, Bastani R, Crespi CM, et al. Behavioral mediators of colorectal cancer screening in a randomized controlled intervention trial. *Prev Med* 2011; 52:167-73.

[33] Sheeran P, Orbell S. Using implementation intentions to increase attendance for cervical cancer screening. *Health Psychol* 2000; 19:283-9.

[34] Rutter D, Quine L, Steadman L, et al. Increasing Attendance at Breast Cancer Screening: Field Trial. Final Report to NHSBSP. University of Kent. Department of Psychology. Centre for Research in Health Behaviour, 2007.

[35] Greiner KA, Daley CM, Epp A, et al. Implementation Intentions and Colorectal Screening: A Randomized Trial in Safety-Net Clinics. *Am J Prev Med* 2014; 47:703-14.

For peer review only

For peer review only - http://bmjopen.bmj.com/site/about/guidelines.xhtml

[36] Ling BS, Schoen RE, Trauth JM, et al. Physicians encouraging colorectal screening: a randomized controlled trial of enhanced office and patient management on compliance with colorectal cancer screening. *Arch Intern Med* 2009; 169:47-55.

[37] Lasser KE, Murillo J, Medlin E, et al. A multilevel intervention to promote colorectal cancer screening among community health center patients: results of a pilot study. *BMC Family Practice* 2009; 10:1-7.

[38] NHS Inform. Risks and benefits of bowel screening. 2013. Available from: . https://www.nhsinform.scot/healthy-living/screening/bowel-screening/introduction-to-bowel-screening#benefits-and-risks

[39] NHS Cancer Screening Programmes. Consent to Cancer Screening. Cancer Screening Series No 4., 2008.

[40] IBM SPSS Statistics for Windows, Version 19.0. Armonk, NY: IBM Corp.: IBM Corp, 2010.

[41] Baker R, Brick MJ, Bates NA, Battaglia M, Couper MP, Dever JA, K.J. G, Tourangeau R. Report of the AAPOR Task Force on Non-Probability Sampling. 2013. Available from: https://www.aapor.org/AAPOR_Main/media/MainSiteFiles/NPS_TF_Report_Final_7_revised_FNL_6_22_13.pdf

[42] Gale N, Heath G, Cameron E, et al. Using the framework method for the analysis of qualitative data in multi-disciplinary health research. *BMC Med Res Methodol* 2013; 13:117.

[43] Ritchie J, Spencer L. Qualitative data analysis for applied policy research. In: Bryman A, Burgess R, editors. Analyzing qualitative data. Abindgon: Routledge, 1994:173-94.

[44] NVivo qualitative data analysis Software. QSR International Pty Ltd, 2006.

[45] Krippendorf K. Content analysis: An introduction to its methodology. Beverly Hills, CA: Sage Publications, 1980.

[46] Medical Research Council. Developing and evaluating complex interventions: new guidance. Available from: http://www.mrc.ac.uk/documents/pdf/complex-interventions-guidance/, 2008.

[47] Baird B, Charles A, Honeyman M, et al. Understanding pressures in general practice. The King's Fund, 2016:1-100.

[48] Wilson S, Delaney BC, Roalfe A, et al. Randomised controlled trials in primary care: case study. *BMJ* 2000; 321:24.

[49] The Scottish Government. New bowel cancer home screening test. 2015. http://news.gov.scot/news/new-bowel-cancer-home-screening-test#downloads

For peer review only - http://bmjopen.bmj.com/site/about/guidelines.xhtml

[50] Kaner EF, Beyer F, Dickinson HO, et al. Effectiveness of brief alcohol interventions in primary care populations. *Cochrane Database Syst Rev* 2007:Cd004148.

[51] Bauld L, Bell K, McCullough L, et al. The effectiveness of NHS smoking cessation services: a systematic review. *J Public Health* 2010; 32:71-82.

[52] Aveyard P, Lewis A, Tearne S, et al. Screening and brief intervention for obesity in primary care: a parallel, two-arm, randomised trial. *Lancet* 2016;388:2492–500.

[53] Sheeran P. Intention—Behavior Relations: A Conceptual and Empirical Review. *Eur Rev Soc Psychol* 2002; 12:1-36.

[54] Power E, Van Jaarsveld CH, McCaffery K, et al. Understanding intentions and action in colorectal cancer screening. *Ann Behav Med* 2008; 35:285-94.

[55] Rutter D, Quine L, Steadman L, et al. Increasing Attendance at Breast Cancer Screening: Field Trial. Final Report to NHSBSP. University of Kent. Department of Psychology. Centre for Research in Health Behaviour, 2007.

[56] Lo SH, Good A, Sheeran P, et al. Preformulated implementation intentions to promote colorectal cancer screening: a cluster-randomized trial. *Health Psychol* 2014; 33:998-1002.

[57] Walsh JC. Increasing Screening Uptake for a Cervical Smear Test: Predictors of Attendance and the use of Action Plans in Prior Non-Attenders. *The Irish Journal of Psychology* 2005; 26:65-73.

[58] Sharp L, Tilson L, Whyte S, et al. Cost-effectiveness of population-based screening for colorectal cancer: a comparison of guaiac-based faecal occult blood testing, faecal immunochemical testing and flexible sigmoidoscopy. *Br J Cancer* 2012; 106:805-16.

[59] Birtwistle M, Earnshaw A. Saving lives, averting costs. An analysis of the financial implications of achieving earlier diagnosis of colorectal, lung and ovarian cancer Incisive Health on behalf of Cancer Research UK, 2014.

[60] ISD Scotland. Practice Team Information (PTI) Annual Update (2012/13). Available from: http://www.isdscotland.org/Health-Topics/General-Practice/Publications/2013-10-29/2013-10-29-PTI-Report.pdf, 2013.

For peer review only

For peer review only - http://bmjopen.bmj.com/site/about/guidelines.xhtml

Supplementary file 1:

A. **Estimating the size of the study population**
B. **Intervention supporting materials**
C. **Feasibility parameters**

A. Estimating the size of the study population

According to the 2011 Census, there are 184,846 residents in Lothian aged 50-74[1] (the age range for eligibility to bowel cancer screening). In these age groups, approximately 86% of patients (estimated 158,968) consult at least once a year (aged 45 and over are included as data were not available separately)[2], with a linear increase in the number of consultations according to patients' age (from 5.2 annual consultations to 7.7 among the most deprived groups).
As of September 2013 there were 127 practices in Lothian[3]. Each practice would then have, on average, at least 1252 patients consulting during our intervention period (although numbers vary between practices).

Official Scottish Bowel screening data for the period 2011-2013 show that uptake in Lothian was 55.3% (ranging from 39.6% to 59.8% from the most deprived to the least deprived areas)[4]. Considering the participation rates for the most deprived areas (39.6%) and the population aged 50-74; there would roughly be a maximum of 756 eligible patients who could be reached during the study period per practice, or 4536 in six selected practices.

In practice, however, numbers are likely to be much smaller. For example, in 2011 the prevalence of cancer (all types) in Scotland was 4.5%[5]. These patients would not take part in the intervention as it would not be appropriate to approach the issue of cancer screening to someone in receipt of cancer treatment. Considering cancer prevalence in Scotland, the number of eligible patients would be reduced to 4355 in all practices. This is a conservative estimate as cancer is more common in areas of high deprivation (which is the case of some of the selected practices in Lothian). Furthermore, the opportunity to approach a patient about screening may not arise in every consultation. We estimate that an opportunity will arise in about a quarter of all consultations with eligible participants, thus reducing the number of patients to 1089 (or approximately 182 per practice).

References:

1.Scotland's Census 2011 - National Records of Scotland. Table DC1117SC - Age by sex. All people [Internet]. 2014. Available from: http://www.scotlandscensus.gov.uk.

2.Estimated number of patients seen1/not seen2 in Scotland by either a General Practitioner (GP) or Practice Nurse [Internet]. 2013. Available from: http://www.isdscotland.org/PTI/.

3 .General Practice – GP workforce and practice population statistics to 2013. Scottish general practice characteristics: as at 30th September from 2005 to 2013. In: ISD Scotland, editor. 2014.

4.Scottish Bowel Screening Programme. Scottish Bowel Screening Programme. Key Performance Indicators Report: May 2014 data submission. Invitations between 1st November 2011 and 31st October 2013. Scotland: Scottish Bowel Screening Programme; 2014.

5. ISD Scotland. Cancer in Scotland: ISD Scotland; 2012. Available from: https://isdscotland.scot.nhs.uk/Health-Topics/Cancer/Publications/2012-04-24/Cancer_in_Scotland_summary_m.pdf

For peer review only

B. Intervention supporting materials

Intervention material	Aim(s)	Theoretical framework/adopted principles
3-4 questions/statements<br>• A5 coloured laminated sheet of paper<br>• Given to the health care professional	• To guide discussion addressing concerns around or barriers to bowel screening with non-responders	• Non-directive statements<br>• Non-coercion<br>• Informed choice<br>• Implementation Intentions theory
Staff flowchart<br>• A4 coloured laminated sheet of paper<br>• Given to the health care professional	• To describe barriers to screening (with examples), and evidence-based approaches to deal with these barriers	• Health Behaviour Framework<br>• Motivational Interviewing
Patient leaflet and freepost envelope<br>• A4 coloured sheet of paper folded into A5, perforated so a reply slip could be sent back by post<br>• FREEPOST envelope addressed to the bowel screening centre<br>• Given to patients who accepted the intervention	• To deconstruct health beliefs associated with low uptake, i.e. the barriers described in the flowchart<br>• To provide space for the patient to develop their own implementation plan<br>• To offer the opportunity to request a new test kit	• Health Behaviour Framework<br>• Non-coercion<br>• Informed choice<br>• Implementation Intentions theory
Guidance sheet<br>• A4 coloured laminated sheet of paper<br>• Given to the health care professional	• To provide practice staff with relevant information regarding the research study	N/A
Practice Proforma<br>• Green A4 sheet with perforations (3 proformas in each)<br>• Given to the health care professional<br>• Designed to require approximately 2 minutes to be completed	• To collect relevant intervention data so the feasibility outcomes could be assessed:<br>○ Intervention date, staff name and role, duration of the intervention, patient age and gender, reasons for consultation (text)<br>○ Whether intervention was accepted, leaflet was given and completed in practice<br>○ Comments	N/A

Note: Bowel Screening leaflets in Polish were requested from NHS Scotland and provided to practices with a larger Polish population

For peer review only

C. Feasibility parameters

- Processes required in general practice to record (and flag) responder status in patients' electronic records
- Number of patients approached during consultations
- Number of interventions delivered over the intervention period
- Length of time the intervention takes
- Number and willingness of patients to engage in conversation when responder status is raised during a consultation
- Number of patients who were willing to receive leaflet
- Number of patients who completed leaflet with health care professional
- Number of patients who sent reply slip
- Number of patients who returned a completed kit
- Processes required in the bowel screening centre to deal with the extra-to-programme returned FOBt kits, and to ensure over-screening does not occur
- Whether the brief intervention leads to longer discussion with patients regarding cancer screening, or bowel symptoms
- Willingness of GPs and practice nurses to deliver intervention
- Overall views on the study's feasibility according to primary care professionals
- Overall views on the study's feasibility according to bowel screening staff

For peer review only

Supplementary file 2

A. Recruitment Flowchart

B. Characteristics of practices approached in Wave 1

A. Recruitment Flowchart

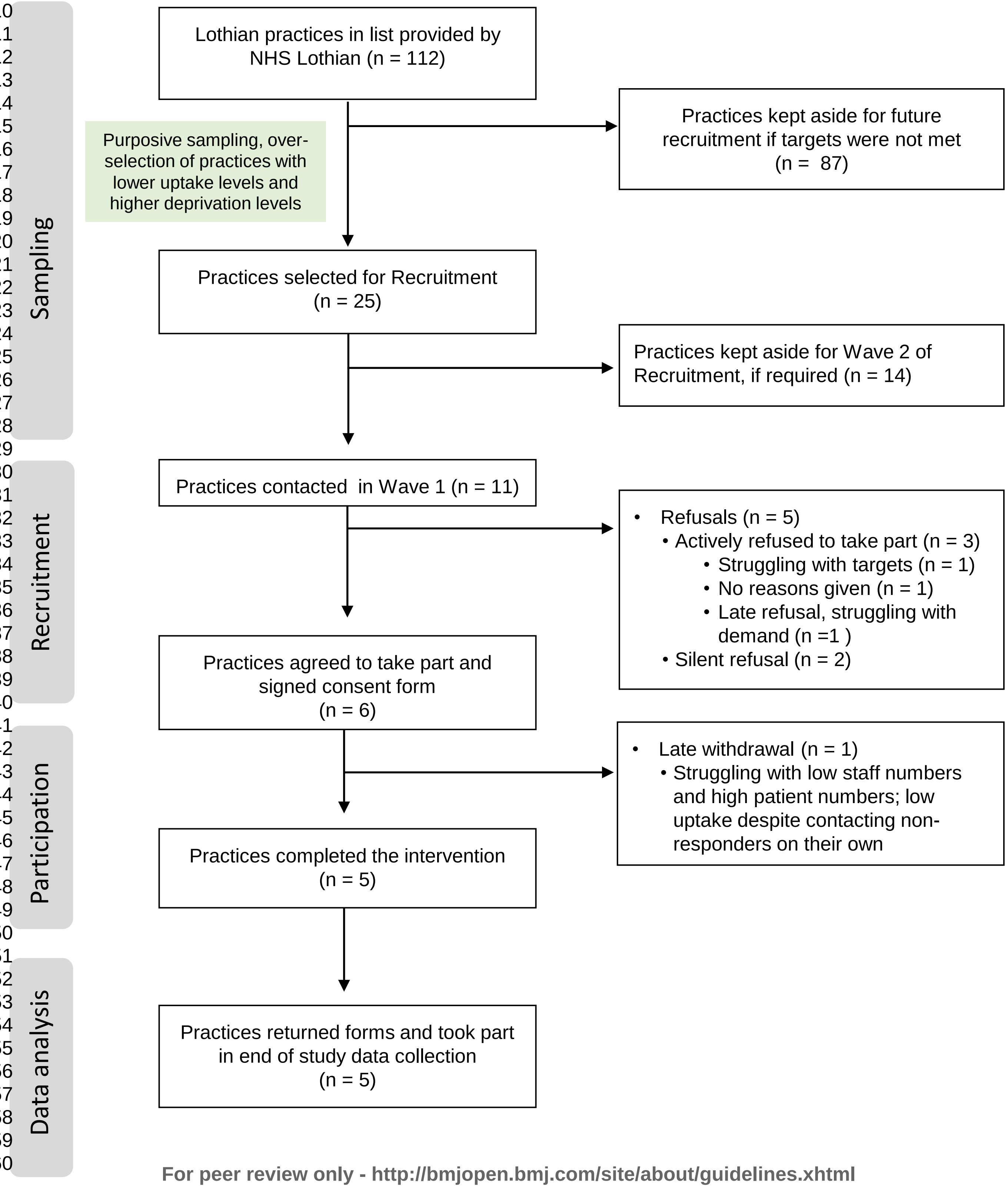

B. Characteristics of practices approached in Wave 1

Practice name	Outcome in Wave 1 of recruitment	Uptake % (2013)	Pop 50-75	Mean SIMD decile* (50-75yr olds)	Average Monthly Non Responder Numbers	Practice list size (ISD)
Practice A	Recruited	<45%	1,413	2.6	41	6,888
Practice B	Recruited	45-50%	2,654	3.6	61	10,440
Practice C	Recruited	50-55%	2,515	4.5	56	8,693
Practice D	Recruited	50-55%	1,241	6.2	29	5,326
Practice E	Recruited	55-60%	1,668	5.5	30	5,201
Practice F	Recruited, but withdrew as struggling with pressures	50-55%	3,444	3.2	73	11,624
Practice G	Actively refused, struggling with pressures	<45%	2,543	2.1	70	12,482
Practice H	Actively refused, struggling with pressures	45-50%	2,030	4.2	47	9,585
Practice I	Actively refused, no reasons given	50-55%	3,809	4.6	76	13,984
Practice J	Silent refusal	<45%	1,075	3.9	29	7,848
Practice K	Silent refusal	45-50%	2,241	3.4	50	8,287

Abbreviations: ISD: Information Services Division; Pop: population; SIMD: Scottish Index of Multiple Deprivation.
*The lower the decile number, the higher the deprivation levels.

Supplementary file 3:

A. **Recorded reasons for consultation**
B. **Comparing patients who accepted and did not accept the intervention**

A. Recoded reasons for consultation

Reasons for consultation	Practice A n(%)	Practice B n(%)	Practice C n(%)	Practice D n(%)	Practice E n(%)	All practices n(%)			
	All	All	All	All	All	GP	PN	HCA	All
Known chronical illness/review of existing condition	25 (36.8)	6 (11.5)	2 (7.4)	18 (26.1)	4 (11.8)	12 (21.8)	40 (72.7)	3 (5.5)	55 (22.0)
Musculoskeletal symptoms/conditions	3 (4.4)	7 (13.5)	7 (25.9)	11 (15.9)	5 (14.7)	33 (100.0)	0 (0.0)	0 (0.0)	33 (13.2)
Tests/test results	8 (11.8)	8 (15.4)	3 (11.1)	8 (11.6)	2 (5.9)	4 (13.8)	17 (58.6)	8 (27.6)	29 (11.6)
Respiratory or ENT symptoms/conditions	6 (8.8)	7 (13.5)	2 (7.4)	4 (5.8)	3 (8.8)	14 (63.6)	8 (36.4)	0 (0.0)	22 (8.8)
Prescriptions/medication review	7 (10.3)	2 (3.8)	1 (3.7)	1 (1.4)	4 (11.8)	15 (100.0)	0 (0.0)	0 (0.0)	15 (6.0)
Skin complaints	1 (1.5)	5 (9.6)	2 (7.4)	3 (4.3)	4 (11.8)	13 (86.7)	2 (13.3)	0 (0.0)	15 (6.0)
Non-clinical	2 (2.9)	4 (7.7)	2 (7.4)	5 (7.2)	1 (2.9)	10 (71.4)	2 (14.3)	2 (14.3)	14 (5.6)
Gastrointestinal symptoms/conditions	3 (4.4)	4 (7.7)	1 (3.7)	4 (5.8)	2 (5.9)	14 (100.0)	0 (0.0)	0 (0.0)	14 (5.6)
Multiple issues	4 (5.9)	1 (1.9)	0 (0.0)	1 (1.4)	5 (14.7)	11 (100.0)	0 (0.0)	0 (0.0)	11 (4.4)
Mental health symptoms/conditions	0 (0.0)	2 (3.8)	2 (7.4)	2 (2.9)	1 (2.9)	7 (100.0)	0 (0.0)	0 (0.0)	7 (2.8)
Preventative behaviour	4 (5.9)	1 (1.9)	2 (7.4)	0 (0.0)	0 (0.0)	0 (0.0)	7 (100.0)	0 (0.0)	7 (2.8)
Gynaecological or urological symptoms/conditions	2 (2.9)	1 (1.9)	0 (0.0)	1 (1.4)	0 (0.0)	4 (100.0)	0 (0.0)	0 (0.0)	4 (1.6)
Inconclusive	0 (0.0)	0 (0.0)	0 (0.0)	2 (2.9)	0 (0.0)	2 (100.0)	0 (0.0)	0 (0.0)	2 (0.8)
Other/non-specific symptoms	3 (4.4)	4 (7.7)	3 (11.1)	9 (13.0)	3 (8.8)	21 (95.5)	1 (4.5)	0 (0.0)	22 (8.8)
Total	68 (100.0)	52 (100.0)	27 (100.0)	69 (100.0)	34 (100.0)	N/A	N/A	N/A	250 (100.0)

Abbreviations: GP: General Practitioner; PN: Practice Nurse; HCA – Health Care Assistant; ENT – ear, nose and throat
Missing data: 8 cases (3 in Practice A, 1 in Practice C, 1 in Practice D and 3 in Practice B).
Sums may not add up to 100 due to rounding

For peer review only

B. Comparing patients who accepted and did not accept the intervention

Overall data	Accepted the intervention (n=220) n(%)	Did not accept the intervention (n=33) n(%)	Overall (n=253)* n(%)
**Patient sex**			
Male	113 (51.4)	22 (66.7)	135 (53.4)
Female	107 (48.6)	11 (33.3)	118 (46.6)
**Patient age**			
Median (IQR)	58.00 (53.00-64.75)	64.00 (57.00-71.50)	58.00 (53.00-65.00)
50-54	78 (35.5)	3 (9.1)	81 (32.5)
55-59	48 (21.8)	9 (27.3)	57 (22.9)
60-64	36 (16.4)	6 (18.2)	42 (16.9)
65-69	32 (14.5)	3 (9.1)	35 (14.1)
70-74	17 (7.7)	9 (27.3)	26 (10.4)
75-79	5 (2.3)	3 (9.1)	8 (3.2)
**Staff carrying out the intervention**			
GP	153 (69.5)	11 (33.3)	164 (64.8)
PN	57 (25.9)	19 (57.6)	76 (30.0)
HCA	10 (4.5)	3 (9.1)	13 (5.1)
**Duration of the intervention**			
Median (IQR)	2.00 (1.00-5.00)	2.00 (2.00-5.00)	2.00 (1.00-5.00)
**Reasons for consultation**			
Known chronical illness/review of existing condition	42 (19.7)	12 (37.5)	54 (22.0)
Musculoskeletal symptoms/conditions	30 (14.1)	3 (9.4)	33 (13.5)
Tests/test results	22 (10.3)	5 (15.6)	27 (11.0)
Respiratory or ENT symptoms/conditions	17 (8.0)	5 (15.6)	22 (9.0)
Prescriptions/medication review	15 (7.0)	0 (0.0)	15 (6.1)
Skin complaints	15 (7.0)	0 (0.0)	15 (6.1)
Non-clinical	14 (6.6)	0 (0.0)	14 (5.7)
Gastrointestinal symptoms/conditions	12 (5.6)	1 (3.1)	13 (5.3)
Multiple issues	10 (4.7)	1 (3.1)	11 (4.5)
Mental health symptoms/conditions	6 (2.8)	1 (3.1)	7 (2.9)
Preventative behaviour	5 (2.3)	1 (3.1)	6 (2.4)
Gynaecological or urological symptoms/conditions	2 (0.9)	2 (6.3)	4 (1.6)
Inconclusive	2 (0.9)	0 (0.0)	2 (0.8)
Other/non-specific symptoms	21 (9.9)	1 (3.1)	22 (9.0)

Abbreviations: GP: General Practitioner; PN: Practice Nurse; HCA – Health Care Assistant; ENT – ear, nose and throat; IQR – Interquartile range
*In 5 cases data were missing on whether patient accepted the intervention. These cases are not included here; hence the overall values do not match the ones in the main manuscript (i.e. 258 participants). There were also missing data for patient age (4 cases), duration of intervention (9 cases) and reasons for consultation (8 cases). Sums may not add up to 100 due to rounding.

For peer review only

Supplementary file 4

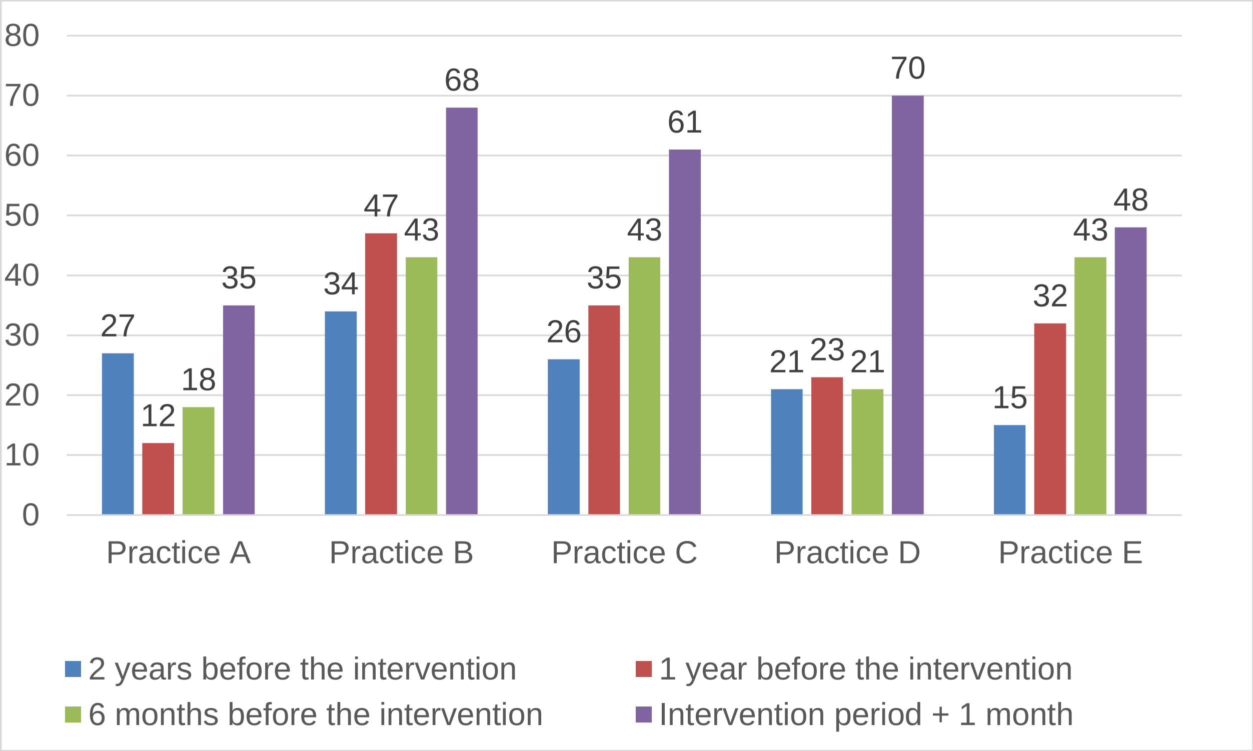

For peer review only

STROBE 2007 (v4) Statement—Checklist of items that should be included in reports of *cross-sectional studies*

Section/Topic	Item #	Recommendation	Reported on page #
**Title and abstract**	1	(*a*) Indicate the study's design with a commonly used term in the title or the abstract	1
		(*b*) Provide in the abstract an informative and balanced summary of what was done and what was found	2
**Introduction**			
Background/rationale	2	Explain the scientific background and rationale for the investigation being reported	3,4
Objectives	3	State specific objectives, including any prespecified hypotheses	4
**Methods**			
Study design	4	Present key elements of study design early in the paper	5,6
Setting	5	Describe the setting, locations, and relevant dates, including periods of recruitment, exposure, follow-up, and data collection	4,5,8
Participants	6	(*a*) Give the eligibility criteria, and the sources and methods of selection of participants	4,5
Variables	7	Clearly define all outcomes, exposures, predictors, potential confounders, and effect modifiers. Give diagnostic criteria, if applicable	6
Data sources/ measurement	8*	For each variable of interest, give sources of data and details of methods of assessment (measurement). Describe comparability of assessment methods if there is more than one group	7
Bias	9	Describe any efforts to address potential sources of bias	Limitations and potential sources of bias approached in the discussion (page 17)
Study size	10	Explain how the study size was arrived at	4,5
Quantitative variables	11	Explain how quantitative variables were handled in the analyses. If applicable, describe which groupings were chosen and why	7
Statistical methods	12	(*a*) Describe all statistical methods, including those used to control for confounding	7
		(*b*) Describe any methods used to examine subgroups and interactions	N/A
		(*c*) Explain how missing data were addressed	Reported in all cases (table footnotes on pages 9 and 10, supplementary file 3). N/A for multivariate analysis as only descriptive statistics were reported
		(*d*) If applicable, describe analytical methods taking account of sampling strategy	N/A

For peer review only

		(*e*) Describe any sensitivity analyses	N/A
**Results**			
Participants	13*	(a) Report numbers of individuals at each stage of study—eg numbers potentially eligible, examined for eligibility, confirmed eligible, included in the study, completing follow-up, and analysed	8, 9, 10, 11 and supplementary file 3
		(b) Give reasons for non-participation at each stage	Not available, limitations approached on pages 17-19
		(c) Consider use of a flow diagram	Supplementary file 2
Descriptive data	14*	(a) Give characteristics of study participants (eg demographic, clinical, social) and information on exposures and potential confounders	8, 9, 10, 11, supplementary file 3, limitations discussed in pages 17-19
		(b) Indicate number of participants with missing data for each variable of interest	Reported in all cases (table footnotes on pages 9 and 10, supplementary file 3).
Outcome data	15*	Report numbers of outcome events or summary measures	8, 9, 10, 11
Main results	16	(*a*) Give unadjusted estimates and, if applicable, confounder-adjusted estimates and their precision (eg, 95% confidence interval). Make clear which confounders were adjusted for and why they were included	N/A, no multivariate analyses were carried out
		(*b*) Report category boundaries when continuous variables were categorized	N/A
		(*c*) If relevant, consider translating estimates of relative risk into absolute risk for a meaningful time period	N/A
Other analyses	17	Report other analyses done—eg analyses of subgroups and interactions, and sensitivity analyses	Supplementary file 3
**Discussion**			
Key results	18	Summarise key results with reference to study objectives	16
Limitations	19	Discuss limitations of the study, taking into account sources of potential bias or imprecision. Discuss both direction and magnitude of any potential bias	17-19
Interpretation	20	Give a cautious overall interpretation of results considering objectives, limitations, multiplicity of analyses, results from similar studies, and other relevant evidence	16-19
Generalisability	21	Discuss the generalisability (external validity) of the study results	17-19
**Other information**			
Funding	22	Give the source of funding and the role of the funders for the present study and, if applicable, for the original study on which the present article is based	20

*Give information separately for cases and controls in case-control studies and, if applicable, for exposed and unexposed groups in cohort and cross-sectional studies.

Note: An Explanation and Elaboration article discusses each checklist item and gives methodological background and published examples of transparent reporting. The STROBE checklist is best used in conjunction with this article (freely available on the Web sites of PLoS Medicine at http://www.plosmedicine.org/, Annals of Internal Medicine at http://www.annals.org/, and Epidemiology at http://www.epidem.com/). Information on the STROBE Initiative is available at www.strobe-statement.org.

For peer review only

BMJ Open

Is an opportunistic primary care-based intervention for non-responders to bowel screening feasible and acceptable? A mixed methods feasibility study in Scotland

Journal:	*BMJ Open*
Manuscript ID	bmjopen-2017-016307.R2
Article Type:	Research
Date Submitted by the Author:	06-Jul-2017
Complete List of Authors:	Calanzani, Natalia ; University of Edinburgh, Centre For Population Health Sciences, The Usher Institute of Population Health Sciences and Informatics<br>Cavers, Debbie; University of Edinburgh, General Practice<br>Vojt, Gabriele; Glasgow Caledonian University, Department of Psychology<br>Orbell, Sheina; University of Essex, Department of Psychology<br>Steele, Robert; University of Dundee, Surgery and Molecular Oncology<br>Brownlee, Linda; Scottish Bowel Screening Centre<br>Smith, Steve; University Hospitals of Coventry & Warwickshire NHS Trust, Midlands & NW Bowel Cancer Screening Hub, Hospital of St. Cross<br>Patnick, Julietta; University of Oxford, Cancer Epidemiology Unit<br>Weller, David; University of Edinburgh, General Practice<br>Campbell, Christine; University of Edinburgh, Centre for Population Health Sciences
**Primary Subject Heading**:	Health services research
Secondary Subject Heading:	General practice / Family practice, Public health, Oncology
Keywords:	feasibility studies, bowel screening, general practice, neoplasms

SCHOLARONE™
Manuscripts

Is an opportunistic primary care-based intervention for non-responders to bowel screening feasible and acceptable? A mixed methods feasibility study in Scotland

Natalia Calanzani[1], Debbie Cavers[1], Gabriele Vojt[2], Sheina Orbell[3], Robert JC Steele[4], Linda Brownlee[5], Steve Smith[6], Julietta Patnick[7], David Weller[1], Christine Campbell[1]

[1]University of Edinburgh, The Usher Institute of Population Health Sciences and Informatics, Centre for Population Health Sciences, Medical School, Teviot Place, Edinburgh EH8 9AG, UK

[2]Glasgow Caledonian University, Department of Psychology, Cowcaddens Road, Glasgow G4 0BA

[3]Department of Psychology, University of Essex, Wivenhoe Park, Colchester CO4 3SQ, UK

[4]Surgery and Molecular Oncology, Ninewells Hospital, University of Dundee, Dundee, DD1 9SY, Scotland, UK

[5]Scottish Bowel Screening Centre, Kings Cross, Clepington Road, Dundee, DD3 8EA, Scotland, UK

[6]University Hospitals of Coventry & Warwickshire NHS Trust, Midlands & NW Bowel Cancer Screening Hub, Hospital of St. Cross, Barry Road, Rugby, CV22 5PX

[7]Oxford University, Cancer Epidemiology Unit, Richard Doll Building, Roosevelt Drive, Oxford OX3 7LF

Word count: 4,557

Corresponding author:

Natalia Calanzani

Centre for Population Health Sciences

The Usher Institute of Population Health Sciences and Informatics, University of Edinburgh

Room 123, Doorway 1, Medical School, Teviot Place, Edinburgh EH8 9AG

Tel: 0131 650 3818

Fax: 031 650 3227

Natalia.Calanzani@ed.ac.uk

For peer review only - http://bmjopen.bmj.com/site/about/guidelines.xhtml

ABSTRACT

Objectives: We aimed to test whether a brief, opportunistic intervention in general practice was a feasible and acceptable way to engage with bowel screening non-responders.

Design: This was a feasibility study testing an intervention which comprised a brief conversation during routine consultation, provision of a patient leaflet and instructions to request a replacement faecal occult blood test kit. A mixed methods approach to evaluation was adopted. Data were collected from proformas completed after each intervention, from the Bowel Screening Centre database, and from questionnaires. Semi-structured interviews were carried out. We used descriptive statistics, content and framework analysis to determine intervention feasibility and acceptability.

Participants: Bowel screening non-responders (as defined by the Scottish Bowel Screening Centre) and primary care professionals working in five general practices in Lothian, Scotland.

Primary and secondary outcome measures: Several predefined feasibility parameters were assessed, including numbers of patients engaging in conversation, requesting a replacement kit and returning it; and willingness of primary care professionals to deliver the intervention.

Results: The intervention was offered to 258 patients in five general practices: 220 (87.0%) engaged with the intervention, 60 (23.3%) requested a new kit, 22 (8.5%) kits were completed and returned. Interviews and questionnaires suggest that the intervention was feasible, acceptable, and consistent with an existing health prevention agenda. Reported challenges referred to work-related pressures, time constraints and practice priorities.

Conclusions: This intervention was acceptable and resulted in a modest increase in non-responders participating in bowel screening, although outlined challenges may affect sustained implementation. The strategy is also aligned with the increasing role of primary care in promoting bowel screening.

Keywords: feasibility studies; bowel screening; general practice; neoplasms

For peer review only - http://bmjopen.bmj.com/site/about/guidelines.xhtml

STRENGTHS AND LIMITATIONS OF THE STUDY

- This intervention is grounded in psychological theory and evidence on factors associated with non-participation in bowel screening
- Furthermore, it considered the pragmatic reality of a dynamic, time-pressured primary care environment
- This is a small-scale, non-randomised feasibility study in one region of Scotland, targeting non-responders who consult in primary care
- As the intervention is of an opportunistic nature, data on patient characteristics (such as medical history or ethnicity) are not available.
- Further limitations include not being able to record information on non-responders and ascertain how many were missed; in addition to the low participation rate among general practices invited to take part in the study.

INTRODUCTION

Bowel screening using a faecal occult blood test (FOBt) enables identification of earlier stage cancers when treatment is more likely to be beneficial [1], ultimately leading to reduction in bowel cancer mortality [2]. The United Kingdom has well-established bowel screening programmes in each of its constituent countries. The Scottish Bowel Screening Centre (SBSC) sends a guaiac-based FOBt biennially to eligible patients aged 50-74 years [3]. The current uptake is 57.7%; with lower participation among the most deprived populations compared with the least deprived groups (45.5% vs 66.6% respectively). Uptake is higher for women (60.6%) compared to men (54.7%) [4].

Both barriers to uptake and effective strategies to increase participation in bowel screening are described in the literature. Lack of awareness of bowel cancer [5] or of screening [6], concerns about unpleasantness and embarrassment [7, 8], fear of the outcome [5, 9], fatalism [10] and perception of risk [11, 12] are commonly identified barriers. Nevertheless, there is good evidence that reminders targeting patients [13, 14] and physicians [5], having one-on-one interactions/education with general practitioners (GPs) and/or nurses [6, 15] and GP endorsements [6, 16] have a positive impact on screening uptake.

Primary care has an important and increasing role in cancer prevention and cancer screening [17]. In the UK alone, a number of interventions involving primary care have been recently developed [1, 13, 16, 18, 19]. In

For peer review only - http://bmjopen.bmj.com/site/about/guidelines.xhtml

Scotland, a government programme aiming to improve cancer survival (the Detect Cancer Early Programme) [20] provided a financial incentive for practices meeting defined bowel screening targets [21, 22].

In this context, we aimed to test the feasibility and acceptability of an opportunistic intervention in general practice patient consultations, examining whether a brief conversation was a viable way to engage with non-responders and increase bowel screening participation. The study was undertaken in the Lothian region of Scotland which has slightly lower bowel screening uptake (57.2%) than the national average, and shows similar variation based on sex and socio-economic status [4].

MATERIALS AND METHODS

Recruitment of Practices and Patients

Practice recruitment

NHS Lothian provided the research team with a list of 112 practices in this region. The list had information on practice code, % screening uptake in 2013, practice list size, number of patients aged 50-75, number of average monthly non-responders, mean Scottish Index of Multiple Deprivation (SIMD) decile [23] (for those aged 50-75), and whether or not practices took part in the bowel Scottish Quality and Outcomes Framework (QOF) [21]. Eleven general practices were purposively selected for a first wave of recruitment. We oversampled among the most deprived practices with lower uptake (as it was perceived that these practices could benefit the most from the intervention), while also taking into account the other factors listed above (as these would impact on how many patients could potentially be approached during the study period). Practices were invited to take part in the study via a personalised email sent by the study's principal investigator.

A visit was scheduled at the practices that were interested in taking part in the study. A brief information session was delivered, giving background information on colorectal cancer and screening, known barriers and facilitators to screening uptake and a thorough description of the study. Practices also received a folder containing the intervention materials, study information sheet, ethical approvals, background information on bowel cancer and bowel screening and a consent form.

Estimated size of study population

A preliminary calculation estimated that each recruited general practice would have the opportunity to engage with up to 182 potentially eligible patients during the study period (Supplementary File 1). These figures are

For peer review only - http://bmjopen.bmj.com/site/about/guidelines.xhtml

rough estimates as the study findings will guide calculations of a powered sample size for a larger study. We aimed to recruit up to six general practices for testing the intervention's feasibility and acceptability.

Patient inclusion and exclusion criteria

The target population were men and women aged 50-74 registered in a participating Lothian practice who received an invitation to screening and did not return a completed kit with a definitive screening result within 90 days (i.e. the official SBSC's definition of a non-responder). Patients were excluded from the study when they lacked mental capacity as defined by the Mental Capacity Act 2005 [24] and at professionals' discretion where patients were regarded as too ill (e.g. undergoing cancer treatment or in receipt of palliative care) or distressed to take part.

Identifying bowel screening non-responders

NHS Lothian and the SBSC routinely provide Scottish general practices with a list of non-responders. The research team worked alongside each participating practice to create a customised plan to ensure they could efficiently flag non-responders in their computer systems if they did not already have a system in place.

Intervention

The intervention comprised a brief conversation about bowel screening with non-responders. During a consultation with an eligible patient, the primary care professional (PCP) (a GP, practice nurse or health care assistant) raised the topic of non-participation using neutral statements and discussed any patient concerns. A leaflet with further information and an opportunity to request a bowel screening kit (via email, phone or tear off slip with FREEPOST) was offered. The intervention was designed to last 3-5 minutes. As part of the intervention, patients could also choose whether or not to develop a written plan of how to complete and return the kit (an implementation intention) [25]. In addition to the information leaflet and FREEPOST envelope, the intervention was supported by: an A5 set of 3-4 suggested questions/topic for discussion; an intervention flowchart and guidance sheet for PCPs (Supplementary File 1).

In developing the intervention content we drew on our previous work on strategies promoting uptake of FOBt screening [26], and available literature on factors associated with uptake and barriers to screening. We also drew on psychological models, principally on Implementation Intentions [25], the Health Behaviour Framework

For peer review only - http://bmjopen.bmj.com/site/about/guidelines.xhtml

[27], and were guided by principles of motivational interviewing [28] and informed choice [29]. We chose the Health Behaviour Framework as it synthesises major health behaviour models while also considering contextual factors. It has also been successfully applied to cancer screening health behaviours [30-32]. Implementation Intentions have been associated with higher participation in studies about cervical [33], breast [34] and bowel [35] screening. Finally, motivational interviewing has been shown to increase bowel screening uptake [36, 37]; its principles aided the development of non-directive statements to discuss non-participation.

The process of developing the intervention also included eight interviews with health professionals and 19 non-responders to bowel screening to explore their views on its acceptability in a primary care setting. Overall feedback was positive and results are reported elsewhere (manuscript under review). We sought approaches (and wording in our materials) which were not coercive, but invited participants to consider the offer of screening after balancing potential benefits and harms. Materials conformed to the Scottish Bowel Screening Programme [38] and the NHS Cancer Screening Programme [39] resources and guidance.

Data Collected for Evaluation

Delivery of the intervention

PCPs logged details of each intervention on a proforma (Supplementary File 1). Researchers regularly visited practices to collect these and to distribute materials as required, recording all communication/events in an intervention log. The intervention was planned to run for 3-4 months in each practice, depending on availability.

Requests for screening kits

Requests for new kits were made to the SBSC, which logged both the requests using the tear-off slip, and the returned kits. It was not possible to identify email or telephone requests relating to this project due to the high volume received through these means daily. For the purposes of comparison, the SBSC also provided data on total number of requests for a replacement kit made by each of the recruited practices during the intervention period (plus one extra month to allow time for requests to come in), and for equivalent periods at six months, one year and two years before the intervention.

Questionnaire and Interview Data

For peer review only - http://bmjopen.bmj.com/site/about/guidelines.xhtml

For peer review only

The feasibility parameters and mechanisms to be investigated are available in Supplementary File 1. Brief end of study questionnaires were developed for primary care and bowel screening staff. The questionnaires comprised closed and open-ended questions and focused on intervention acceptability and potential impact on workload. Semi-structured interviews were carried out with members of the practice team (aiming to interview the practice manager and at least one GP or practice nurse). Interviews sought to ascertain views on the running of the brief intervention, its acceptability, and its overall feasibility as part of routine primary care.

Data analysis

Templates were created using SPSS 19 for Windows [40] to collect data on the practice proforma and the end of study questionnaires. Quantitative data from the SBSC, proformas and end of study questionnaires were analysed using descriptive statistics (summaries, frequencies and cross-tabulations). As we had a purposeful sample of practices and a non-random sample of patients, no inferential statistics were calculated [41].

End of study interviews were digitally recorded and transcribed verbatim. Qualitative data were analysed using thematic analysis informed by a framework approach including techniques of familiarisation, coding, indexing, charting, mapping and interpretation [42, 43] assisted by QSR NVivo 7 software [44]. This approach was considered appropriate due to the pre-existing feasibility parameters being tested. Identified themes were considered in the context of these parameters and interpreted according to existing theory and research. Scrutiny both within and across transcripts ensured that the analysis encompassed all perspectives and used the whole dataset. All transcripts were read by two researchers (DC and NC) and 50% were subject to triple initial coding (DC, NC and CC). The initial coding framework was developed by DC and was reiterated following discussion, with any discrepancies explored and accounted for. Content analysis [45] was used to summarise, categorise and interpret text entries made by practice staff on proformas to record reasons for consultation.

Ethical approval and consent

The study was approved by the South East Scotland Research Ethics Committee 01 (reference 14/SS/1067) and the NHS Lothian's Research and Development Office (Project Number 2014/0366). The Scottish Bowel Screening Governance Reference Group also approved the study. Written informed consent was obtained via

For peer review only - http://bmjopen.bmj.com/site/about/guidelines.xhtml

the practice manager or GP partner and separate consent was obtained for end of study interviews. Practices were reimbursed for their participation.

RESULTS

Practice recruitment

Six out of 11 invited practices consented to participate in the study; one practice subsequently withdrew due to resource issues (Supplementary file 2). The five remaining practices varied in size, bowel screening uptake and deprivation levels (Table 1). All but one practice were signed up to the Scottish QOF.

Table 1 Characteristics of recruited practices

Recruited Practices	Location	Uptake % (2013)	Pop 50-75	Mean SIMD decile (50-75 year olds)*	Average monthly non-responders	Practice list size	Signed up to QOF	Start date	End date
Practice A	Edinburgh	<45%	1,413	2.6	41	6,888	No	04/03/15	05/07/15
Practice B	Edinburgh	45-50%	2,654	3.6	61	10,440	Yes	14/04/15	15/08/15
Practice C	East Lothian	50-55%	2,515	4.5	56	8,693	Yes	22/04/15	03/09/15
Practice D	Edinburgh	50-55%	1,241	6.2	29	5,326	Yes	20/04/15	24/08/15
Practice E	Midlothian	55-60%	1,668	5.5	30	5,201	Yes	05/03/15	08/07/15

Abbreviations: Pop: population; SIMD: Scottish Index of Multiple Deprivation; QOF: Quality and Outcomes Framework.
*The Scottish Index of Multiple Deprivation (SIMD) is a measure of multiple deprivation which combines different domains related to employment, income, health, education, skills and training, geographic access to services, crime and housing [23]. The lower the decile number, the higher the deprivation levels.

Setting up the intervention

Although all practices were routinely provided with an electronic list of non-responders, there was variation in the methods in place to identify non-responders during consultations. In two practices, the researchers coded non-responders into practices' computer systems, also helping to insert screen 'pop-up' reminders in one of these cases. The remaining three practices already had systems in place.

One practice developed a digital proforma in their GP system instead of using the paper-based one provided by the research team. Planned monthly visits were not always required and were adapted to suit practice needs.

Intervention delivery, acceptance and impact on screening uptake

Overall, 258 patients were approached between March and September 2015. Men were approached slightly more often than women (53.1% vs 46.9%) and most patients were among the younger eligible age groups for

For peer review only - http://bmjopen.bmj.com/site/about/guidelines.xhtml

For peer review only

screening (median 58.00, interquartile range (IQR) 53.00-65.00) (Table 2). No information on patient ethnicity was available. The median duration of the intervention was 2.00 minutes (IQR 1.25-5.00).

Table 2. Patient and staff characteristics

Overall data	Practice A n(%)	Practice B n(%)	Practice C n(%)	Practice D n(%)	Practice E n(%)	Total n(%)
**Patient sex**						
Men	43 (60.6)	25 (45.5)	8 (28.6)	43 (61.4)	18 (52.9)	137 (53.1)
Women	28 (39.4)	30 (54.5)	20 (71.4)	27 (38.6)	16 (47.1)	121 (46.9)
Total	71 (100.0)	55 (100.0)	28 (100.0)	70 (100.0)	34 (100.0)	258 (100.0)
**Patient age**[a]						
Median	55.50	55.50	58.50	63.00	59.00	58.00
(IQR)	(53.00-64.00)	(51.75-62.25)	(52.25-67.75)	(56.25-69.00)	(53.75-63.00)	(53.00-65.00)
50-54	25 (35.7)	24 (44.4)	10 (35.7)	13 (19.1)	11 (32.4)	83 (32.7)
55-59	22 (31.4)	9 (16.7)	5 (17.9)	14 (20.6)	7 (20.6)	57 (22.4)
60-64	8 (11.4)	10 (18.5)	4 (14.3)	11 (16.2)	11 (32.4)	44 (17.3)
65-69	6 (8.6)	6 (11.1)	4 (14.3)	15 (22.1)	5 (14.7)	36 (14.2)
70-74	9 (12.9)	4 (7.4)	3 (10.7)	10 (14.7)	0 (0.0)	26 (10.2)
75-79	0 (0.0)	1 (1.9)	2 (7.1)	5 (7.4)	0 (0.0)	8 (3.1)
Total	70 (100.0)	54 (100.0)	28 (100.0)	68 (100.0)	34 (100.0)	254 (100.0)
**Interventions by primary care role**						
GP	33 (46.5)	31 (56.4)	21 (75.0)	50 (71.4)	31 (91.2)	166 (64.3)
PN	38 (53.5)	11 (20.0)	7 (25.0)	20 (28.6)	3 (8.8)	79 (30.6)
HCA	0 (0.0)	13 (23.6)	0 (0.0)	0 (0.0)	0 (0.0)	13 (5.0)
Total	71 (100.0)	55 (100.0)	28 (100.0)	70 (100.0)	34 (100.0)	258 (100.0)

Abbreviations: GP: General Practitioner; PN: Practice Nurse; HCA: Health Care Assistant.
[a]Eight patients aged 75 or older were included as their last invitation to screening happened before their 75th birthday (hence meeting eligibility criteria).
Missing data: There were no missing data for patient sex and staff carrying out the intervention. There were 4 missing cases for patient age. Percentages may not add up to 100 due to rounding.

The majority of the interventions were carried out by GPs (64.3%), followed by practice nurses (30.6%) and health care assistants (5.0%). Patients receiving the intervention consulted for a variety of reasons (Supplementary File 3). The main reasons were reviews of existing conditions (22.0%), consultations due to musculoskeletal symptoms/conditions (13.2%), to carry out tests or obtain test results (11.6%), or due to respiratory or ear, nose and throat symptoms/conditions (8.8%).

The majority of patients who were offered the intervention accepted it (i.e. engaged in conversation) (87.0%), with variations across practices (Table 3). The leaflet was given to 74.2% of patients. Almost a quarter of the 258 patients approached (n=60) requested a replacement kit using a reply slip. Over a third of these patients (n=22) also returned a completed kit.

For peer review only

For peer review only - http://bmjopen.bmj.com/site/about/guidelines.xhtml

Table 3. Intervention acceptability, requested and returned kits

Overall data		Practice A n(%)	Practice B n(%)	Practice C n(%)	Practice D n(%)	Practice E n(%)	Total n(%)
Duration of intervention (minutes)[a]	Median (IQR)	3.50 (2.00-5.00)	2.00 (2.00-4.50)	2.00 (1.00-5.00)	2.00 (2.00-3.25)	1.00 (1.00-2.00)	2.00 (1.25-5.00)
Acceptance of intervention	Accepted (yes)	56 (78.9)	46 (85.2)	24 (96.0)	61 (88.4)	33 (97.1)	220 (87.0)
	Leaflet given (yes)	40 (57.1)	44 (81.5)	24 (85.7)	48 (68.6)	34 (100.0)	190 (74.2)
	Leaflet completed in practice (yes)	10 (14.3)	9 (16.7)	0 (0.0)	19 (27.9)	0 (0.0)	38 (17.3)
Requested kits using a slip	N requested kits/total interventions (% requesting a kit amongst total interventions)	16/71 (22.5)	7/55 (12.7)	6/28 (21.4)	25/70 (35.7)	6/34 (17.7)	60/258 (23.3)
Returned kits	N completed kits returned/total requested kits (% completing kits amongst total requests)	1/16 (6.3)	4/7 (57.1)	5/6 (83.3)	8/25 (32.0)	4/6[c] (66.7)	22/60 (36.7)
Test results	Negative	1	3	4	8	4	20
	Positive	0	0	1	0	0	1
	Pending[b]	0	1	0	0	0	1
Non-responders approached who became a responder to screening	N completed kits/N approached non-responders (% approached who became a responder)	1/71 (1.4)	4/55 (7.2)	5/28 (17.9)	8/70 (11.4)	4/34 (11.8)	22/258 (8.5)
Non-responders accepting the intervention who became a responder to screening	N completed kits/N accepting intervention (% accepting intervention who became a responder)	1/56 (1.8)	4/46 (8.7)	5/24 (20.8)	8/61 (13.1)	4/33 (12.1)	22/220 (10.0)

Abbreviations: IQR: Interquartile Range.
Missing data: there were 10 missing cases for duration of intervention, 5 for whether intervention was accepted, 2 for whether leaflet was given and 4 for whether it was completed in the practice. The same denominator (i.e. the total number of interventions carried out) applies for each question about acceptance of the intervention (i.e. intervention accepted, leaflet given and leaflet completed in practice) due to issues observed in data entry. Overall 4 leaflets were given although intervention was ticked as not accepted and 12 leaflets were completed in practice although they were ticked as not given to the patient.
[a]Over 90% of the interventions (n=225) lasted up to 5 minutes.
[b]A weak positive result (not shown) indicates that further tests are needed; in one case results for further tests were not yet available so results are shown as pending. In another case a weak positive became a positive result after further tests.
[c]One patient from Practice E requested a kit but was not sent one as s/he was only due for a new test in 2016. The National Bowel Screening System (BoSS) does not allow for sending additional kits for patients who are not due for another test; this helps to avoid over-screening.

For peer review only - http://bmjopen.bmj.com/site/about/guidelines.xhtml

Younger participants were more likely to accept the intervention (median age 58.00; IQR 53.00-64.75 for those accepting the intervention vs. 64.00; IQR 57.00-71.50 for those not accepting it). Men refused the intervention more often than women (the former represented 66.7% of all refusals). Over half (57.6%) of refused interventions were carried out by a practice nurse (Supplementary File 3).

Descriptive data from the Bowel Screening Centre on requested kits (Supplementary File 4) show that there was an increase in the number of requested kits across all practices during the intervention period (the highest increase in practice D and the lowest in Practice E) compared to two years, one year and six months prior to the intervention.

End of study evaluation: qualitative interviews

Eleven individual and one group in-depth qualitative interviews were conducted with a total of 14 primary care staff (four GPs, four practice nurses, five practice managers and one health care assistant). Thirteen interviews were face to face and one was via telephone. Findings from the qualitative interviews identified four main domains: the primary care and general *health care context*; the *processes* involved *in delivering the intervention*; *patient-related acceptability*; and *primary care professional acceptability* (Table 4).

For peer review only - http://bmjopen.bmj.com/site/about/guidelines.xhtml

Table 4. End of study interview quotes to support findings

<u>1.Health care context</u>

Existing practices

- We remind patients about cervical screening, so it's on a, sort of, slightly similar vein." Practice Nurse, Practice D
- "I think similar to our alcohol brief interventions and I think it enables us to initiate conversation…about an important subject which we might not otherwise do." GP, Practice D

Pressurised work environment

- "Because of the state of the practice at the minute when we've got doctors leaving - retiring and resigning – it's just put an added burden on existing people to do that." Practice Manager, Practice C
- "Just part of a greater workload issue. We're struggling to provide our contracted services, so I'm not going to commit to take on anything now unless it's properly resourced." GP, Practice E

Acknowledging barriers to screening

- "But it's always practicalities. That's why people don't want to do it." Practice Nurse, Practice D
- It's interesting because the research does show there is a kind of gradient there and that in some minority groups the uptake is not as high." GP, Practice D

Knowledge of and attitude to bowel screening

- It's definitely an extra to add in to the patients but I'm a real proponent of preventive health care and I think these things are worthwhile." GP, Practice D
- "To be honest, the practice population that we have have far bigger problems than whether they did their bowel screening or not, so in the real world it's possibly not one of the things we would include." Practice Nurse, Practice A

<u>2. Processes in implementing the intervention</u>

Providing information on the intervention

- "Actually it was very helpful, because I hadn't understood what the patients were being asked to do." GP, Practice E
- "They all thought it was a very worthwhile thing to sign up to." Practice Manager, Practice C

Appropriate timing and scenarios for the intervention

- "Probably if you have had any consultations that has presented with six problems and the last thing you need is to get into something else." GP, Practice B
- "I think when you leave it to the end you have not encroached on the patient's time." Practice Nurse, Practice B

Use of intervention supporting materials

- "You maybe look at it once or twice and see what's the kind of chat and then you probably don't dig it out every time, it's so opportunistic. GP, Practice A

Minimising paperwork, adaptations and integrating the intervention into existing IT

- "Obviously if we had a reminder for everything […] then we wouldn't be able to see the screen for reminders. So it's okay in the short term but in the long term it's a bit more difficult." Practice Manager, Practice A
- "We've got a computer, so it tells you that you need a bowel intervention, so why (not) record the data that you wanted on the same system?" GP, Practice E

Time limitations

- "I was aware that we were missing lots of people as the GPs simply didn't have time." Practice Manager, Practice C
- "In GP land when you've got ten minutes, ten minutes, ten minutes, then every little five minutes counts." GP, Practice A

Constraints in implementing the intervention

- "It all boils down to sometimes some men don't want to discuss it. […] I've found that sometimes a barrier, especially with older men." Practice Nurse, Practice D
- "We have a high Asian population and they are not keen to talk about poo or the practicalities of keeping their kit beside the toilet." GP, Practice D

Translating intention into action

- "When you actually spoke about it they thought it was a good idea, "I'll do it", but whether they do it or not, don't know." Practice Nurse, Practice B

For peer review only - http://bmjopen.bmj.com/site/about/guidelines.xhtml

3. Patient factors and acceptability

Patient receptivity

- "I had no bad experiences at all. People were happy to talk about it." Practice Nurse, Practice E
- "I was surprised at how receptive the patients were to it." GP, Practice B

Patient awareness and support for bowel screening

- "They knew pretty much what was involved." Practice Nurse, Practice E
- My own finding was as soon as you mentioned it to patients and brought up screening, the majority of them were keen to go ahead and do it." GP, Practice B

Patient priorities and motivation to participate in bowel screening

- "The patient would say, well that's the least of my concerns and I'll tell you why…" GP, Practice A
- "Just sort of, inertia and couldn't be bothered, not a priority." GP, Practice E

4. Primary care professional factors and acceptability

Acceptability to professionals

- "It isn't an onerous thing to do and what they have to do is fairly straightforward." Practice Manager, Practice E
- "It's just an extension of normal dialogues really.[…] I think it's entirely appropriate and problem, well almost problem free.[…] It was quick and simple to do and if the feedback turns out to that it's effective, then I think it would be an appropriate thing to implement in practice." GP, Practice E

Professional interest, variable support and priority

- "I think there was a bit of a mixed response. I think generally GPs when they're asked to do something over and above are just like whoa, we're totally overwhelmed." GP, Practice A
- "I mean they are so busy here. They are always running late […] so whether or not it just hasn't been a great priority for them." Practice Nurse, Practice E

Motivation to adopt the intervention

- "Bowel screening is effective and we didn't have to sell that concept to them. […] I think if they think it's a good thing they're more likely to advocate it." Practice Manager, Practice B
- "Sometimes it felt like quite a positive thing to do because it is about health promotion and disease prevention and that very much chimes with our ethos." GP, Practice A

The intervention as part of a broader preventive health agenda

- "I think we have to be trying to educate people to look after themselves instead of fixing things after they're broken." Practice Nurse, Practice A
- "Giving them a message of empowering them to take control of their destiny, which is something I think that is really lacking in a population like ours." GP, Practice A

The perceived professional role in educating patients and raising awareness

- "It was a good opportunity to bring it to the forefront of their consciousness […] to kind of put some medical opinion behind it and say, "this is the reason we are doing it", you know. It does reduce your chances of having a serious bowel cancer if we catch it early." GP, Practice D
- It was just talking round the practicalities. […] "Oh, I just didn't know how to do it" […] so you'd try to talk through it a little bit with them." Practice Nurse, Practice D

Potential for differing roles and involvement

- "I'm a more junior practice nurse, people aren't coming to me with loads of things, […] so maybe I have more time to look at it." Practice Nurse, Practice E
- "I do think that the nurses will integrate it more than the GPs will. […] I think GPs deal with the more acute problems, whereas health checks you've maybe got a bit more time and people are more relaxed and they are expecting you to ask that." Practice Manager, Practice A

For peer review only

Health care context

PCPs reported that certain organisational aspects of primary care services impacted on the implementation of the brief intervention. Existing health promotion interventions already placed demands on practices. PCPs emphasised the highly pressured primary care work environment with a cumulative impact on their ability to commit to new projects.

PCPs also highlighted important barriers to bowel screening participation such as embarrassment and practical issues, in addition to the influence of gender and ethnicity. There was variation by practitioner and also across practices, reflecting PCP's knowledge and belief in screening and also particular patient populations (with reported constraints such as illiteracy and high levels of deprivation).

Processes in delivering the intervention

PCPs commented on the usefulness of the background information on bowel cancer and screening to increase their understanding and belief in screening and the intervention, fostering a sense of commitment. In relation to delivering the intervention itself, staff commented on appropriate types of consultations to raise the topic of screening, appropriate timing within the consultation, frequency of use of the intervention materials, adaptations made, and issues around logging interventions. Staff also referred to use of computer systems to highlight non-responders (also serving as a reminder) and to log and monitor interventions, as well as the need to minimise paperwork and integrate any future interventions into existing computer systems. Constraints raised in ability to deliver the intervention related to difficulty in interacting on the topic of bowel screening with certain groups (e.g. males and minority ethnic groups), but mainly to limited time in an already pressured environment.

Patient factors and acceptability

PCPs reported that patients were positive or neutral but rarely negative when engaging on the topic of screening. They felt that patients were overall receptive to the intervention and discussing bowel screening. In a number of cases, patients had knowledge of bowel cancer and bowel screening and were aware of the benefits of taking part, but there was a large degree of perceived 'inertia', where bowel screening did not appear to be a priority.

For peer review only - http://bmjopen.bmj.com/site/about/guidelines.xhtml

Primary care professional factors and acceptability

The PCPs reported the importance of increasing bowel screening participation and found the process to be acceptable, straightforward and easy to administer as part of routine consultations. However, PCPs reported variation depending on factors such as special interests, personal experience, perceived priorities for the patient population, and forced priorities as a result of limited time and work pressures. Professionals also acknowledged the influence of their attitude towards bowel screening on their approach to the intervention. Those who were motivated drew on the importance of screening and their belief in a holistic approach to health care whereas for others bowel screening was not the highest priority in order to improve patient care.

Interviewees were cognisant of their role in the intervention process in educating patients about bowel cancer and screening and raising awareness of the benefits of participating, and how sometimes this alone was enough to prompt patients to take part. However, they felt they lacked control once the patient had left the consulting room over whether or not they ultimately returned a FOBt kit. There was also discussion of the most appropriate member of the practice team to take the intervention forward, whether this be related to time available, role (GP, PN or HCA), practice load or special interest.

Practice staff reported it was feasible to roll out the intervention and made suggestions for certain adjustments to make it more effective, such as handing out kits directly to patients; streamlining any written materials and making them electronic; integrating any data recording into existing computer systems; and considering funding and set time periods dedicated to bowel screening that complement other initiatives.

End of study evaluation: questionnaires

Nineteen PCPs returned a completed questionnaire (response rate 38.8%). Thirteen were GPs, five were practice nurses and one was a practice manager. This group carried out over half (51.2%) of all interventions (n=132). As reflected in the qualitative interviews, all but one GP (no recorded interventions) stated that most patients were receptive to the intervention; that it could be easily incorporated into practice; and they would theoretically be willing to take part in the study again. Nonetheless, despite positive feedback, ten professionals highlighted lack of time as a potential or actual barrier.

For peer review only - http://bmjopen.bmj.com/site/about/guidelines.xhtml

Four bowel screening staff (out of seven; three screening officers and the screening supervisor) whom had been involved in the intervention returned a completed end of study questionnaire. They all stated that the intervention could be easily incorporated into their workload. However, opinion on the potential impact on workload was uncertain. Three respondents stated that it was difficult identifying calls from patients in intervention practices among over 300 daily calls. However, all four reported that it was suitable for testing in a larger study in its present form.

DISCUSSION

Summary

Results indicate that the intervention was feasible and acceptable to PCPs and patients (as reported by professionals). Of those reached, a small but important minority became responders, a likely underestimate as email and telephone requests were not recorded. It is also possible that some patients may have completed their original kit at home and returned it. The majority of patients approached were willing to discuss the subject of bowel screening. Some patients may have made an informed choice not to participate in screening (indeed informed choice guided the intervention design), although this was not documented in this study.

Qualitative and questionnaire data indicate that the intervention was straightforward and easy to implement and reflected similar ongoing health promotion initiatives, and was thus an effective way to communicate with patients about bowel screening. Overall, PCPs were willing and felt comfortable delivering the intervention in different scenarios, suggesting suitability for most primary care consultations. Practices varied in the number of patients approached and reasons for this variability were widely described in the interviews. Inappropriate or challenging scenarios reported included those involving patients with complex health and social care needs, poor literacy, English as a second language, or sensitivities related to ethnicity and culture. Evidence on appropriate scenarios can help inform future interventions on how to approach these hard to reach groups. Furthermore, these findings have implications for the flexible design of the intervention at a larger scale so it meets the needs of individual practices and different patient groups.

PCPs stated that materials were helpful to promote the intervention, draw attention to it and reinforce messages post-consultation. Nonetheless, not all practices had systems in place to identify non responders,

For peer review only - http://bmjopen.bmj.com/site/about/guidelines.xhtml

or wished to use reminders long-term; both issues can influence the success of any future implementation. Finally, feedback from the SBSC regarding the intervention was also positive, but future implementation would need to take into account the difficulty recoding telephone and email requests and explore all potential mechanisms for requests to be made.

Study strengths and limitations

This was an evidence-based intervention informed by current data on non-participation and psychological theory, grounded in the pragmatic reality of the primary care workload. A good relationship with practices was developed. The study produced a clear audit trail and the duplicate coding of qualitative data helped ensure consistency, rigour and transparency. Nevertheless, this was a small feasibility study which requires further evaluation in larger patient populations. The study also targeted patients who consult in primary care; those who do not consult may present different challenges regarding participation. As we had to adapt to a dynamic, time-pressured primary care environment, it was not possible to record information about all eligible non-responders. It is unknown how many of them consulted (and how many of these were approached). Some elements of the intervention were adapted by practices, this is expected in a complex intervention [46]. In fact, an intervention that can be adapted to local circumstances without loss of its essence is a strength that facilitates practical implementation.

The number of patients approached was smaller than our estimates based on national population statistics. This may be due to variation in general practice characteristics and the actual number of non-responders who consulted in primary care. We may have underestimated the number of possible appropriate consultations, although qualitative data suggest that the number of perceived inappropriate scenarios were small. Furthermore, time pressures were described as the main reason for not approaching patients, even among professionals motivated to carry out the intervention. Nonetheless, we consider that an increase in requested bowel screening kits in all recruited practices was a positive outcome influenced by the intervention, and that more patients could be reached over time if the intervention was incorporated into practice, similar to what happens with other types of brief interventions.

Five out of the 11 invited practices completed the study. This is a low participation rate (45.5%), but not uncommon due to recognised challenges in doing research in primary care [47, 48]. Reasons given for non-

For peer review only - http://bmjopen.bmj.com/site/about/guidelines.xhtml

participation were struggles with demand/targets and pressures in primary care, a challenge also described by those who took part in the study. Only one of our recruited practices was within the 30% most deprived areas in Scotland (three were amongst the 50% most deprived). Hence, it is likely that these areas were underrepresented (this would need to be explored in a larger study). To ensure better representation, further intervention roll-out should consider additional strategies to engage with practices in the most deprived areas (such as more personal contact and additional monetary incentives). Nonetheless, it is important to recognise that certain challenges may be beyond the scope of the study, and that priorities in these practices may be different.

Finally, the context of governmental campaigns in Scotland promoting screening participation [49] and the QOF rewards may have influenced practice decisions to participate in our study. It also made it harder to separate the impact of the campaigns and the intervention on patients' behavioural response.

Comparison with other studies

Brief interventions in primary care are well established and successful in influencing behaviours such as alcohol consumption [50], tobacco smoking [51], and weight management [52]. Our research also contributes to a body of recent UK studies examining primary care-based interventions to influence screening behaviour and demonstrating their effectiveness in improving bowel screening uptake [13, 18, 19], offering further evidence on the benefits of such interventions. Our results show that intervention acceptance varied across practices with the two most deprived practices having the lowest proportion of acceptance and the lowest number of kits requested and returned. This finding suggests that GP endorsement alone is not sufficient to change patient bowel screening behaviour among the most deprived groups, as reflected in a recent study [16].

PCPs reported lack of control after the patient leaves the consulting room - indeed only over a third of those requesting a kit actually returned it - and a gap between intention and action, a phenomenon well described in the literature [53, 54]. The implementation intention plan aimed to help deal with this limitation but was seldom used by PCPs and other studies have shown mixed effectiveness [33, 35, 55-57].

Implications for practice and further research

For peer review only - http://bmjopen.bmj.com/site/about/guidelines.xhtml

Our feasibility parameters did not include a cost-related analysis. Future roll-out should aim to incorporate both direct costs (such as professional time) and indirect costs for the SBSC. These costs need to be balanced against long-term gains in terms of early detection and likely reduction of population mortality [58, 59].

Most interventions were carried out by a GP, which is consistent with national statistics in Scotland demonstrating that GPs carry out about two-thirds of all consultations in primary care [60]. A higher proportion of people seen by a nurse did not accept the intervention. Disease monitoring was a common reason for seeing a nurse, reflecting official data on consultation patterns in Scotland [60]. Interviews show that both GPs and PNs saw their role as important. PNs suggested that they may have more time to deliver interventions incorporated into routine patient checks and reviews, but there was some suggestion that GPs placed greater emphasis on educating and persuading patients. HCAs also reported being in a good position to deliver interventions, though the numbers in this study were small. There is scope to explore further the potential differing roles for members of the primary care team in this context, and to identify ways for different professionals to have a more active involvement.

Practices varied in the number of patients approached. Our qualitative data suggest that the practice population profile, staff's level of engagement with screening, professional knowledge, experience and interests are likely to have been strong explanatory elements. Understanding this variability has implications for the flexible design of the intervention at a larger scale so it meets the needs of individual practices and different patient groups.

The primary care context was described as a highly pressured environment comprising complex patients' needs, limited financial and human resources, increasing patients but diminishing staff, and the need to incentivise health promotion. When asked about the likelihood of continuing on with the intervention, it was clear that despite perceiving it as useful and supporting its underlying ethos, other pressing issues would be prioritised. These challenges constrain the ability to deliver and sustain the intervention, irrespective of motivation, willingness and recognised importance. However, the flexibility of the intervention meant that it could be adapted to suit individual practices and demonstrated an impact on bowel screening participation despite the outlined constraints.

For peer review only - http://bmjopen.bmj.com/site/about/guidelines.xhtml

CONCLUSIONS

We tested a primary care-based intervention to increase uptake in non-responders to FOBt screening, and found it to be feasible and acceptable in Scottish primary practices, despite recognised organisational and system constraints that would need to be considered for the intervention to be more widely implemented. Further testing in a randomised controlled trial would give robust evidence of the effectiveness of the brief intervention in increasing informed screening participation. The intervention can be useful as one tool to complement other efforts to engage with non-responders and reflects the broader aims from the Scottish government to raise awareness and normalise bowel screening. Our study adds to evidence that primary care can play a key role in promoting bowel screening uptake.

AUTHORS' CONTRIBUTIONS

NC, DC, GV, SO, RJCS, SS, JP, DW and CC contributed to the design of the study. LB contributed to the acquisition of data for the work. NC, DC, GV, SO, RJCS, LB, SS, JP, DW and CC contributed to interpretation of data for the work. CC, DW, DC and NC were involved in recruitment, data collection and data analysis. NC, DC, GV, SO, RJCS, LB, SS, JP, DW and CC contributed to the drafting and revision of the manuscript and the approval of the final version. NC, DC, GV, SO, RJCS, LB, SS, JP, DW and CC agree to be accountable for all aspects of the work in ensuring that questions related to the accuracy or integrity of any part of the work are appropriately investigated and resolved.

FUNDING STATEMENT

This work was supported by CRUK [grant reference number C12357/A13965] through the National Awareness and Early Diagnosis Initiative (NAEDI-2). The funder had no role in the design, collection, analysis, and interpretation of data; in the writing of the manuscript; and in the decision to submit the manuscript for publication.

CONFLICT OF INTEREST STATEMENT

The authors declare no conflicts of interest.

DATA SHARING STATEMENT

For peer review only - http://bmjopen.bmj.com/site/about/guidelines.xhtml

Some of the unpublished data are available from the authors (such as intervention materials and questionnaires). The corresponding author (natalia.calanzani@ed.ac.uk) can be contacted by anyone interested in accessing these. Data from patients, primary care practices and the Scottish Bowel Screening Centre cannot be accessed by anyone who is not part of the research team due to ethical and confidentiality concerns.

ACKNOWLEDGEMENTS

Bowel screening data were obtained from the Scottish Bowel Screening Centre in Dundee; we thank all staff for their assistance and for completing the end of study questionnaires. We acknowledge the support and advice from NHS Lothian, which provided the practice lists used for recruiting practices and the help from NHS Scotland which provided the leaflets in Polish. We thank Dr Robert Scully for his help with medical acronyms/terms in the intervention proformas and Dr Ewan Gray for his advice when revising this manuscript. We also thank the reviewers for their comments and recommendations. We are very grateful to each GP, practice nurse, health care assistant and practice manager who agreed to take part in the study, gave up their time to be interviewed and completed the end of study questionnaires.

REFERENCES

[1] Damery S, Smith S, Clements A, et al. Evaluating the effectiveness of GP endorsement on increasing participation in the NHS Bowel Cancer Screening Programme in England: study protocol for a randomized controlled trial. *Trials* 2012;13:18.

[2] Hewitson P, Glasziou Paul P, et al. Screening for colorectal cancer using the faecal occult blood test, Hemoccult. *Cochrane Database Syst Rev*: John Wiley & Sons, Ltd, 2007.

[3] Bowel Cancer UK. Screening in the Nations. 2015. Available from: http://www.bowelcanceruk.org.uk/understanding-bowel-cancer/screening/screening-in-the-uk/.

[4] Scottish Bowel Screening Programme. Scottish Bowel Screening Programme. Key Performance Indicators Report: May 2015 data submission. Invitations between 1st November 2012 and 31st October 2014. https://www.isdscotland.org/Health-Topics/Cancer/Publications/2015-08-04/2015-08-04-Bowel-Screening-KPI-Full-Report.xlsx, 2015

[5] European Commission. European guidelines for quality assurance in colorectal cancer screening and diagnosis. Luxembourg: Publications Office of the European Union, 2010.

For peer review only - http://bmjopen.bmj.com/site/about/guidelines.xhtml

[6] Holden DJ, Harris R, Porterfield DS, et al. Enhancing the use and quality of colorectal cancer screening. *Evid Rep Technol Assess* 2010:1-195.

[7] Jepson R, Clegg A, Forbes C, et al. The determinants of screening uptake and interventions for increasing uptake: a systematic review. *Health Technol Assess* 2000;4:i-vii,1-133.

[8] Palmer CK, Thomas MC, von Wagner C, et al. Reasons for non-uptake and subsequent participation in the NHS Bowel Cancer Screening Programme: a qualitative study. *Br J Cancer* 2014;110:1705-11.

[9] Chapple A, Ziebland S, Hewitson P, et al. What affects the uptake of screening for bowel cancer using a faecal occult blood test (FOBt): a qualitative study. *Soc Sci Med* 2008;66:2425-35.

[10] Power E, Miles A, von Wagner C, et al. Uptake of colorectal cancer screening: system, provider and individual factors and strategies to improve participation. *Future Oncol* 2009;5:1371-88.

[11] Malila N, Senore C, Armaroli P. European guidelines for quality assurance in colorectal cancer screening and diagnosis. First Edition--Organisation. *Endoscopy* 2012;44 Suppl 3:Se31-48.

[12] Powe BD, Faulkenberry R, Harmond L. A review of intervention studies that seek to increase colorectal cancer screening among African-Americans. *Am J Health Promot* 2010;25:92-9.

[13] Wardle J, von Wagner C, Kralj-Hans I, et al. Effects of evidence-based strategies to reduce the socioeconomic gradient of uptake in the English NHS Bowel Cancer Screening Programme (ASCEND): four cluster-randomised controlled trials. *Lancet* 2015;387:751-59.

[14] Brouwers MC, De Vito C, Bahirathan L, et al. Effective interventions to facilitate the uptake of breast, cervical and colorectal cancer screening: an implementation guideline. *Implement Sci* 2011;6:112.

[15] Sabatino SA, Lawrence B, Elder R, et al. Effectiveness of interventions to increase screening for breast, cervical, and colorectal cancers: nine updated systematic reviews for the guide to community preventive services. *Am J Prev Med* 2012;43:97-118.

[16] Raine R, Duffy SW, Wardle J, et al. W. Impact of general practice endorsement on the social gradient in uptake in bowel cancer screening. *Br J Cancer* 2016;114:321-6.

[17] Rubin G, Berendsen A, Crawford SM, et al. The expanding role of primary care in cancer control. *Lancet Oncol* 2015;16:1231-72.

[18] Hewitson P, Ward AM, Heneghan C, et al. D. Primary care endorsement letter and a patient leaflet to improve participation in colorectal cancer screening: results of a factorial randomised trial. *Br J Cancer* 2011;105:475-80.

For peer review only

For peer review only - http://bmjopen.bmj.com/site/about/guidelines.xhtml

[19] Shankleman J, Massat NJ, Khagram L, et al. Evaluation of a service intervention to improve awareness and uptake of bowel cancer screening in ethnically-diverse areas. *Br J Cancer* 2014;111:1440-7.

[20] The Scottish Government. Detect Cancer Early. 2015. http://www.gov.scot/Topics/Health/Services/Cancer/Detect-Cancer-Early

[21] ISD Scotland, National Services Scotland. Quality and Outcomes Framework. Prevalence, achievement, payment and exceptions data for Scotland, 2013/2014. https://isdscotland.scot.nhs.uk/Health-Topics/General-Practice/Publications/2014-09-30/2014-09-30-QOF-Report.pdf?44355410338, 2014

[22] The Scottish Government. Detect Cancer Early - Bowel Screening Initiative. Health and Social Care Integration Directorate Primary Care Division, 2013.

[23] The Scottish Government. Scottish Index of Multiple Deprivation 2012. 2012. http://simd.scotland.gov.uk/publication-2012/.

[24] Department for Constitutional Affairs. Mental Capacity Act 2005 Code of Practice. London, 2007. https://www.gov.uk/government/uploads/system/uploads/attachment_data/file/497253/Mental-capacity-act-code-of-practice.pdf.

[25] Gollwitzer PM. Goal Achievement: The Role of Intentions. *Eur Rev Soc Psychol* 1993;4:141-85.

[26] Campbell C, Techer L, Crawford F, et al Primary Care based strategies to increase uptake of colorectal cancer screening. USA: Report to the National Cancer Institute, 2009.

[27] Bastani R, Glenn BA, Taylor VM, et al. Integrating theory into community interventions to reduce liver cancer disparities: The Health Behavior Framework. *Prev Med* 2010;50:63-7.

[28] Treasure J. Motivational interviewing. *Adv Psychiatr Treat* 2004;10:331-7.

[29] Jepson RG, Hewison J, Thompson A, et al. Patient perspectives on information and choice in cancer screening: a qualitative study in the UK. *Soc Sci Med* 2007;65:890-9.

[30] Tu SP, Yip MP, Chun A, et al. Development of intervention materials for individuals with limited English proficiency: lessons learned from "Colorectal Cancer Screening in Chinese Americans". *Med Care* 2008; 46:S51-61.

[31] Jo AM, Maxwell AE, Wong WK, et al. Colorectal cancer screening among underserved Korean Americans in Los Angeles County. *Journal Of Immigrant And Minority Health* 2008; 10:119-26.

[32] Maxwell AE, Bastani R, Crespi CM, et al. Behavioral mediators of colorectal cancer screening in a randomized controlled intervention trial. *Prev Med* 2011; 52:167-73.

For peer review only - http://bmjopen.bmj.com/site/about/guidelines.xhtml

[33] Sheeran P, Orbell S. Using implementation intentions to increase attendance for cervical cancer screening. *Health Psychol* 2000; 19:283-9.

[34] Rutter D, Quine L, Steadman L, et al. Increasing Attendance at Breast Cancer Screening: Field Trial. Final Report to NHSBSP. University of Kent. Department of Psychology. Centre for Research in Health Behaviour, 2007.

[35] Greiner KA, Daley CM, Epp A, et al. Implementation Intentions and Colorectal Screening: A Randomized Trial in Safety-Net Clinics. *Am J Prev Med* 2014; 47:703-14.

[36] Ling BS, Schoen RE, Trauth JM, et al. Physicians encouraging colorectal screening: a randomized controlled trial of enhanced office and patient management on compliance with colorectal cancer screening. *Arch Intern Med* 2009; 169:47-55.

[37] Lasser KE, Murillo J, Medlin E, et al. A multilevel intervention to promote colorectal cancer screening among community health center patients: results of a pilot study. *BMC Family Practice* 2009; 10:1-7.

[38] NHS Inform. Risks and benefits of bowel screening. 2013. Available from: . https://www.nhsinform.scot/healthy-living/screening/bowel-screening/introduction-to-bowel-screening#benefits-and-risks

[39] NHS Cancer Screening Programmes. Consent to Cancer Screening. Cancer Screening Series No 4., 2008.

[40] IBM SPSS Statistics for Windows, Version 19.0. Armonk, NY: IBM Corp.: IBM Corp, 2010.

[41] Baker R, Brick MJ, Bates NA, Battaglia M, Couper MP, Dever JA, K.J. G, Tourangeau R. Report of the AAPOR Task Force on Non-Probability Sampling. 2013. Available from: https://www.aapor.org/AAPOR_Main/media/MainSiteFiles/NPS_TF_Report_Final_7_revised_FNL_6_22_13.pdf

[42] Gale N, Heath G, Cameron E, et al. Using the framework method for the analysis of qualitative data in multi-disciplinary health research. *BMC Med Res Methodol* 2013; 13:117.

[43] Ritchie J, Spencer L. Qualitative data analysis for applied policy research. In: Bryman A, Burgess R, editors. Analyzing qualitative data. Abindgon: Routledge, 1994:173-94.

[44] NVivo qualitative data analysis Software. QSR International Pty Ltd, 2006.

[45] Krippendorf K. Content analysis: An introduction to its methodology. Beverly Hills, CA: Sage Publications, 1980.

For peer review only - http://bmjopen.bmj.com/site/about/guidelines.xhtml

[46] Medical Research Council. Developing and evaluating complex interventions: new guidance. Available from: http://www.mrc.ac.uk/documents/pdf/complex-interventions-guidance/, 2008.

[47] Baird B, Charles A, Honeyman M, et al. Understanding pressures in general practice. The King's Fund, 2016:1-100.

[48] Wilson S, Delaney BC, Roalfe A, et al. Randomised controlled trials in primary care: case study. *BMJ* 2000; 321:24.

[49] The Scottish Government. New bowel cancer home screening test. 2015. http://news.gov.scot/news/new-bowel-cancer-home-screening-test#downloads

[50] Kaner EF, Beyer F, Dickinson HO, et al. Effectiveness of brief alcohol interventions in primary care populations. *Cochrane Database Syst Rev* 2007:Cd004148.

[51] Bauld L, Bell K, McCullough L, et al. The effectiveness of NHS smoking cessation services: a systematic review. *J Public Health* 2010; 32:71-82.

[52] Aveyard P, Lewis A, Tearne S, et al. Screening and brief intervention for obesity in primary care: a parallel, two-arm, randomised trial. *Lancet* 2016;388:2492–500.

[53] Sheeran P. Intention—Behavior Relations: A Conceptual and Empirical Review. *Eur Rev Soc Psychol* 2002; 12:1-36.

[54] Power E, Van Jaarsveld CH, McCaffery K, et al. Understanding intentions and action in colorectal cancer screening. *Ann Behav Med* 2008; 35:285-94.

[55] Rutter D, Quine L, Steadman L, et al. Increasing Attendance at Breast Cancer Screening: Field Trial. Final Report to NHSBSP. University of Kent. Department of Psychology. Centre for Research in Health Behaviour, 2007.

[56] Lo SH, Good A, Sheeran P, et al. Preformulated implementation intentions to promote colorectal cancer screening: a cluster-randomized trial. *Health Psychol* 2014; 33:998-1002.

[57] Walsh JC. Increasing Screening Uptake for a Cervical Smear Test: Predictors of Attendance and the use of Action Plans in Prior Non-Attenders. *The Irish Journal of Psychology* 2005; 26:65-73.

[58] Sharp L, Tilson L, Whyte S, et al. Cost-effectiveness of population-based screening for colorectal cancer: a comparison of guaiac-based faecal occult blood testing, faecal immunochemical testing and flexible sigmoidoscopy. *Br J Cancer* 2012; 106:805-16.

For peer review only - http://bmjopen.bmj.com/site/about/guidelines.xhtml

[59] Birtwistle M, Earnshaw A. Saving lives, averting costs. An analysis of the financial implications of achieving earlier diagnosis of colorectal, lung and ovarian cancer Incisive Health on behalf of Cancer Research UK, 2014.

[60] ISD Scotland. Practice Team Information (PTI) Annual Update (2012/13). Available from: http://www.isdscotland.org/Health-Topics/General-Practice/Publications/2013-10-29/2013-10-29-PTI-Report.pdf, 2013.

For peer review only

For peer review only - http://bmjopen.bmj.com/site/about/guidelines.xhtml

Supplementary file 1:

- **A. Estimating the size of the study population**
- **B. Intervention supporting materials**
- **C. Feasibility parameters**

A. Estimating the size of the study population

According to the 2011 Census, there are 184,846 residents in Lothian aged 50-74[1] (the age range for eligibility to bowel cancer screening). In these age groups, approximately 86% of patients (estimated 158,968) consult at least once a year (aged 45 and over are included as data were not available separately)[2], with a linear increase in the number of consultations according to patients' age (from 5.2 annual consultations to 7.7 among the most deprived groups).
As of September 2013 there were 127 practices in Lothian[3]. Each practice would then have, on average, at least 1252 patients consulting during our intervention period (although numbers vary between practices).

Official Scottish Bowel screening data for the period 2011-2013 show that uptake in Lothian was 55.3% (ranging from 39.6% to 59.8% from the most deprived to the least deprived areas)[4]. Considering the participation rates for the most deprived areas (39.6%) and the population aged 50-74; there would roughly be a maximum of 756 eligible patients who could be reached during the study period per practice, or 4536 in six selected practices.

In practice, however, numbers are likely to be much smaller. For example, in 2011 the prevalence of cancer (all types) in Scotland was 4.5%[5]. These patients would not take part in the intervention as it would not be appropriate to approach the issue of cancer screening to someone in receipt of cancer treatment. Considering cancer prevalence in Scotland, the number of eligible patients would be reduced to 4355 in all practices. This is a conservative estimate as cancer is more common in areas of high deprivation (which is the case of some of the selected practices in Lothian). Furthermore, the opportunity to approach a patient about screening may not arise in every consultation. We estimate that an opportunity will arise in about a quarter of all consultations with eligible participants, thus reducing the number of patients to 1089 (or approximately 182 per practice).

References:

1.Scotland's Census 2011 - National Records of Scotland. Table DC1117SC - Age by sex. All people [Internet]. 2014. Available from: http://www.scotlandscensus.gov.uk.

2.Estimated number of patients seen1/not seen2 in Scotland by either a General Practitioner (GP) or Practice Nurse [Internet]. 2013. Available from: http://www.isdscotland.org/PTI/.

3 .General Practice – GP workforce and practice population statistics to 2013. Scottish general practice characteristics: as at 30th September from 2005 to 2013. In: ISD Scotland, editor. 2014.

4.Scottish Bowel Screening Programme. Scottish Bowel Screening Programme. Key Performance Indicators Report: May 2014 data submission. Invitations between 1st November 2011 and 31st October 2013. Scotland: Scottish Bowel Screening Programme; 2014.

5. ISD Scotland. Cancer in Scotland: ISD Scotland; 2012. Available from: https://isdscotland.scot.nhs.uk/Health-Topics/Cancer/Publications/2012-04-24/Cancer_in_Scotland_summary_m.pdf

B. Intervention supporting materials

Intervention material	Aim(s)	Theoretical framework/adopted principles
3-4 questions/statements<br>• A5 coloured laminated sheet of paper<br>• Given to the health care professional	• To guide discussion addressing concerns around or barriers to bowel screening with non-responders	• Non-directive statements<br>• Non-coercion<br>• Informed choice<br>• Implementation Intentions theory
Staff flowchart<br>• A4 coloured laminated sheet of paper<br>• Given to the health care professional	• To describe barriers to screening (with examples), and evidence-based approaches to deal with these barriers	• Health Behaviour Framework<br>• Motivational Interviewing
Patient leaflet and freepost envelope<br>• A4 coloured sheet of paper folded into A5, perforated so a reply slip could be sent back by post<br>• FREEPOST envelope addressed to the bowel screening centre<br>• Given to patients who accepted the intervention	• To deconstruct health beliefs associated with low uptake, i.e. the barriers described in the flowchart<br>• To provide space for the patient to develop their own implementation plan<br>• To offer the opportunity to request a new test kit	• Health Behaviour Framework<br>• Non-coercion<br>• Informed choice<br>• Implementation Intentions theory
Guidance sheet<br>• A4 coloured laminated sheet of paper<br>• Given to the health care professional	• To provide practice staff with relevant information regarding the research study	N/A
Practice Proforma<br>• Green A4 sheet with perforations (3 proformas in each)<br>• Given to the health care professional<br>• Designed to require approximately 2 minutes to be completed	• To collect relevant intervention data so the feasibility outcomes could be assessed:<br>○ Intervention date, staff name and role, duration of the intervention, patient age and gender, reasons for consultation (text)<br>○ Whether intervention was accepted, leaflet was given and completed in practice<br>○ Comments	N/A

Note: Bowel Screening leaflets in Polish were requested from NHS Scotland and provided to practices with a larger Polish population

For peer review only

C. Feasibility parameters

- Processes required in general practice to record (and flag) responder status in patients' electronic records
- Number of patients approached during consultations
- Number of interventions delivered over the intervention period
- Length of time the intervention takes
- Number and willingness of patients to engage in conversation when responder status is raised during a consultation
- Number of patients who were willing to receive leaflet
- Number of patients who completed leaflet with health care professional
- Number of patients who sent reply slip
- Number of patients who returned a completed kit
- Processes required in the bowel screening centre to deal with the extra-to-programme returned FOBt kits, and to ensure over-screening does not occur
- Whether the brief intervention leads to longer discussion with patients regarding cancer screening, or bowel symptoms
- Willingness of GPs and practice nurses to deliver intervention
- Overall views on the study's feasibility according to primary care professionals
- Overall views on the study's feasibility according to bowel screening staff

For peer review only

Supplementary file 2

A. Recruitment Flowchart
B. Characteristics of practices approached in Wave 1

A. Recruitment Flowchart

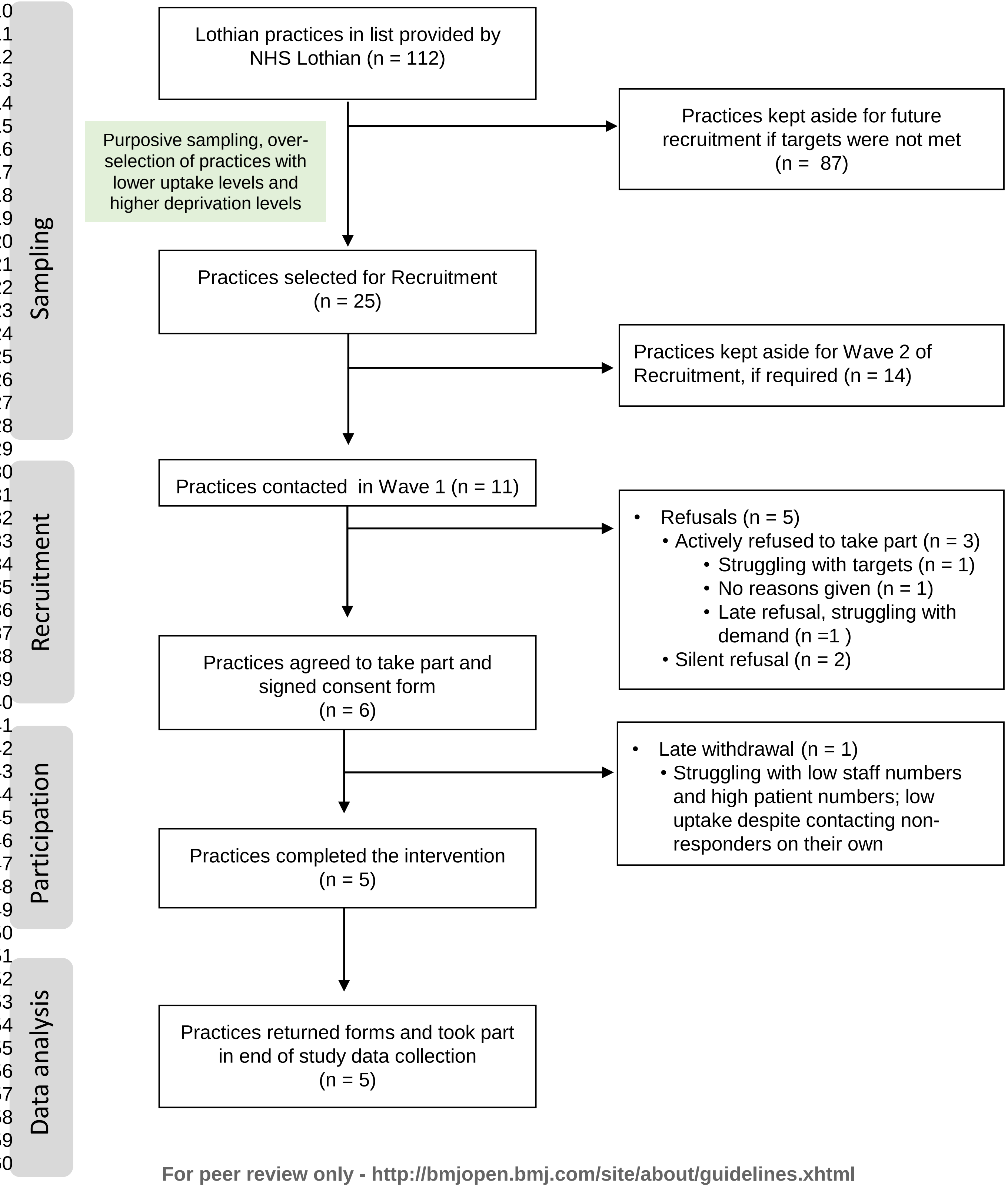

B. Characteristics of practices approached in Wave 1

Practice name	Outcome in Wave 1 of recruitment	Uptake % (2013)	Pop 50-75	Mean SIMD decile* (50-75yr olds)	Average Monthly Non Responder Numbers	Practice list size (ISD)
**Practice A**	Recruited	<45%	1,413	2.6	41	6,888
**Practice B**	Recruited	45-50%	2,654	3.6	61	10,440
**Practice C**	Recruited	50-55%	2,515	4.5	56	8,693
**Practice D**	Recruited	50-55%	1,241	6.2	29	5,326
**Practice E**	Recruited	55-60%	1,668	5.5	30	5,201
**Practice F**	Recruited, but withdrew as struggling with pressures	50-55%	3,444	3.2	73	11,624
**Practice G**	Actively refused, struggling with pressures	<45%	2,543	2.1	70	12,482
**Practice H**	Actively refused, struggling with pressures	45-50%	2,030	4.2	47	9,585
**Practice I**	Actively refused, no reasons given	50-55%	3,809	4.6	76	13,984
**Practice J**	Silent refusal	<45%	1,075	3.9	29	7,848
**Practice K**	Silent refusal	45-50%	2,241	3.4	50	8,287

Abbreviations: ISD: Information Services Division; Pop: population; SIMD: Scottish Index of Multiple Deprivation.
*The lower the decile number, the higher the deprivation levels.

Supplementary file 3:

A. Recorded reasons for consultation
B. Comparing patients who accepted and did not accept the intervention

A. Recoded reasons for consultation

Reasons for consultation	Practice A n(%)	Practice B n(%)	Practice C n(%)	Practice D n(%)	Practice E n(%)	All practices n(%)			
	All	All	All	All	All	GP	PN	HCA	All
Known chronical illness/review of existing condition	25 (36.8)	6 (11.5)	2 (7.4)	18 (26.1)	4 (11.8)	12 (21.8)	40 (72.7)	3 (5.5)	55 (22.0)
Musculoskeletal symptoms/conditions	3 (4.4)	7 (13.5)	7 (25.9)	11 (15.9)	5 (14.7)	33 (100.0)	0 (0.0)	0 (0.0)	33 (13.2)
Tests/test results	8 (11.8)	8 (15.4)	3 (11.1)	8 (11.6)	2 (5.9)	4 (13.8)	17 (58.6)	8 (27.6)	29 (11.6)
Respiratory or ENT symptoms/conditions	6 (8.8)	7 (13.5)	2 (7.4)	4 (5.8)	3 (8.8)	14 (63.6)	8 (36.4)	0 (0.0)	22 (8.8)
Prescriptions/medication review	7 (10.3)	2 (3.8)	1 (3.7)	1 (1.4)	4 (11.8)	15 (100.0)	0 (0.0)	0 (0.0)	15 (6.0)
Skin complaints	1 (1.5)	5 (9.6)	2 (7.4)	3 (4.3)	4 (11.8)	13 (86.7)	2 (13.3)	0 (0.0)	15 (6.0)
Non-clinical	2 (2.9)	4 (7.7)	2 (7.4)	5 (7.2)	1 (2.9)	10 (71.4)	2 (14.3)	2 (14.3)	14 (5.6)
Gastrointestinal symptoms/conditions	3 (4.4)	4 (7.7)	1 (3.7)	4 (5.8)	2 (5.9)	14 (100.0)	0 (0.0)	0 (0.0)	14 (5.6)
Multiple issues	4 (5.9)	1 (1.9)	0 (0.0)	1 (1.4)	5 (14.7)	11 (100.0)	0 (0.0)	0 (0.0)	11 (4.4)
Mental health symptoms/conditions	0 (0.0)	2 (3.8)	2 (7.4)	2 (2.9)	1 (2.9)	7 (100.0)	0 (0.0)	0 (0.0)	7 (2.8)
Preventative behaviour	4 (5.9)	1 (1.9)	2 (7.4)	0 (0.0)	0 (0.0)	0 (0.0)	7 (100.0)	0 (0.0)	7 (2.8)
Gynaecological or urological symptoms/conditions	2 (2.9)	1 (1.9)	0 (0.0)	1 (1.4)	0 (0.0)	4 (100.0)	0 (0.0)	0 (0.0)	4 (1.6)
Inconclusive	0 (0.0)	0 (0.0)	0 (0.0)	2 (2.9)	0 (0.0)	2 (100.0)	0 (0.0)	0 (0.0)	2 (0.8)
Other/non-specific symptoms	3 (4.4)	4 (7.7)	3 (11.1)	9 (13.0)	3 (8.8)	21 (95.5)	1 (4.5)	0 (0.0)	22 (8.8)
Total	68 (100.0)	52 (100.0)	27 (100.0)	69 (100.0)	34 (100.0)	N/A	N/A	N/A	250 (100.0)

Abbreviations: GP: General Practitioner; PN: Practice Nurse; HCA – Health Care Assistant; ENT – ear, nose and throat
Missing data: 8 cases (3 in Practice A, 1 in Practice C, 1 in Practice D and 3 in Practice B).
Sums may not add up to 100 due to rounding

For peer review only

B. Comparing patients who accepted and did not accept the intervention

Overall data	Accepted the intervention (n=220) n(%)	Did not accept the intervention (n=33) n(%)	Overall (n=253)* n(%)
**Patient sex**			
Male	113 (51.4)	22 (66.7)	135 (53.4)
Female	107 (48.6)	11 (33.3)	118 (46.6)
**Patient age**			
Median (IQR)	58.00 (53.00-64.75)	64.00 (57.00-71.50)	58.00 (53.00-65.00)
50-54	78 (35.5)	3 (9.1)	81 (32.5)
55-59	48 (21.8)	9 (27.3)	57 (22.9)
60-64	36 (16.4)	6 (18.2)	42 (16.9)
65-69	32 (14.5)	3 (9.1)	35 (14.1)
70-74	17 (7.7)	9 (27.3)	26 (10.4)
75-79	5 (2.3)	3 (9.1)	8 (3.2)
**Staff carrying out the intervention**			
GP	153 (69.5)	11 (33.3)	164 (64.8)
PN	57 (25.9)	19 (57.6)	76 (30.0)
HCA	10 (4.5)	3 (9.1)	13 (5.1)
**Duration of the intervention**			
Median (IQR)	2.00 (1.00-5.00)	2.00 (2.00-5.00)	2.00 (1.00-5.00)
**Reasons for consultation**			
Known chronical illness/review of existing condition	42 (19.7)	12 (37.5)	54 (22.0)
Musculoskeletal symptoms/conditions	30 (14.1)	3 (9.4)	33 (13.5)
Tests/test results	22 (10.3)	5 (15.6)	27 (11.0)
Respiratory or ENT symptoms/conditions	17 (8.0)	5 (15.6)	22 (9.0)
Prescriptions/medication review	15 (7.0)	0 (0.0)	15 (6.1)
Skin complaints	15 (7.0)	0 (0.0)	15 (6.1)
Non-clinical	14 (6.6)	0 (0.0)	14 (5.7)
Gastrointestinal symptoms/conditions	12 (5.6)	1 (3.1)	13 (5.3)
Multiple issues	10 (4.7)	1 (3.1)	11 (4.5)
Mental health symptoms/conditions	6 (2.8)	1 (3.1)	7 (2.9)
Preventative behaviour	5 (2.3)	1 (3.1)	6 (2.4)
Gynaecological or urological symptoms/conditions	2 (0.9)	2 (6.3)	4 (1.6)
Inconclusive	2 (0.9)	0 (0.0)	2 (0.8)
Other/non-specific symptoms	21 (9.9)	1 (3.1)	22 (9.0)

Abbreviations: GP: General Practitioner; PN: Practice Nurse; HCA – Health Care Assistant; ENT – ear, nose and throat; IQR – Interquartile range
*In 5 cases data were missing on whether patient accepted the intervention. These cases are not included here; hence the overall values do not match the ones in the main manuscript (i.e. 258 participants). There were also missing data for patient age (4 cases), duration of intervention (9 cases) and reasons for consultation (8 cases). Sums may not add up to 100 due to rounding.

For peer review only

Supplementary file 4

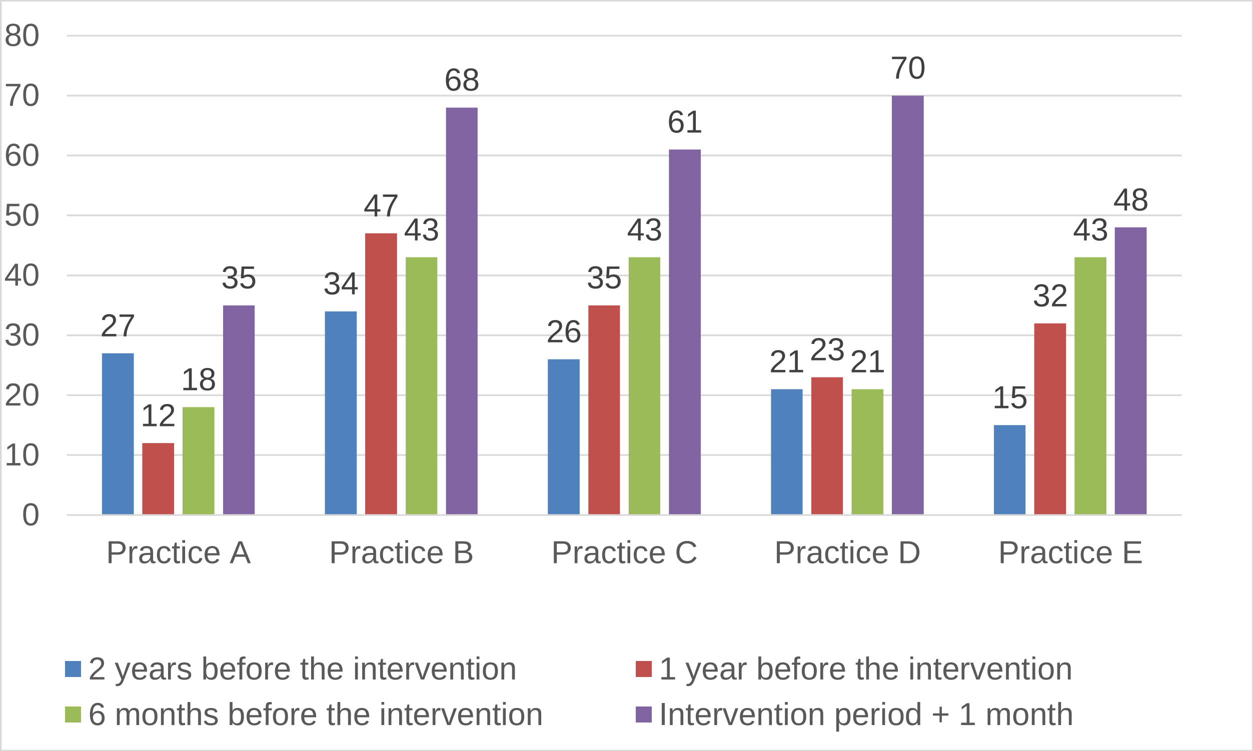

For peer review only

STROBE 2007 (v4) Statement—Checklist of items that should be included in reports of *cross-sectional studies*

Section/Topic	Item #	Recommendation	Reported on page #
**Title and abstract**	1	(*a*) Indicate the study's design with a commonly used term in the title or the abstract	1
		(*b*) Provide in the abstract an informative and balanced summary of what was done and what was found	2
**Introduction**			
Background/rationale	2	Explain the scientific background and rationale for the investigation being reported	3,4
Objectives	3	State specific objectives, including any prespecified hypotheses	4
**Methods**			
Study design	4	Present key elements of study design early in the paper	5,6
Setting	5	Describe the setting, locations, and relevant dates, including periods of recruitment, exposure, follow-up, and data collection	4,5,8
Participants	6	(*a*) Give the eligibility criteria, and the sources and methods of selection of participants	4,5
Variables	7	Clearly define all outcomes, exposures, predictors, potential confounders, and effect modifiers. Give diagnostic criteria, if applicable	6
Data sources/ measurement	8*	For each variable of interest, give sources of data and details of methods of assessment (measurement). Describe comparability of assessment methods if there is more than one group	7
Bias	9	Describe any efforts to address potential sources of bias	Limitations and potential sources of bias approached in the discussion (page 17)
Study size	10	Explain how the study size was arrived at	4,5
Quantitative variables	11	Explain how quantitative variables were handled in the analyses. If applicable, describe which groupings were chosen and why	7
Statistical methods	12	(*a*) Describe all statistical methods, including those used to control for confounding	7
		(*b*) Describe any methods used to examine subgroups and interactions	N/A
		(*c*) Explain how missing data were addressed	Reported in all cases (table footnotes on pages 9 and 10, supplementary file 3). N/A for multivariate analysis as only descriptive statistics were reported
		(*d*) If applicable, describe analytical methods taking account of sampling strategy	N/A

For peer review only

		(*e*) Describe any sensitivity analyses	N/A
**Results**			
Participants	13*	(a) Report numbers of individuals at each stage of study—eg numbers potentially eligible, examined for eligibility, confirmed eligible, included in the study, completing follow-up, and analysed	8, 9, 10, 11 and supplementary file 3
		(b) Give reasons for non-participation at each stage	Not available, limitations approached on pages 17-19
		(c) Consider use of a flow diagram	Supplementary file 2
Descriptive data	14*	(a) Give characteristics of study participants (eg demographic, clinical, social) and information on exposures and potential confounders	8, 9, 10, 11, supplementary file 3, limitations discussed in pages 17-19
		(b) Indicate number of participants with missing data for each variable of interest	Reported in all cases (table footnotes on pages 9 and 10, supplementary file 3).
Outcome data	15*	Report numbers of outcome events or summary measures	8, 9, 10, 11
Main results	16	(*a*) Give unadjusted estimates and, if applicable, confounder-adjusted estimates and their precision (eg, 95% confidence interval). Make clear which confounders were adjusted for and why they were included	N/A, no multivariate analyses were carried out
		(*b*) Report category boundaries when continuous variables were categorized	N/A
		(*c*) If relevant, consider translating estimates of relative risk into absolute risk for a meaningful time period	N/A
Other analyses	17	Report other analyses done—eg analyses of subgroups and interactions, and sensitivity analyses	Supplementary file 3
**Discussion**			
Key results	18	Summarise key results with reference to study objectives	16
Limitations	19	Discuss limitations of the study, taking into account sources of potential bias or imprecision. Discuss both direction and magnitude of any potential bias	17-19
Interpretation	20	Give a cautious overall interpretation of results considering objectives, limitations, multiplicity of analyses, results from similar studies, and other relevant evidence	16-19
Generalisability	21	Discuss the generalisability (external validity) of the study results	17-19
**Other information**			
Funding	22	Give the source of funding and the role of the funders for the present study and, if applicable, for the original study on which the present article is based	20

*Give information separately for cases and controls in case-control studies and, if applicable, for exposed and unexposed groups in cohort and cross-sectional studies.

Note: An Explanation and Elaboration article discusses each checklist item and gives methodological background and published examples of transparent reporting. The STROBE checklist is best used in conjunction with this article (freely available on the Web sites of PLoS Medicine at http://www.plosmedicine.org/, Annals of Internal Medicine at http://www.annals.org/, and Epidemiology at http://www.epidem.com/). Information on the STROBE Initiative is available at www.strobe-statement.org.

For peer review only
